# Diagnostic value of endoscopic ultrasound for insulinoma localization: A systematic review and meta-analysis

**DOI:** 10.1371/journal.pone.0206099

**Published:** 2018-10-23

**Authors:** Hao Wang, Ying Ba, Qian Xing, Jian-ling Du

**Affiliations:** Department of Endocrinology, The First Affiliated Hospital of Dalian Medical University, Dalian, Liaoning Province, China; University of Texas Southwestern Medical Center at Dallas, UNITED STATES

## Abstract

**Background:**

Previous studies reported varies parameters of endoscopic ultrasound (EUS) for the localization of insulinomas, the purpose of this meta-analysis based on published studies to accuracy the diagnostic value of EUS.

**Methods:**

PubMed, Embase, Web of science, Cochrane library and Wanfang digital database were searched to identify published studies up to April 2018, which diagnostic insulinoma by using EUS. Retrieved sensitivity, specificity, positive likelihood ratio (PLR), negative likelihood ratio (NLR), diagnostic odds ratio (DOR) and receiver operating characteristic (ROC) curves data were summarized for meta-analysis.

**Results:**

A total of 9 studies involved a total of 350 patients were included in final analysis. The summary sensitivity, specificity, PLR, and NLR were 0.81 (95%CI: 0.75–0.86), 0.90 (95%CI: 0.84–0.94), 7.90 (95%CI: 4.9–12.8), and 0.21 (95%CI: 0.16–0.29), respectively. Further, the pooled DOR was 37.00 (95%CI:19.55–70.04) and area under the ROC was 0.92 (95%CI: 0.90–0.84).

**Conclusion:**

The findings of this study demonstrate that EUS should be a routine diagnosis approach for the preoperative localization of insulinomas.

## Introduction

Insulinoma is the most common neuroendocrine neoplasm, which account for 1–10 new cases per million annual incidence worldwide [1]. The clinical manifest of insulinoma included varying degrees of hypoglycemia symptoms, including fasting symptoms of hypoglycemia, low blood glucose level, and symptomatic relief with glucose administration. This disease significantly affect the quality of life and even caused life-threatening events. Therefore, early diagnosis and treatment of insulinoma are critical. Currently, surgical removal of the neoplasm is the primary procedure in treatment of insulinoma, and preoperative insulinoma localization is necessary [2,3].

Insulinoma is usually small and benign, while high accuracy diagnostic method remains unclear [4–6]. Traditional imaging methods including ultrasound, magnetic resonance imaging, and computed tomography-negative were difficult accuracy localization of insulinoma. Although high accuracy rate of digital subtraction angiography and arterial stimulation of venous blood test for localization of insulinoma, while these two procedures were invasive and determined by expert technical skills. Nowadays, endoscopic ultrasonography (EUS) is widely used for diagnosis of insulinoma, which associated with minimally invasive and could acquired high accuracy rate. However, the diagnostic parameters are varies among previous studies. We therefore conducted this meta-analysis to evaluate the diagnostic value of EUS for localization of insulinoma, and to compare the diagnostic parameters according to study and patients characteristics.

## Methods

### Data sources, search strategy, and selection criteria

Our present study was performed in accordance with the guidelines for the Preferred Reporting Items for Systematic reviews and Meta-Analyses (PRISMA) [7]. We performed electronic searches in PubMed, Embase, Web of Science, Cochrane Library and Wanfang digital database through April 2018 to identify studies of EUS in diagnosis in localization of insulinoma. The following search terms were used: endoscopic ultrasound, endoscopic ultrasonography, insulinoma, localization, and pancreatic tumor. The applications of search strategy in various databases were as follows: PubMed:("Endosonography"[Mesh]) AND "Insulinoma" [Mesh]); Embase: Emtree term-exploded = (insulinoma AND endoscopic ultrasound); Web of Science: TS = (insulinoma AND endoscopic ultrasound); Cochrane Library and WanFang: keyword = (insulinoma AND endoscopic ultrasound). The additional publications in reference lists and citation sections of recovered articles were also searched. There were no restricted on publication status and publication language.

The literature search, and study selection were independently undertaken by 2 authors, and any inconsistencies were resolved by the primary author until a consensus was reached. Studies were included if the study met the following criteria: (1) Patients satisfied the symptoms of having Whipple triad and were suspected of having insulinomas; (2) Insulinoma patients were confirmed by postoperative pathology; and (3) the study provided true positive, false positive, false negative, true negative. The exclusion criteria included (1) Lack of postoperative pathology. (2) Incomplete clinical data. (3) un-extractable four-fold table. (4) duplicate reports. (5) Animal experiments.

### Data collection and quality assessment

Two authors reviewed the abstract first independently and then summarized the full selected studies. Any inconsistencies were settled by group discussion until a consensus was reached. The relevant data abstracted are listed as follows: first author’s name, publication year, country, study design, gold standard, apparatus, methods, true and false positive and negative. Quality Assessment of Diagnostic Accuracy Studies (QUADAS-2) tool was used to evaluate the quality of the studies included in this meta-analysis independently by the two authors [8,9]. This method consisted of 4 components: selection of cases, trials to be assessed, gold standard, as well as flowchart and progress of cases. Each of the assessment has seven items and response as “yes”, “no”, or “uncertainty”. The answer of “yes” means that a study’s risk bias can be judged as low, while “no” and “uncertainty” mean that the risk of bias can be judged as high.

### Statistical analysis

Revman5.3 was used for quality assessment, while other analysis was conducted using Stata software. The sensitivity, specificity, PLR, NLR and corresponding 95% confidence intervals (CIs) were calculated from true positive, false positive, false negative, and true negative, which was extracted from each study before data pooling. The bivariate random effects [10] was applied to summarize sensitivity, specificity, PLR, NLR, and the hierarchical regression model was used to summary receiver operating characteristic (SROC) curve and the area under the ROC [11]. Q statistic and I-square were used to estimate the heterogeneity of individual study contributing to the pooled estimate. *P >*0.10 indicated no significant heterogeneity, while *P ≤*0.10 indicated significant heterogeneity for the Q statistic [12,13]. A fixed-effect model was used if there was low heterogeneity between the studies (P> 0.05, I-square<50%) and a random effect model was used if the heterogeneity was high (P< 0.05, I-square > 50%).Meta-regression analyses were conducted for DOR on the basis of study design, year of publication, enrolled population, ethnicity and diagnostic methods [14]. Deeks’ asymmetry test was used to evaluate potential publication bias [15]. All reported P values are two-sided, and p <0.05 was considered statistically significant for pooled diagnostic parameters.

## Results

**Fig 1:**
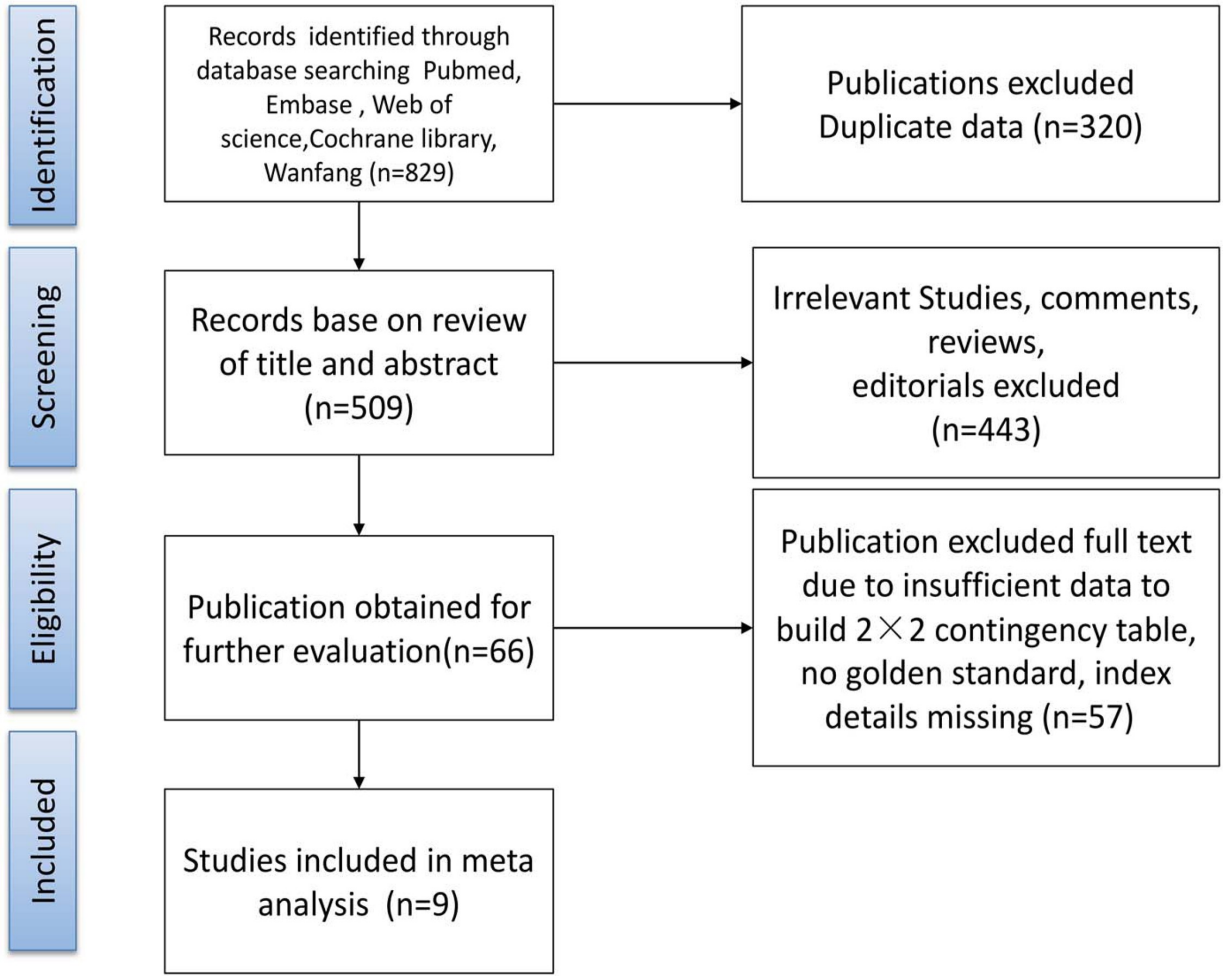
Retrieval flowchart to obtain study data for meta-analysis.

The results of the study-selection process are shown in Fig 1. The initial electronic research identified 829 articles, of which 320 articles were excluded due to duplication. Further 443 articles were excluded due to irrelevance (removed after reading the titles and abstracts). A total of 66 potentially eligible studies were selected; after reviewing the full text of each study and browsing the results, 57 articles were excluded due to incomplete data, lacking of gold standards, and incomplete description of the trials (removed after reading the full manuscript). Finally, 9 studies with a total of 350 patients were included in this systematic review and meta-analysis [16–24]. The characteristics of the included studies are listed in Table 1. The included studies published between 1992–2009. Four studies were conducted in Europe [16–19], 2 studies were conducted in USA or Brazil [20,21], and the remaining 3 studies were conducted in Asia [22–24]. Four of included studies with prospective design [16,19,23,24], and the remaining 5 studies with retrospective design [17,18,20–22].

**Fig 2:**
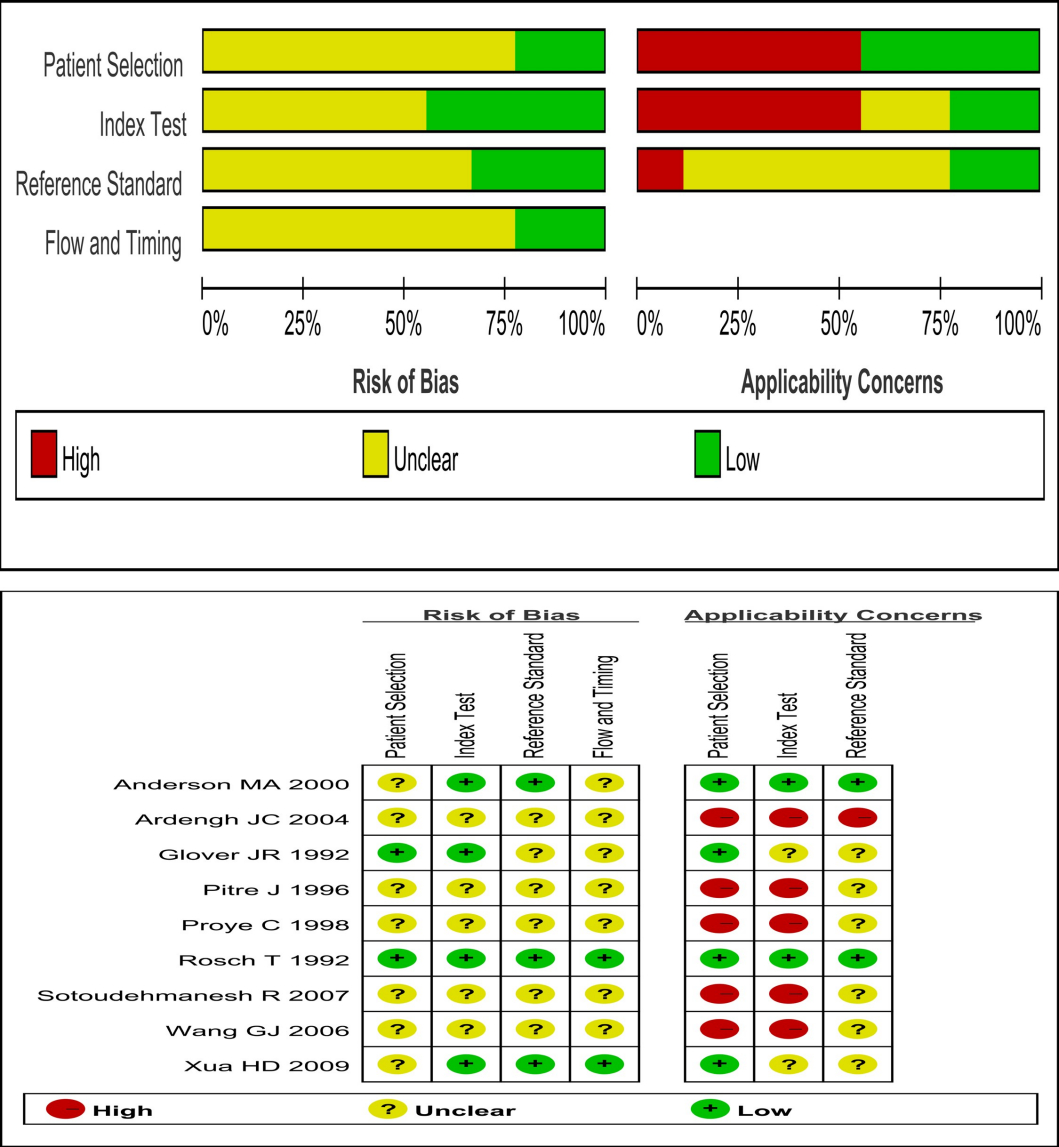
Quality assessments for the included studies.

The quality assessment results of the included studies are shown in Fig 2.

**Fig 3:**
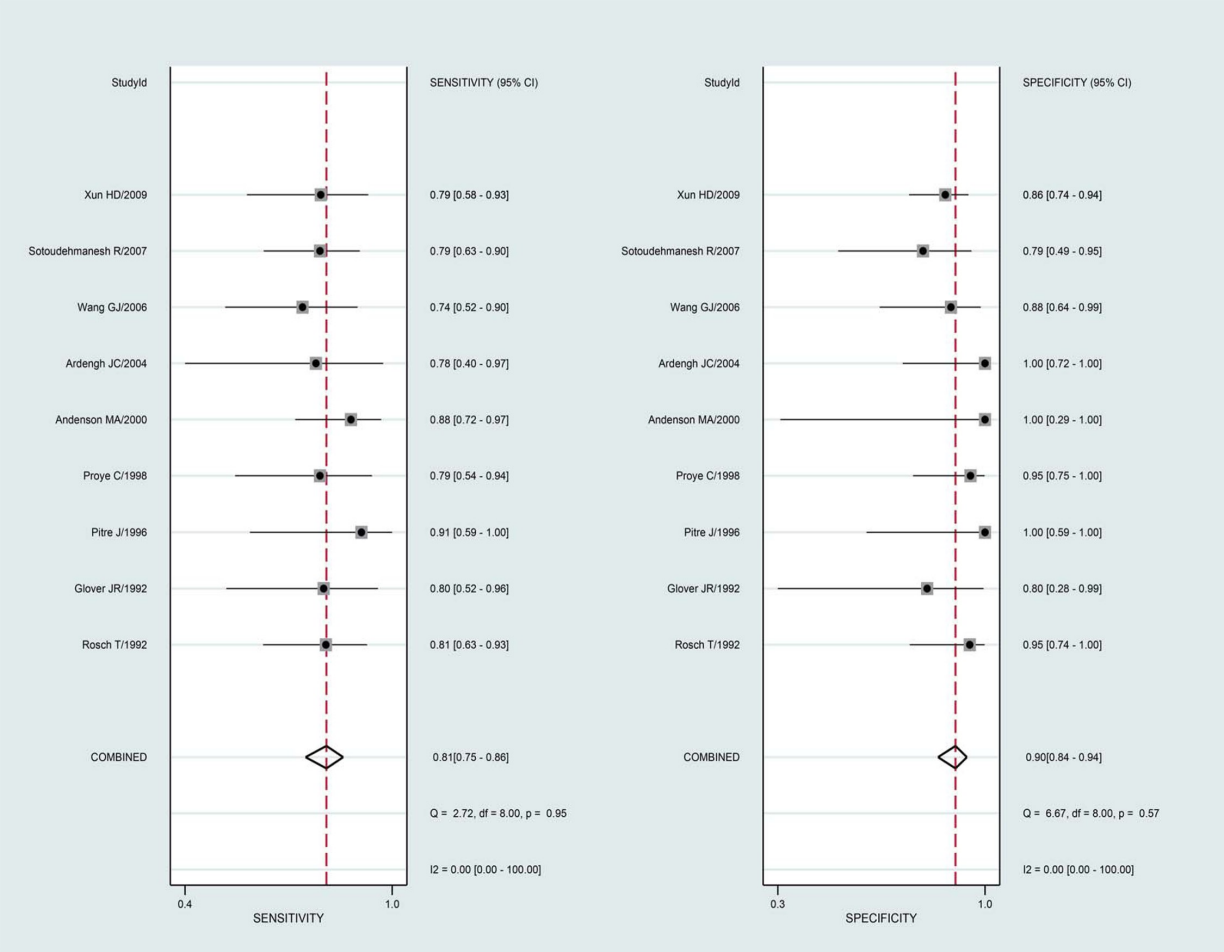
Forest plots for sensitivity and specificity.

**Fig 4:**
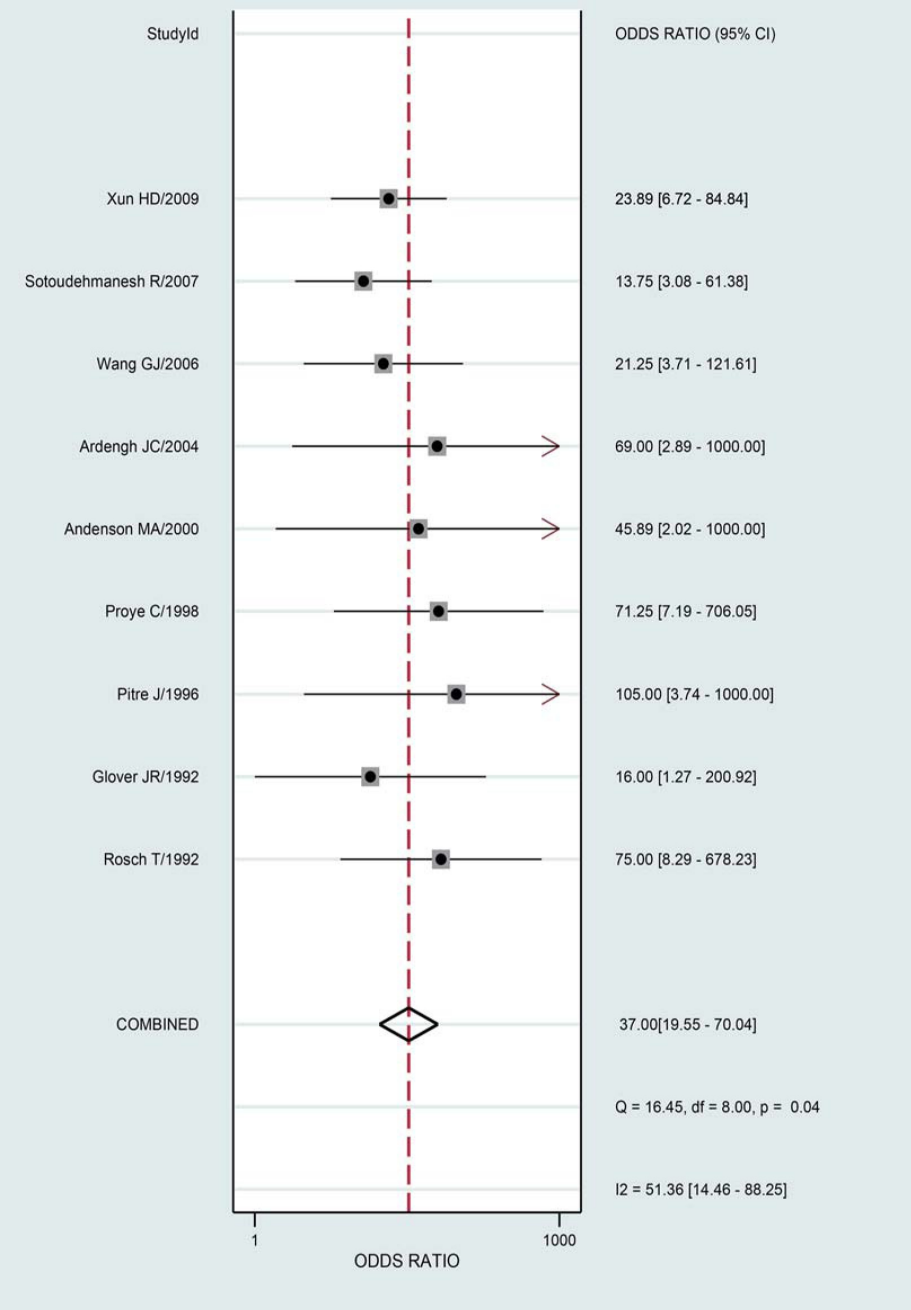
Forest plot for DOR.

**Fig 5:**
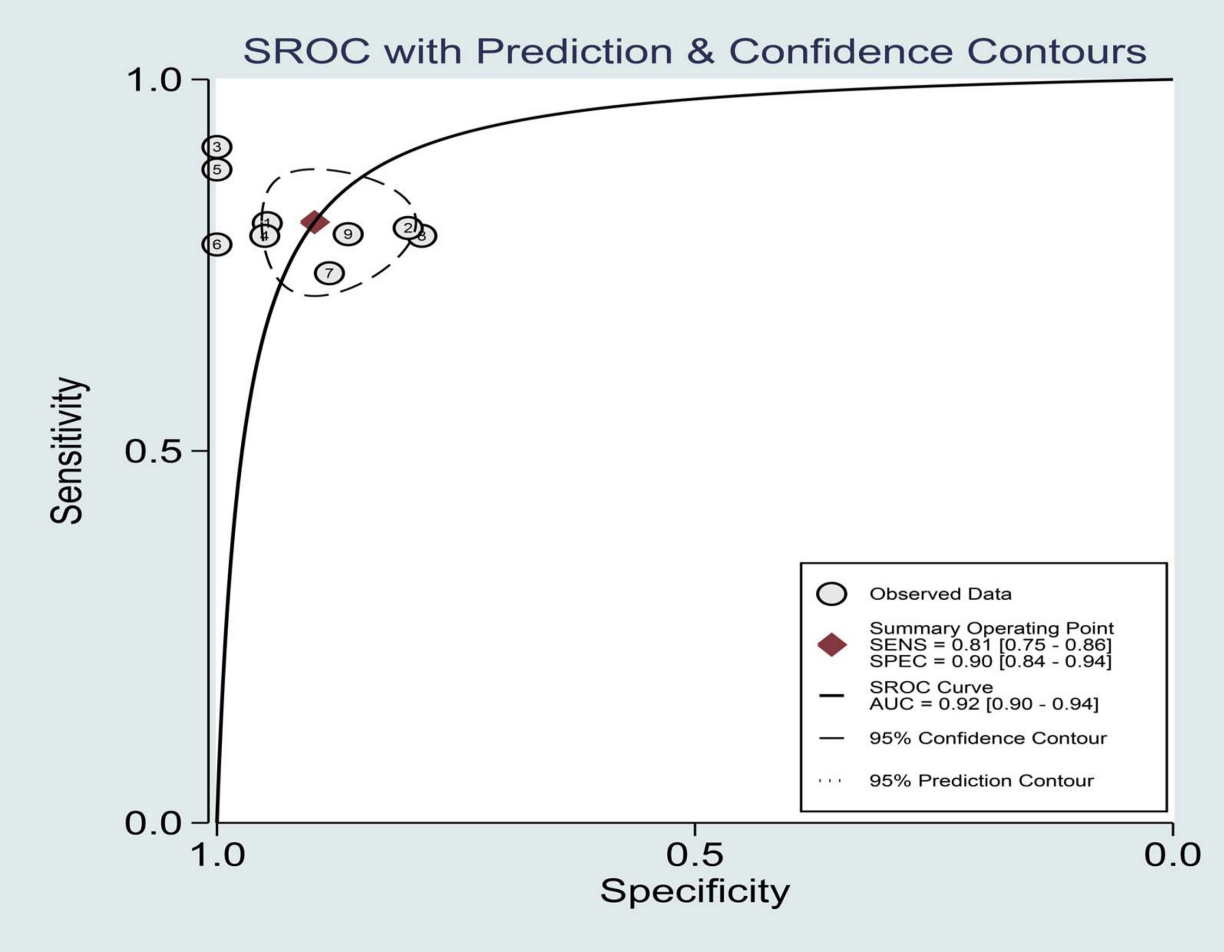
Area under the ROC curve.

**Fig 6:**
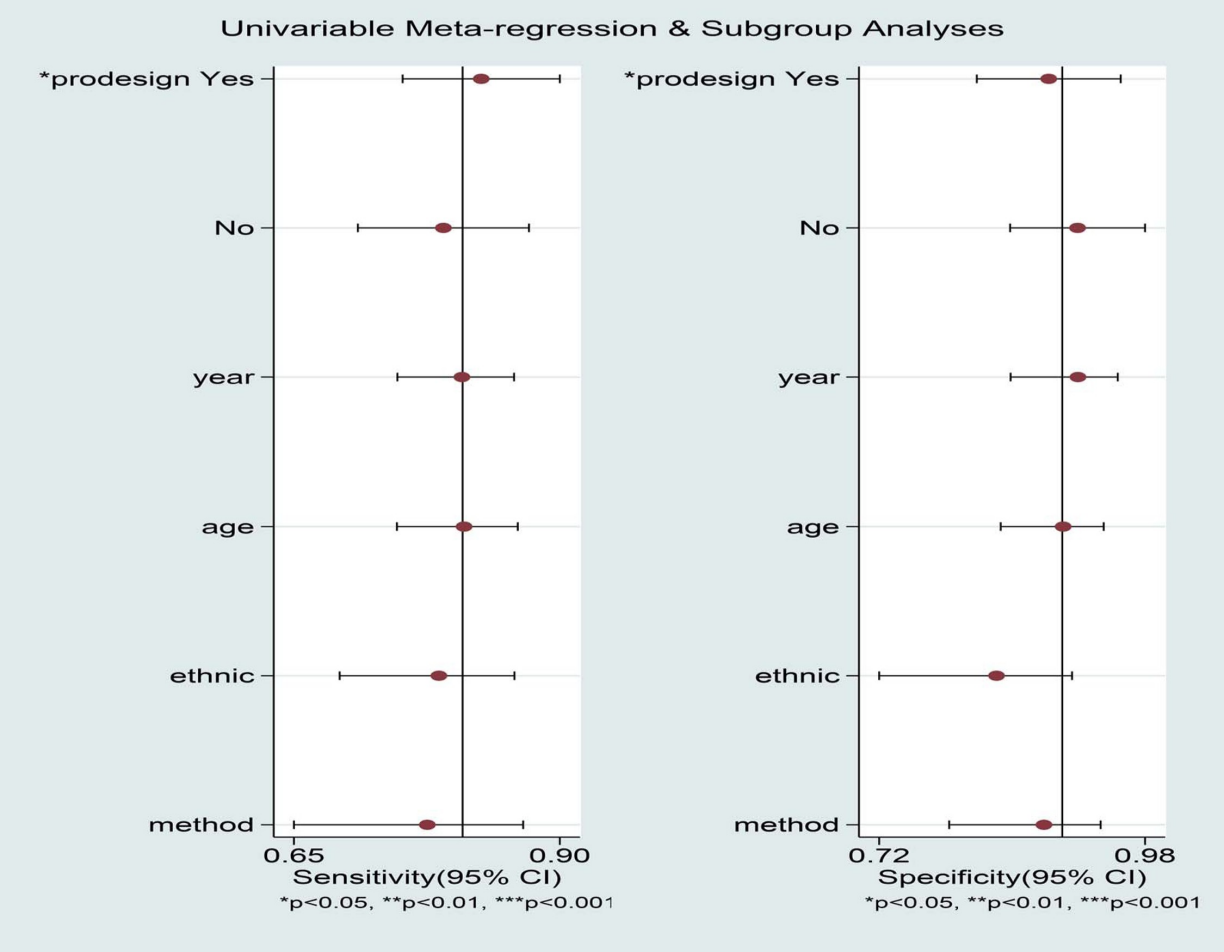
meta-regression analyses for DOR.

**Fig 7:**
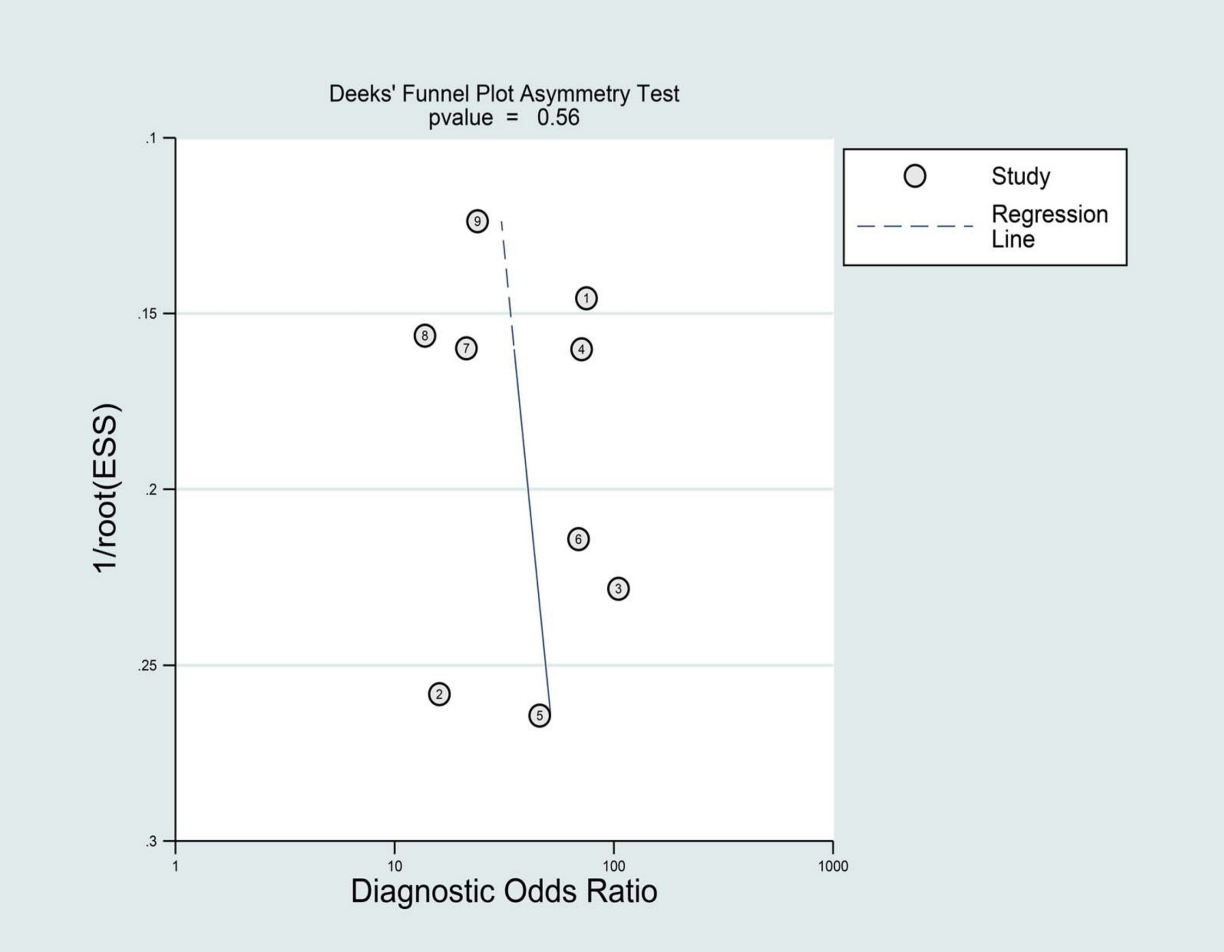
Deeks’ plot for EUS for localization of insulinoma.

The summary results for sensitivity and specificity are presented in Fig 3. The pooled sensitivity was 0.81 (95%CI 0.75–0.86), specificity was 0.90 (95%CI 0.84–0.94), PLR was 7.90 (95%CI 4.90–12.8), and NLR was 0.21(95%CI 0.16–0.29). Further, we noted the pooled DOR of EUS for localization of insulinoma was 37.00 (95%CI:19.55–70.04; Fig 4). Finally, The summary area under the ROC curve was 0.92 (95%CI: 0.90–0.84; Fig 5). Results of the heterogeneity (P = 0.04, I-square: 51.36) was calculated using the forest of DOR, and meta-regression was conducted based on study design, year of publication, enrolled population, ethnicity and diagnostic methods (Fig 6). The results suggested that heterogeneity was mainly derived from the study design. To review the spots in funnel plot was symmetry, and the Deeks’ asymmetry test showed no evidence of publication bias (p = 0.56; Fig 7).

**Table 1:** Characteristics of the studies included in the meta-analysis.

Author	Year	Country	Study design	Gold standard	Apparatus	Method	TP	FP	FN	TN
Rosch [16]	1992	German	Prospective	Pathology	Olympus GF-UM2 GF-UM3	Water-filled balloon method	25	1	6	18
Glover [17]	1992	UK	Retrospective	Pathology	GF-UM2	Water-filled balloon method	12	1	3	4
Pitre[18]	1996	France	Retrospective	Pathology	GF-UM2 GF-UM3	Water-filled balloon method	10	0	1	7
Proye[19]	1998	France	Prospective	Pathology	Olympus	N/A	15	1	4	19
Andenson[20]	2000	USA	Retrospective	Pathology	Olympus UM-20 Pentax FG 32	N/A	29	0	4	3
Ardengh[21]	2004	Brazil	Retrospective	Pathology	FG32-UA	N/A	7	0	2	11
Wang [22]	2006	China	Retrospective	Pathology	Pentax PG-36UX	Water-filled balloon method	17	2	6	15
Sotoudehmandesh[23]	2007	Iran	Prospective	Pathology	FG-UMQ 240	N/A	30	3	8	11
Xun[24]	2009	China	Prospective	Pathology	JF-UM20	Air-bag contact Water-filled balloon method	19	7	5	44

## Discussion

This is the first meta-analysis evaluate the diagnostic value of EUS for localization of insulinoma, and this study included 9 studies involved a total of 350 patients with a broad characteristics. The findings of this meta-analysis found the sensitivity and specificity of EUS for localization of insulinomas were 81% and 90% respectively, this results suggested a acceptable detection rate. Further, the high DOR suggested stronger discrimination ability for localization of insulinomas. In addition, the area under the ROC was 0.92, which indicated a high diagnostic accuracy rate. Therefore, EUS was associated with high diagnostic value for preoperative localization of insulinomas, and combined EUS with digital subtraction angiography and arterial stimulation of venous blood test to reduce the misdiagnosis rate and improve the diagnostic value for preoperative localization of insulinomas.

The pancreatic lesions using EUS with high resolution at close-ranges via transluminal sonography through the duodenum and gastric body, which was associated with detailed observation in target tissue and organs. However, previous study suggested the detected of insulinomas using EUS was determined by the location and size of the lesions. They point out the lesion in pancreatic head if easier observed than those in the tail of the pancreas and outside the pancreas [23]. However, the technical requirement in EUS is difficult due to it difficulty to find tiny lesions, tumors hidden in anatomical locations or other organs. The diagnostic value of EUS might affect in thin patients, female, and more younger patients [25,26].

The strengths of this study were that we followed a standard protocol and used a comprehensive search strategy. Furthermore, bivariate random effects model and hierarchical summary ROC analyses were also calculated. In addition, the large sample size was pooled and the findings of this study are more robust than any individual study. Finally, substantial heterogeneity was explored using meta-regression analyses, and indicated the heterogeneity was observed due to different study design.

The limitations of this study should be mentioned. First, the data abstracted for the details of patients characteristics were not available, which might affect the diagnostic value of EUS. Second, the analysis used summarized data, which restricted us conducting more detailed analysis. Finally, the current study based on published studies, and publication bias is inevitable problem.

The current study was first meta-analysis to determine the diagnostic value of EUS for localization of insulinomas, and suggested EUS was associated with high diagnostic value for localization of insulinomas. These findings needed further large-scale prospective study to verify and evaluated the diagnostic value of EUS in patients with specific characteristics.

## Supporting information

S1 File PRISMA checklist. (DOC)

S2 File Included studies. (ZIP)
