# Supplementary material for: Diagnostic value of endoscopic ultrasound for insulinoma localization: A systematic review and meta-analysis
Source: PLoS One. 2018 Oct 23;13(10):e0206099. doi: 10.1371/journal.pone.0206099 (PMC6198953; doi:10.1371/journal.pone.0206099)
Supplement: S2 File — (ZIP) [file pone.0206099.s002.zip › included studies data availability EUS/Comparison of endoscopic ultrasonography and digital subtracation angiography in localization of insuinlomas.pdf]

论 著

内镜超声与选择性腹腔动脉造影用于胰岛素瘤定位诊断的比较

王国君[1]，窦京涛[2]，王志强[2]，陆菊明[2]

【文章编号】1005－2194（2006）22－1829－02 【中图分类号】R5 【文献标志码】A

【摘 要】 目的 比较内镜超声（EUS）和选择性腹腔动脉造影（DSA）在胰岛素瘤定位诊断中的价值。方法 2000—2005 年解放军总医院收治的 23 例胰岛素瘤患者术前均行腹部超声（US）、螺旋 CT 检查，其中 17 例行 EUS 检查、18 例行 DSA 检查，比较各种方法对胰岛素瘤的定位准确率。结果 各种方法阳性检出率为：US 13.3%、CT 50%、EUS 73.9%、DSA 80%，EUS 阳性率明显高于 US 及 CT（$P<0.01$），与 DSA 相近（$P=0.629$）。结论 EUS 检查对胰岛素瘤的定位准确率接近 DSA，是一种无创、有效、简单、易推广的诊断方法。

【关键词】 内镜超声；胰岛素瘤，定位诊断

Comparison of endoscopic ultrasonography and digital subtracation angiography in localization of insulinomas.

*WangGuojun**, *Dou Jingtao*, *Wang Zhiqiang*, *et al.* * *Departmant of Endocrinology*, *PLA General Hospital*, *Beijing* 100853, China

Abstract **Objective** To compare the value of EUS and DSA in the preopreation localization of insulinomas. **Methods** Twenty－three cases were examined by ultrasonography (US) and spiral computed tomography (CT), of whom 17 cases were examined by endoscopic ultrasonography (EUS), 18 cases were examined by digital subtracation angiography (DSA). The preoperative accurate rates of localizing insulinomas were compared among four methods. **Results** The detective rate of four methods was 13.3% of US, 50.0% of CT, 73.9% of EUS and 80.0% of DSA. The sensitivity of EUS was significantly higher than US and CT ($P<0.001$) and approximate to that of DSA ($P=0.629$). **Conclusion** EUS is a sensitive and easy detective method for insulinoma. It is a valuable site－specific method.

Keywords Endoscopic ultrasonography; Insulinomas, localization diagnosis

胰岛素瘤为胰腺常见内分泌肿瘤，占胰腺内分泌肿瘤的 70%～75%[1－2]。手术切除是最佳治疗方法，术前准确定位是治疗的关键。根据临床表现及生化检查定性诊断相对较容易，但因其体积通常较小，术前定位诊断较困难。常用影像学诊断方法有 US、CT，但阳性率较低，DSA 阳性率较高，但因其有创性、技术条件要求高而应用受限。近年来，随着 EUS 技术的发展，可通过消化道近距离探查整个胰腺，也应用于胰岛素瘤定位诊断。本研究比较了 EUS 和 DSA、US、螺旋 CT 诊断胰岛素瘤的阳性率，评价 ESU 在胰岛素瘤定位诊断中的价值。

1 资料与方法

1.1 研究对象 对 2000—2005 年在解放军总医院内分泌科经手术及术后病理证实为胰岛素瘤的 23 例患者的临床资料进行回顾性分析。其中男 11 例，女 12 例。年龄 13～70 岁，平均 35.4 岁。病程 1 个月至 11 年，平均 3.6 年。所有患者均具有不同程度低血糖发作病史和典型 Whipple 三联症，其中 3 例为胰岛素瘤术后复发。

1.2 方法

1.2.1 定性诊断 23 例患者空腹及低血糖发作时血糖为（2.5±0.2）mmol/L，最低 0.54 mmol/L。血浆胰岛素为（48.7±10.2）mU/L，12 例行 100 g 葡萄糖胰岛素释放试验，同时计算胰岛素血糖比值（INS/BG），均＞0.4，其中 1 例住院期间经饥饿试验诱发出低血糖。

1.2.2 定位诊断 23 例患者均进行 US、螺旋 CT 检查，17 例行 EUS 检查。内镜超声诊断装置是在内镜前端安装一个高频超声波探头，采用水囊与水充盈方法结合，经胃十二指肠扫描胰腺，扫描方式为扇形线阵型扫描，超声诊断仪为日本 Pentax FG－36 UX。18 例进行 DSA 检查，采用改良 Seldinger 技术穿刺股动脉，插入 4F 动脉导管，至

作者单位：1. 内蒙古包头市医学院第二附属医院内分泌科，内蒙古 包头 014030；2. 解放军总医院内分泌科，北京 100853

通讯作者：窦京涛，E－mail：jingtaodou@sohu.com

万方数据

腹腔干动脉、肠系膜上动脉注入优维显 370 造影剂25 mL，用东芝 DFD－O3A 及 Philips H5000 型数字减影血管造影机显影。所有患者均接受外科剖腹探查手术及组织病理学检查。

1.3　**统计学处理**　各组检出率比较采用 χ^2 检验，$P < 0.05$ 为差异有统计学意义，采用 SPSS 软件分析数据。

2　结果

2.1　**术后病理**　22 例为良性胰岛细胞瘤，1 例为胰岛细胞增生。共检出病灶 30 个，其中 3 例为多发胰岛细胞瘤，病灶分别为 6 个、3 个、2 个；病灶部位胰头 12 个、胰体 9 个、胰尾 9 个；病灶大小 2.0～2.5 cm 者 5 个，1.0～1.8 cm 者 16 个、0.3～0.7 cm 者 9 个。

2.2　**病灶检出阳性率**　行 EUS 检查者 17 例，阳性率 73.9%（17/23），其中 1 例病理证实为增生误判为占位，1 例副脾误判为胰尾部占位，同时检出了 DSA 漏检的病灶 3 个；行 DSA 检查者 18 例，阳性率 80%（20/25）；US 阳性率 13.3%（4/30）；螺旋 CT 阳性率 50%（15/30）。EUS 明显优于 US 及 CT（$P < 0.01$），与 DSA 相近（$P = 0.629$）。

2.3　**检出病灶的大小、部位**　本组行 EUS 检查者 17 例，共检出病灶 17 个，直径 1.01～1.18 cm，最小 0.5 cm，检出病灶符合率：胰腺头部病灶 87.5%（7/8）、体部 100%（7/7）、尾部 37.5%（3/8）。漏检的 6 个病灶直径均在 0.3～0.7 cm 之间。假阳性情况：除 1 例增生误判为占位、1 例副脾误判为胰尾部占位，其余均与病理结果一致。18 例行 DSA，检出病灶 20 个，直径 0.89～1.18 cm，最小 0.3 cm。检出病灶符合率：胰腺头部病灶 90.0%（9/10）、体部 60.0（3/5）、尾部 80.0（8/10），病变性质均与病理结果一致，无假阳性出现。

3　讨论

胰岛素瘤分布均匀，胰头、体、尾各占 1/3 左右，直径多在 1.0～2.5 cm，80% 小于 2.0 cm[3]。胰尾部周围脏器结构较多，易相互影响，给肿瘤定位造成困难。胰岛素瘤多为单发，少数多发，大部分为富血管性，极少数为乏血管性或囊性。DSA 检查就是利用肿瘤血供丰富的特点，表现为动脉期肿瘤区血管增多、扭曲及实质期的肿瘤染色。在过去的 20 年中 DSA 一直被认为是胰岛素瘤定位诊断的“金标准”，文献报道阳性率达 67%～87%[3]。本组资料显示，18 例行 DSA 检查与手术及病理符合率为 80%，与文献报道一致。其缺点：（1）假阳性率较高，如副脾、肿大的淋巴结、不透光的肠袢，皆可误认为肿瘤而误导手术。但本研究未出现假阳性。（2）对乏血管性胰岛素瘤检出率低。（3）是有创检查，对技术条件要求高，有一定并发症存在[3]。

内镜超声是使用高频探头从胃或十二指肠内近距离探测胰腺病灶，凭借其高分辨率，更接近靶器官的优点，可对胰腺进行更精确、细致的观察。文献报道内镜超声下胰岛素瘤表现为边缘完整、轮廓清晰、内部呈等回声、低回声的类圆形团块影，诊断准确率可达 77%～96.9%，有较高的定位价值[4]。本组资料显示 EUS 阳性率 73.9%（17/23），略低于文献报道，分析原因：（1）术后胰腺解剖结构不清晰 本组 17 例接受 EUS 检查者，其中 2 例为胰岛素瘤术后复发，胰腺正常结构被破坏。（2）病变体积小 共 23 个病灶，其中 9 个直径为 0.3～0.7 cm，占 39.1%。（3）病灶融合 1 例为多发胰岛素瘤，6 个微小病灶融合在一起，误判为 2 个病灶。以上因素影响了整体阳性检出率。故我们总结 EUS 检查的特点：（1）病灶阳性检出率较传统影像学检查高，与 DSA 相近。（2）是一种无创检查，病人痛苦少、并发症少，技术易于推广。（3）可作为胰岛素瘤术前定位诊断常规检查。EUS 是根据超声图像的改变为依据，有赖于操作医师的经验及技巧，尚存在以下缺点：（1）EUS 对极微小（＜1cm）病灶检出率低。（2）胰尾部病灶检出率相对低，与胰尾部距离探头远及易与脾脏、副脾影像重叠因素有关。（3）对单发病灶检出率高，对多发微小病灶检出率低。本研究假阳性率为 8.7%（2/23）。

本组资料显示 EUS 阳性率 73.9%（17/23），DSA 阳性率 80%（20/25）；US 阳性率 13.3%（4/30）；螺旋 CT 阳性率 50%（15/30），EUS 明显优于 US 及 CT（$P < 0.01$），与 DSA 相近（$P = 0.629$）。EUS 阳性率接近 DSA，但由于 DSA 为有创性检查，且操作复杂，有一定的并发症，只能在少数三级甲等医院进行，尚不能广泛应用，而 EUS 检查对胰岛素瘤术前定位诊断优于 CT、US 等传统影像学方法，是临床有价值、简单、易推广的方法，可作为定位检查的第一选择。有作者报道 EUS 联合螺旋 CT 阳性率可达 100%，故术前定位诊断可参考 2 种以上影像学检查方法，对指导手术治疗更有意义[5]。

参考文献

[1] Dolan JP，Norton JA. Occult insulinoma［J］. Br J Surg，2000，87（4）：385－387.

[2] 胡振序，贺兵. 胰岛素瘤伴神经精神症状 15 例临床分析［J］. 中国实用内科杂志，1988，12：21－23.

[3] 杨志英，刘展，赵平，等. 1078 例胰岛素瘤的定位诊断［J］. 中华外科杂志，2000，38（1）：13－15.

[4] Ardengh JC，Rosenbaum P，Ganc J，et al. Role of EUS in the preoperative localization of insulinoma compared with spiral CT［J］. Gastrointest Endosc，2000，51：522－525.

[5] McLean AM，Fairclough PD. Endoscopic ultrasound in the localization of pancreatic islet cell tumours［J］. Best Pract Res Clin Endocrinol Metab，2005，19（2）：177－193.

2006－07－06 收稿　2006－08－14 修回　本文编辑：宇广华

万方数据
