# Supplementary material for: Diagnostic value of endoscopic ultrasound for insulinoma localization: A systematic review and meta-analysis
Source: PLoS One. 2018 Oct 23;13(10):e0206099. doi: 10.1371/journal.pone.0206099 (PMC6198953; doi:10.1371/journal.pone.0206099)
Supplement: S2 File — (ZIP) [file pone.0206099.s002.zip › included studies data availability EUS/Endoscopic ultrasonography (EUS) in the localization.pdf]

Endocr (2007) 31:238–241
DOI 10.1007/s12020-007-0045-4

ORIGINAL PAPER

Endoscopic ultrasonography (EUS) in the localization of insulinoma

Rasoul Sotoudehmanesh · Anooshirvan Hedayat · Nahid Shirazian · Shadi Shahraeeni · Sanaz Ainechi · Fatemeh Zeinali · Shadi Kolahdoozan

Received: 30 April 2007 / Accepted: 20 July 2007 / Published online: 25 August 2007
© Humana Press Inc. 2007

Abstract
Objective Endoscopic ultrasonography has been accepted as a sensitive modality for preoperative tumor localization in pancreas. We have aimed to determine the performance characteristics of endoscopic ultrasonography in pancreatic insulinoma localization and evaluation of relationship between the tumor size and serum-c peptide level, lowest glucose level and insulin level.
Methods Patients suspicious to insulinoma according to clinical and laboratory findings were included. Endoscopic ultrasonography was performed and if a tumor was identified, the patient was referred for surgery.
Results A total of 52 patients (24 male and 28 female) with mean age of 42.4 years underwent EUS and 43 patients underwent surgery. In one patient, a tumor was identified both by transabdominal ultrasonography and abdominal CT scan. The overall sensitivity and accuracy of endoscopic ultrasonography for detection of insulinoma was 89.5% and 83.7% respectively. The sensitivity of endoscopic ultrasonography for detection of lesions in pancreatic head, body and tail was 92.6%, 78.9%, and 40.0%, respectively. There was no relationship between c-peptide, lowest blood glucose, insulin blood levels and tumor size in surgery.
Conclusion EUS is an accurate method for detection of insulinoma. The accuracy depends on the location of the tumor and is greatest for tumors in the pancreatic head.

Keywords Insulinoma · Endosonography · Sensitivity · Surgery

R. Sotoudehmanesh (✉) · S. Shahraeeni · S. Ainechi · F. Zeinali · S. Kolahdoozan
Digestive Disease Research Center, Medical Sciences/University of Tehran, Shariati Hospital, North Kargar Ave, 14117-13135 Tehran, Iran
e-mail: setoodeh@ams.ac.ir

A. Hedayat
Department of Surgery, Medical Sciences/University of Tehran, Shariati Hospital, North Kargar Ave, 14117-13135 Tehran, Iran

N. Shirazian
Islamic Azad University, Tehran School of Medical Sciences, Tehran, Iran

Introduction

Insulinoma is the most common endocrine tumor of the pancreas with a reported annual incidence ranging between 1 and 10 cases per million in the general population [1, 2]. The clinical presentation includes fasting symptoms of hypoglycemia, low blood glucose level, and symptomatic relief with glucose administration; a triad known as Whipple's triad [3]. The tumor is often small; 90% are less than 2 cm in diameter. They are found evenly distributed over the whole pancreas. As a general rule, patients with insulinoma can be cured by surgical resection of the tumor. In experienced centers, most pancreatic endocrine tumors, including insulinoma, could be found during surgery by careful palpation of the pancreas. Intraoperative ultrasonography is also helpful, but is technically demanding. Traditional imaging techniques, such as computed tomography (CT), transabdominal ultrasonography (US) and magnetic resonance imaging (MRI) are not sensitive enough to reliably identify these small tumors. The sensitivity of US and CT has been 15.0% and 64.0%, respectively [4]. Angiography and transhepatic venography have higher sensitivities, but are invasive, expensive, difficult to perform, and have high risk of complications [5–7]. The high spatial resolution of EUS allows the

detection of small lesions and their precise anatomical localization. The sensitivity of EUS for detection of tumor location has been reported to be 65.0–94.0% with a mean sensitivity of 84.0–89.0%. EUS is a minimally invasive, safe and accurate method for localization and imaging of pancreatic tumors, especially insulinoma.

We have assessed the accuracy of EUS in detection of insulinoma in different parts of the pancreas, and the relationship between tumor size and serum C-peptide, blood glucose, and insulin levels.

Material and methods

From October 2002 to November 2006, all patients suspicious of harboring an insulinoma referred for endosonography were enrolled. Diagnosis of insulinoma before EUS was based on clinical features and biochemical tests including hypoglycemia, high insulin and C-peptide levels, and negative screen of urine and blood for anti-diabetic agents. All patients underwent abdominal US and CT before EUS. All the EUS procedures were performed by a single experienced endosonologist using a radial echoendoscope (GF-UMQ 240 Olympus Optical Co Ltd, Tokyo, Japan) with a frequency of 7.5 MHz. EUS examination was performed with the patient in the left lateral decubitus position under conscious sedation with midazolam with or without meperidin The uncinate process and head of the pancreas were scanned from the duodenum and the body and tail of pancreas from the stomach. Patients with pancreatic mass detected in EUS and patients with normal EUS but with typical clinical and biochemical evidence of insulinoma were referred for surgery. In this study surgery was used as gold standard for localization of insulinoma tumors.

The study was approved by the institutional review board and ethics committee of the Digestive Diseases Research Center of Tehran University of Medical Sciences. Informed consent was obtained according to the guidelines of the institute.

Results

A total of 52 (24 men and 28 women) with median age of 42.4 years (16–74 years) patients underwent EUS and 43 were referred for surgery. The average duration of symptoms before EUS was 3.5 years (3 months to 10 years). None had familial disease or multiple endocrine neoplasias (MEN). An aliquot of 40 cases (90.9%) had Whipple's triad (a low serum glucose concentration, hypoglycemic symptoms and relief by raising serum glucose). A total of 29 patients (65.9%) had gained and 7 patients (15.9%) lost weight. In two patients, tumor was not found by EUS or surgery. Characteristics of patients and their tumors are given in Table 1.

Table 1 Characteristics of patients and their insulinoma tumors

Characteristics	Mean (range)
Age	42.4 (16–74)
Sex	
Male; n (%)	24 (46.1)
Female; n (%)	28 (53.8)
Duration of symptoms (years)	3.5 (0.25–10)
Lowest Blood Glucose level (mg/dl)	26.2 (12–45)
Tumor size in surgery (mm)	17.3 (8–50)
C-peptide level (ng/dl)	5.3 (1.1–25.4)
Insulin level (mIu/ml)	26.3 (0.5–114)

Nine patients did not agree to surgery despite EUS localization of their tumor. They were treated medically by diazoxide.

In patients who underwent surgery, the mean lowest blood glucose level was 26.2 mg/dl (12–45 mg/dl), C-peptide level: 5.3 ng/dl (1.1–25.4 ng/dl) and insulin level: 26.3 mIu/ml (0.5–114 mIu/ml). Only in one patient abdominal sonography and CTscan successfully localized the tumor. The sensitivity of CT and TUS in detecting pancreatic insulinomas was therefore 2.0% (1/39).

A total of 37 solitary tumors were localized by EUS; 17 in the pancreatic head, 16 in the body and 4 in the tail. The average largest tumor diameter measured by EUS was 14.4 mm (5–30 mm). A total of 38 solitary tumors were detected during surgery; 14 in the head, 19 in the body and 5 in the tail. The average tumor diameter in surgery report was 17.3 mm (8–50 mm). In three patients, EUS reported a tumor in the pancreas but during surgery they turned out to be lymph nodes. In two patients the lymph nodes were in the head of the pancreas and in one patient in the tail. In the latter case, the insulinoma was located in the head of pancreas found during surgery. In two other cases no tumor was found during surgery. The overall sensitivity of EUS in the detection of pancreatic insulinoma was 89.5% with an accuracy of 83.7% and a positive predictive value of 91.2%. The sensitivity of EUS for detecting insulinoma in the head of pancreas was 92.6% (13 out of 14) and 78.9% for body tumors (15 out of 19) and 40.0% for tumors located in the tail (2 out of 5).

Table 2 shows a summary of localization of Insulinoma tumor by EUS and surgery.

The average diameter of tumors which were not detected by EUS was 14 mm (8–20 mm). The mean diameter of tumors detected or not detected by EUS were not significantly different ($P = 0.4$).

Table 2 Localization of Insulinoma tumor by EUS and surgery

		Surgery			
		Head	Body	Tail	No tumor
EUS	Head	13	0	2	2
	Body	0	15	0	1
	Tail	1	1	2	0
	No tumor	0	3	1	2

There was no relationship between c-peptide; lowest blood glucose and insulin blood levels with tumor size in surgery (Plots 1, 2).

Figure 1 shows an insulinoma found in the head of pancreas by EUS.

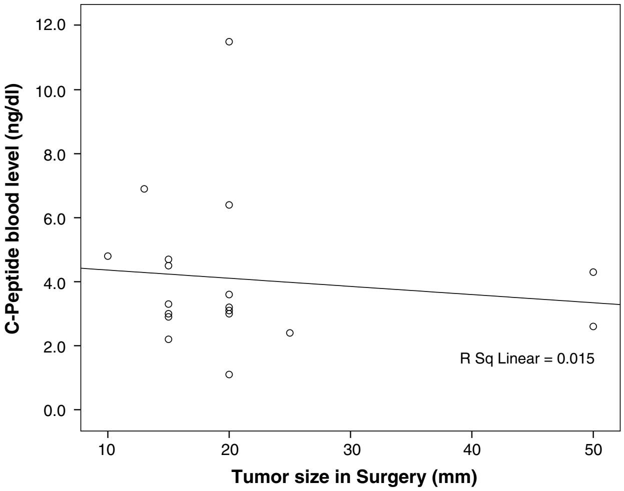

Plot 1 The relationship between C-peptide level and tumor size in surgery

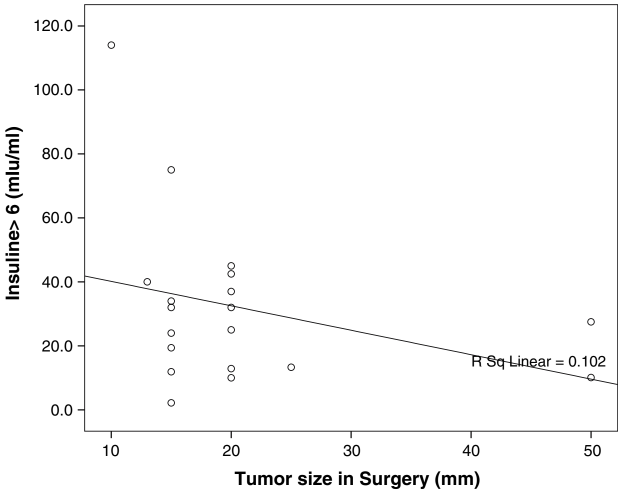

Plot 2 The relationship between insulin level and tumor size in surgery

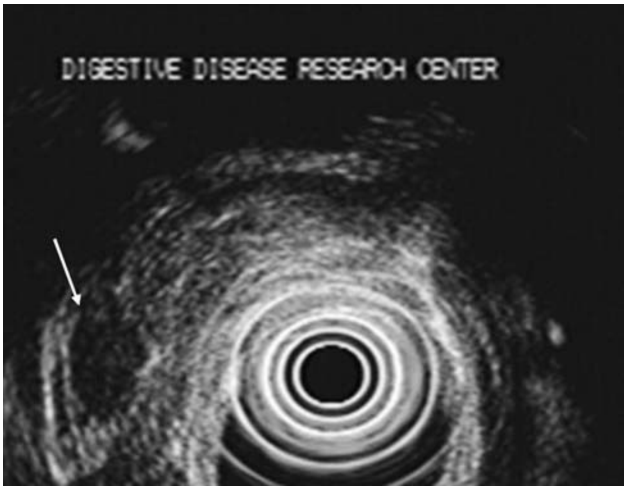

Fig. 1 Insulinoma located within the head of pancreas (arrow)

Discussion

This study revealed the high sensitivity and accuracy of EUS for detection of insulinoma. This sensitivity depends on the location of the tumor; highest in the head and lowest in the tail of the pancreas. Preoperative localization of insulinoma is important in order to reduce the extent of mobilization of the pancreas during surgery, the size of resection, the duration and complexity of the operation and the overall surgical morbidity [4]. When insulinoma tumors are located preoperatively they can frequently be removed laparoscopically.

Non invasive imaging techniques such as CT scan, US, and MRI have not been sufficiently sensitive for localizing insulinomas [4, 8]. Several other studies have also demonstrated EUS to be the most sensitive method for preoperative localization of insulinoma. In addition, the procedure is generally well tolerated by patients. In a study performed on 30 patients by Fendrich et al, the overall sensitivity reported for EUS, was 86.6%, compared to 33.0% for CT and 40.0% for MRI [8]. In another study, sensitivity of the localization procedure was as follows: US: 9.6%, CT: 39.0%, EUS: 92.3% [9]. In our study, spiral CT and US only detected one patient with insulinoma. A possible explanation is that the CT scans performed in our study were spiral or conventional CTs (not multi-detector CT). In addition to the machine used, the technique and meticulousness of the performers are critical issues. The likelihood of detection of pancreatic endocrine tumors by CT depends upon the tumor size and location. Although tumors as small as 0.7 cm in diameter have been located, those less than 2 cm cannot be found consistently [4]. The sensitivity of CT for detecting primary gastrinomas or insulinomas has ranged from 31% to 59% [10–12]. The sensitivity for liver metastases is 42–72% [10, 11].

Sensitivity for detection of primary tumors is increased with spiral CT (82–92% in two reports) [13, 14]. However more studies are required for comparison between multi-detector CT and EUS in localization of insulinomas.

The overall sensitivity of EUS in the detection of pancreatic insulinoma was 89.5% that is comparable with other studies. In our study the sensitivity of EUS for localization of insulinomas in the head, body and tail of pancreas was 92.6%, 78.9%, and 40.0%, respectively. The differences in sensitivities are independent of the tumor diameter as measured by EUS. In a study by Schumacher et al. [15] the overall sensitivity of EUS in the detection of pancreatic insulinoma was 57.0% (8 out of 14 tumors). The sensitivity for detecting insulinoma in the head of the pancreas was 83.0% and for tumors in the tail of the pancreas 37.0%. In another study [6] of six tumors that were not detected by EUS, five insulinomas were located in the body or tail of the pancreas. These studies suggest that the sensitivity of EUS in detecting pancreatic insulinomas depends on the location of the tumor. According to these studies and ours, EUS sensitivity is higher in detecting tumors in the head compared with the tail of pancreas, most likely because of differences in the ability of EUS in visualizing different parts of the pancreas.

The average tumor diameter, as measured by EUS, was 14.4 mm and the average tumor diameter measured during surgery was 17.3 mm. Other studies have also reported that the tumor size as measured by EUS is from 10% to 20% less than the size seen during surgery. [15, 16] The reason for this discrepancy remains unclear, but it may be due to the two-dimensional nature of EUS measurements. The spheroid configuration of most insulinomas that cannot be detected by EUS, implying that the value of EUS in detecting the correct tumor size may be limited.

Insulinomas in the tail of pancreas can be detected by intraoperative ultrasonography as well. Sensitivity of combined intraoperative ultrasonography and palpation has been reported to be, as high as 100%. However, this method prolongs surgery and carries the risk of rupture of the splenic vessels due to mobilization of the pancreas [4, 10].

A limitation of our study is the use of surgery as the gold standard for diagnosis of insulinoma and the absence of intraoperative ultrasonography. Currently intraoperative ultrasonography of the pancreas in conjunction with palpation has been accepted as the gold standard for the localization of insulinoma, despite the difficulties and complications.

In three of our patients, EUS reported insulinoma in the pancreas but in surgery they turned out to be lymph nodes. Differentiation of peripancreatic lymph nodes from intrapancreatic tumors may occasionally be difficult especially in small tumors [15, 17].

In conclusion, EUS has a high sensitivity for the identification and localization of pancreatic insulinoma, which partly depends on the location of tumor.

Acknowledgment We thank Dr. Shahin Merat, Dr. Alireza Sajadi and Dr. Siavosh Nasseri-Moghaddam for their help to edit this article. **Funding:** The authors declare that there is no conflict of interest that would prejudice the impartiality of this scientific work.

References

1. F.J. Service, M.M. McMahon, P.C. O'Brien, D.J. Ballard, Mayo Clin. Proc. **66**, 711–719 (1991)
2. G. Tamburrano, A. Paoloni, D. Pietrobono, E. D'Amico, C. Durante, R. Baldelli, Ital. J. Gastroenterol. Hepatol. **31**(Suppl 2), S104–7 (1999), S104–S107
3. J.A. van Heerden, A.J. Edis, F.J. Service, Ann. Surg. **189**, 677–682 (1979)
4. C.M. King, R.H. Reznek, J.E. Dacie, J.A. Wass, Clin. Radiol. **49**, 295–303 (1994)
5. J. Pitre, O. Soubrane, L. Palazzo, Y. Chapuis, Pancreas **13**, 55–60 (1996)
6. T. Rosch, C.J. Lightdale, J.F. Botet, G.A. Boyce, M.V. Sivak Jr., K. Yasuda, N. Heyder, L. Palazzo, H. Dancygier, V. Schusdziarra, N. Engl. J. Med. **326**, 1721–1726 (1992)
7. N.M. Kuzin, A.V. Egorov, S.A. Kondrashin, A.N. Lotov, N.S. Kuznetzov, J.B. Majorova, World J. Surg. **22**, 593–597 (1998)
8. V. Fendrich, D.K. Bartsch, P. Langer, A. Zielke, M. Rothmund, Dtsch. Med. Wochenschr. **129**, 941–946 (2004)
9. L.B. Nesje, J.E. Varhaug, E.S. Husebye, S. Odegaard, Scand. J. Gastroenterol. **37**, 732–737 (2002)
10. A.K. Galiber, C.C. Reading, J.W. Charboneau, P.F. Sheedy, E.M. James, B. Gorman, C.S. Grant, J.A. van Heerden, R.L. Telander, Radiology **166**, 405–408 (1988)
11. S.A. Wank, J.L. Doppman, D.L. Miller, M.J. Collen, P.N. Maton, R. Vinayek, J.I. Slaff, J.A. Norton, J.D. Gardner, R.T. Jensen, Gastroenterology **92**, 905–912 (1987)
12. F. Gibril, J.C. Reynolds, J.L. Doppman, C.C. Chen, D.J. Venzon, B. Termanini, H.C. Weber, C.A. Stewart, R.T. Jensen, Ann. Intern. Med. **125**, 26–34 (1996)
13. L. Van Hoe, S. Gryspeerdt, G. Marchal, A.L. Baert, L. Mertens, AJR Am. J. Roentgenol. **165**, 1437–1439 (1995)
14. P. Legmann, O. Vignaux, B. Dousset, A.J. Baraza, L. Palazzo, I. Dumontier, J. Coste, A. Louvel, G. Roseau, D. Couturier, A. Bonnin, AJR Am. J. Roentgenol. **170**, 1315–1322 (1998)
15. B. Schumacher, H.J. Lubke, T. Frieling, G. Strohmeyer, A.A. Starke, Endoscopy **28**, 273–276 (1996)
16. J.C. Ardengh, P. Rosenbaum, A.J. Ganc, A. Goldenberg, E.J. Lobo, C.A. Malheiros, F. Rahal, A.P. Ferrari, Gastrointest. Endosc. **51**, 552–555 (2000)
17. C.J. Lightdale, J.F. Botet, J.M. Woodruff, M.F. Brennan, Cancer **68**, 1815–1820 (1991)
