# Supplementary material for: Diagnostic value of endoscopic ultrasound for insulinoma localization: A systematic review and meta-analysis
Source: PLoS One. 2018 Oct 23;13(10):e0206099. doi: 10.1371/journal.pone.0206099 (PMC6198953; doi:10.1371/journal.pone.0206099)
Supplement: S2 File — (ZIP) [file pone.0206099.s002.zip › included studies data availability EUS/Endoscopic Ultrasonography for the Preoperative.pdf]

Pancreas
Vol. 13, No. 1, pp. 55–60
© 1996 Lippincott–Raven Publishers, Philadelphia

Endoscopic Ultrasonography for the Preoperative Localization of Insulinomas

J. Pitre, O. Soubrane, L. Palazzo, and Y. Chapuis

Clinique Chirurgicale, Hôpital Cochin, Paris, France

Summary: Preoperative radiological localization of insulinomas often fails because of the small size of the tumors. We studied retrospectively the value of different procedures in preoperative localization of insulinomas in 18 patients. Radiological assessment included transabdominal ultrasonography, computed tomography, angiography, magnetic resonance imaging, transhepatic venous sampling, and endoscopic ultrasonography (EUS) for the last 11 patients. During surgery, the association of palpation and intraoperative ultrasonography localized 16 solitary tumors and two multiple tumors (mean size, 1.8 ± 1.1 cm). Three insulinomas were found to be malignant. Conventional preoperative methods correctly localized the tumor in seven of 18 cases (38%), whereas the sensitivity of EUS was 10 of 11 cases (90%). Surgical procedures involved eight enucleations, nine distal pancreatectomies, and one total pancreatectomy. Because of its high sensitivity and safety, EUS was found to be the best method for preoperative localization of insulinomas, and we recommend that EUS replace conventional methods for the majority of cases. **Key Words:** Pancreas—Insulinoma—Endoscopic ultrasonography.

Insulinoma is a rare disease but the most frequent cause of pancreatic endocrine tumors (1). Symptoms of hypoglycemia are usually suggestive allowing the diagnosis of organic hyperinsulinemia to be made. These tumors are generally benign, solitary, intrapancreatic, and small, measuring <2 cm in 66% of cases and <1 cm in 24% of cases (2). Surgical resection is the treatment of choice. However, previous reports have shown that, in the absence of localization procedure, insulinoma may not be localized by palpation alone in up to 20% of laparotomies (2–4). To facilitate surgery, preoperative imaging procedures such as transabdominal ultrasonography (US), computed tomography (CT), angiography, and transhepatic venous sampling (THVS) have been used to localize the primary tumor. More recently, promising results were reported with selective intraarterial injection of calcium (5) and methylene blue (6). Noninvasive methods (US and CT) have a low sensitivity (0–73%) (2,7–15), whereas invasive methods (angiography, THVS) are more accurate (52–100%) but are expensive and time-consuming and require an experienced radiologist (2,9,10,12–18). Since the introduction of intraoperative ultrasonography (IOUS) (19), nearly 90% of insulinomas can be localized at surgery in experienced centers, but surgery is still facilitated by preoperative localization (9–13,17,20–22). Endoscopic ultrasonography (EUS) achieves as complete an analysis of pancreas parenchyma as IOUS, and recent studies have advocated the use of EUS in pancreatic tumor diagnosis (23–24). The aim of this study was to evaluate the efficiency of preoperative EUS in localizing pancreatic insulinomas.

Manuscript received May 26, 1995; revised manuscript accepted August 21, 1995.
Address correspondence and reprint requests to Pr. Y. Chapuis, Clinique Chirurgicale, Hôpital Cochin, 27 Rue du Faubourg Saint-Jacques, 75014 Paris, France.
Presented in part at the First European Congress of the World Association of Hepato-Pancreato-Biliary Surgery, Paris, France, 8–11 June 1993.

PATIENTS AND METHODS

Patients

Between October 1983 and May 1993, 18 consecutive patients with a diagnosis of insulinoma were operated on. One patient had previously undergone

a blind distal splenopancreatectomy and no tumor was found. This patient was referred to our center 3 months later and reoperated on for a persistent insulinoma.

Diagnosis

An inappropriately elevated immunoreactive insulin (IRI; normal range, 3–25 μU/ml) for the level of blood glucose confirmed the diagnosis. Other causes of fasting hypoglycemia (such as antiinsulin antibodies and self-administration of sulfonylureas or insulin) were cautiously discarded (25). In all but two patients, diagnosis proceeded with a supervised fast and the mean serum level of glucose was 2.7 ± 1.0 m*M*/L while the mean serum level of insulin was 28 ± 20 μU/ml. Fifteen of 16 supervised fasts were interrupted before 48 h because of the occurrence of a malaise associated with glycemia lower than 2.2 m*M*/L. The median value of the Turner ratio (26) was 479. The median plasma level of C-peptide, measured in six patients, was 3.7 ng/ml (normal range, 0.5–3).

Preoperative localization

From 1983 to 1989, each patient underwent some form of preoperative localization including transabdominal US, CT, and, in some cases, magnetic resonance imaging (MRI), selective angiography, and THVS of the portal vein and its tributaries for insulin level. Since 1990, EUS has been used routinely for each suspected case of insulinoma.

EUS used echoendoscopes (Olympus GF UM 2 and GF UM 3, Japan) with 7.5- and 12.5-MHz scanners and was performed under mild analgesia. The endoscope was introduced in the descending part of the duodenum and the different parts of the pancreas were explored by slowly withdrawing the transducer. The head of the pancreas was scanned from the duodenum and the rest of the pancreas from the stomach. A water-filled balloon surrounding the transducer formed a fluid interface between the transducer and the gastrointestinal wall. In some instances, it was necessary to fill the stomach with water (600 ml). The accuracy of EUS was evaluated by the ability to localize and measure the tumor, as compared with IOUS, surgical exploration, and pathological findings.

Peroperative localization

All operations were performed in a standardized fashion. The abdominal cavity was entered through an upper transverse incision. The liver was examined for the presence of metastases and biopsy samples were taken from any enlarged lymph nodes near the pancreas or along the portal triad. The pancreas was mobilized by an extended Kocher maneuver. The uncinate process was separated from the root of the mesentery. The body and the tail of the pancreas were examined by dividing the gastrocolic omentum and entering the lesser sac. To analyze the whole pancreatic gland, complete mobilization of the pancreatic tail and spleen was achieved. However, this maneuver was not performed when the tumor was localized by EUS prior surgery. The pancreas was then inspected and palpated. After palpation, real-time high-resolution IOUS with 7.5-MHz and, more recently, 10-MHz scanners (CGR, Thomson, France) was performed. Methodology of IOUS of the pancreas has been described elsewhere (22).

Pathology

Pathological examination investigated the tumor, the pancreatic tissue surrounding the tumor and peripancreatic or portal lymph nodes, or liver metastases. Conventional colorations and histochemical reactions were performed. Immunohistochemical stainings scanned the endocrine feature of the tumor.

Statistical methods

Results are expressed as means (±SD) or as medians. Statistical comparison of proportions was accomplished by Fischer's exact test. Differences were considered to be statistically significant at $p < 0.05$.

RESULTS

Preoperative localization

Transabdominal US correctly localized the tumor in three of 17 cases, compared with five of 17 cases for CT scan. Angiography localized the tumor in two of seven cases, and MRI in two of six cases. THVS, used in two cases (one with calcium injection), failed to localize the insulinoma. There were three false-positive CT scans and one false-positive angiogram. When operative findings were compared to the results of preoperative localization studies, tumors were correctly localized by these conventional imaging methods in seven of 18 patients (38%).

EUS, performed in the last 11 patients in this survey, localized the tumor in 10 cases. The sensitivity was significantly higher than for any other imaging method ($p < 0.05$). For eight of 11 patients, EUS was the only method that visualized the tumor. No false-positives were observed. Furthermore, the procedure was always well tolerated by the patients. Benign tumors were usually hypoechoic and homogeneous with a smooth margin ($n = 7$) (Fig. 1) and rarely isoechoic ($n = 1$). Malignant tumors ($n = 3$) were hypoechoic with irregular margins, multiple peripancreatic enlarged lymph nodes, and partial splenic vein invasion with collateral circulation ($n = 2$). One corporeal tumor, measuring 0.8 cm, was not detected by EUS, probably because its echo pattern was closely similar to that of the rest of the pancreas. The size of the smallest tumor visualized by EUS was 0.8 cm. The patient with the MEN 1 had seven endocrine pancreatic tumors, of which two were insulinomas. In this case, all the tumors were visualized by EUS. In a recent case of previous blind distal pancreatectomy, a first attempt at EUS did not demonstrate insulinoma, but a second investigation by an experienced operator detected an isoechoic intracephalic tumor of 1.2 cm.

Peroperative exploration

At surgery, tumors were clearly palpable in 13 of 18 cases (72%). For two patients, results of cautious palpation were doubtful. Results of IOUS for ultrasonographic features were consistent with those of EUS.

Sixteen patients had a solitary insulinoma, while one patient had two tumors and another had seven pancreatic endocrine tumors. The mean size of the main tumor was 1.8 ± 1.1 cm (range, 0.8–5 cm). Half of the tumors measured <1.5 cm. The localization of the main tumor was cephalic in five cases, corporeal in five, and caudal in eight. One tumor that was located in the splenic hilum probably developed on a pancreatic heterotopia. Two patients had diffuse liver metastases, one with ascites. One patient had a solitary liver metastase. We observed two cases of sinistral portal hypertension due to tumoral thrombosis of the splenic vein.

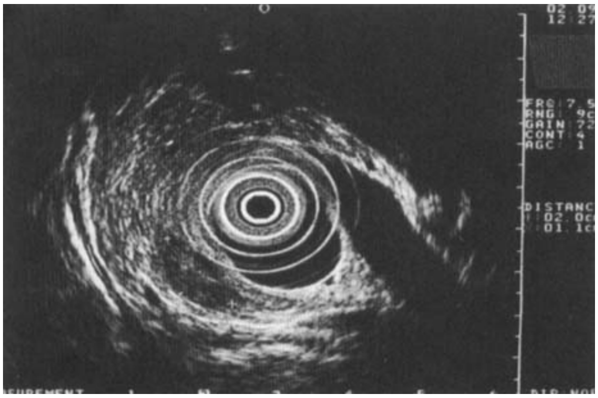

FIG. 1. US image of an insulinoma located at the junction between the head and the uncinate process of the pancreas in a 17-year-old boy. EUS from the duodenum shows an hypoechoic tumor of 20-mm diameter, with regular margins.

Surgery

Benign insulinomas were treated by enucleations ($n = 8$) and distal pancreatectomies ($n = 6$, including 5 without splenectomy). No blind pancreatic resection was performed. For malignant insulinomas, we performed three distal pancreatectomies with regional lymphadenectomy. In one case, liver metastasectomy was associated. The patient with MEN 1 and diffuse tumors had a total pancreatectomy.

No postoperative death occurred, but six patients had surgical complications (33%). One benign external pancreatic fistula resolved itself spontaneously. Two patients had symptomatic residual collections treated by percutaneous drainage. Reoperations were necessary in two cases because of intraabdominal abscesses due to pancreatic fistula. A bulky aneurysm of the splenic artery was reoperated on after distal pancreatectomy with spleen preservation. The morbidity for patients undergoing enucleation was 14% as opposed to 50% for any type of resection ($p < 0.05$).

Pathology

A diagnosis of malignant insulinoma was retained in three cases (16%) because of liver metastases with lymph nodes metastases ($n = 2$) and vascular, perinervous, and peripancreatic fatty tissue invasion ($n = 1$).

Follow-up

All patients (except one lost in the follow-up) with benign tumors were symptom-free and had a normal fast glycemia level after a median follow-up of 18 months (range, 3–120 months). Of the three patients with malignant tumors, one died with liver metastases (15 months), one survived with liver metastases and had a partial response after systemic chemotherapy (31 months), and the third survived without recurrence (44 months).

DISCUSSION

The results of this study suggest that EUS is an excellent method for preoperative localization of insulinomas. Both endoscopic and intraoperative techniques using US can avoid blind pancreatic resection.

Careful operative exploration remains the easiest and one of the most sensitive methods to localize insulinomas (3). However, most surgeons wish to know the exact localization of the tumor, its presumed benignity or malignancy, and the existence of liver metastases before surgery (6). In this study, conventional imaging methods failed to localize 62% of the tumors. The diversity of methods and of their combination represents usual clinical practice as opposed to prospective studies. Transabdominal US and CT correctly localized tumors in 17 and 29% of patients respectively. In other studies, the accuracy of these methods ranges from 0 to 62% for US (7–15) and 12 to 73% for CT (2,3,7,9–15). Preliminary reports of results of MRI were disappointing (11,15). At the beginning of this study, patients suspected of insulinoma had an angiography. Until recently, this method was considered the best procedure for preoperative localization. Several studies have shown 44 to 67% true-positive results (2,3,9,10,12–14,17), but others have had lower success rates (11,15,18), and we were able to locate only 33% of the tumors using angiography. The increasing proportion of small occult insulinomas is probably responsible for these contradictory results (11). We were unable to evaluate critically the efficiency of THVS because of limited experience with this method. Successful localization rates of 64–100% have been reported (2,10,11,13–18), and this procedure is actually considered the best, particularly when all other imaging methods have failed. However, THVS is time-consuming, requires an experienced radiologist, and is sometimes responsible for side effects or complications such as epigastric pain, transient hemobilia (27), hepatic hematoma (28), peritoneal bile leakage (18), or hemorrhage (one personal case). Furthermore, results of THVS are sometimes imprecise and can guide the surgeon only to the head or the tail of the pancreas (3). In our view, THVS could be reserved, prior surgery, in patients for whom a previous operation has failed to resect the tumor and for whom EUS did not localize the insulinoma.

All the patients in this study have been operated on, and surgical resections were always guided by the localization of the tumor. Palpation was clearly positive in 72% of cases and falls within the ranges reported by other authors (64 to 96%) (2–4,9–11,16). IOUS always localized the tumors and was helpful in analyzing their connections with pancreatic and biliary ducts. Similar results have been reported in other studies (9–13,16,17,21,22). In the absence of IOUS, most of the patients might have had pancreatectomies instead of enucleations. IOUS can detect nonpalpable insulinomas because of their small size (usually <1 cm) or because of their deep localization in the head of the pancreas. Benign insulinomas are usually round and regular hypoechoic tumors. Malignant tumors have irregular margins (20). Rarely, the tumor is isoechoic, surrounded by a thin hypoechoic border. In our view IOUS is the best method for localization of insulinomas, and over the last 10 years we have progressively given up performing invasive methods such as angiography and THVS.

EUS is a very sensitive method for diagnosing small pancreatic carcinomas (23). Some observations have reported the usefulness of EUS for the localization of insulinomas (24,29–31). In our study, 90% of the tumors were visualized by EUS. For eight of 11 patients, EUS was the only method that detected the tumor. The tumor's size was also accurately evaluated. At our center, EUS has completely modified the localization strategy since its sensitivity is similar to that of IOUS. If the tumor can be detected before surgery, the need for complete mobilization of the pancreas, which is sometimes dangerous, is avoided. Moreover the nature of the surgical resection can be planned, especially in the case of presumed malignancy. At last, IOUS can be negative at surgery (9–13,16,17,21,22). A multicenter study of the localization of 39 pancreatic endocrine tumors (among which 31 were insulinomas) by EUS has shown an 82% sensitivity and 95% specificity (24). The main reasons for failure were pedonculated tumors and technical problems. Insulinomas are generally hypoechoic and well-demarcated tumors (24,29,30). We found that some benign tumors with an isoechoic pattern could not always be distinguished from the rest of the pancreas. Our observations confirm that a malignant tumor may be suspected by the presence of an irregular margin (30), enlarged lymph nodes (24), or venous invasion with regional portal hypertension. We found that EUS precisely analyzed tumoral connections with the biliary and pancreatic ducts and, therefore, often influenced the type of resec-

tion. No morbidity through the use of EUS has been reported. However, like angiography and THVS, this localization procedure is operator dependent. Various strategies for surgical treatment of insulinomas have been proposed. The simplest one does not require preoperative localization except in those cases that do not respond to diazoxide (3). Other more sophisticated strategies include various invasive methods such as angiography and THVS (15) and selective intraarterial injection of calcium (5) or methylene blue (6). For us, EUS completed by IOUS would dramatically simplify the localization of insulinomas and limit the use of invasive methods to the few cases in which EUS has failed.

Most of our patients were operated on after a trial of medical therapy with diazoxide and frequent feeding. This approach has been recommended to test pharmacological control of organic hypoglycemia in cases of negative intraoperative exploration (2,11). Blind distal two-thirds pancreatectomy has been recommended in cases in which diazoxide does not control hypoglycemia and THVS fails to localize the tumor (3). This procedure has an increased morbidity, with no guarantee that the tumor will be resected (16). EUS and IOUS would make blind resection exceptional. Enucleation of the tumor was our first-choice intervention as recommended by other authors (2,11) even for deep cephalic tumors (three cases in this study). In contrast to other studies (4), we observed less morbidity after enucleation than after pancreatectomy.

In conclusion, we believe that preoperative localization of insulinomas is still necessary. Our results show that EUS is the most accurate noninvasive method. Most tumors can be palpated by experienced hands but IOUS is mandatory. We see no need for other imaging methods except in cases of presumed malignancy.

REFERENCES

1. Comi RJ, Gordon P, Doppman JL, Norton JA. Insulinoma. *In:* Go VLW, Brooks FP, DiMagno EP, Gardner JD, Lebenthal E, Scheele GA, eds. *The exocrine pancreas: biology, pathobiology and diseases.* New York: Raven Press, 1986:745–61.
2. Pasieka JL, McLeod MK, Thompson NW, Burney RE. Surgical approach to insulinomas. *Arch Surg* 1992;127:442–7.
3. Daggett PR, Goodburn EA, Kurtz AB, et al. Is preoperative localisation of insulinomas necessary? *Lancet* 1981;1:483–6.
4. Glickmann MH, Hart MJ, White TT. Insulinoma in Seattle: 39 cases in 30 years. *Am J Surg* 1980;140:119–25.
5. Doppman JL, Miller DL, Chang R, Shawker TH, Gordon P, Norton JA. Insulinomas: localization with selective intraarterial injection of calcium. *Radiology* 1991;178:237–41.
6. Fedorak IJ, Ko TC, Gordon D, Flisak M, Prinz RA. Localization of islet cell tumors of the pancreas: a review of current techniques. *Surgery* 1993;113:242–9.
7. Broughan TA, Leslie JD, Soto JM, Hermann RE. Pancreatic islet cell tumors. *Surgery* 1986;99:671–8.
8. Fraker DL, Norton JA. Localization and resection of islet cell tumors of the pancreas. *JAMA* 1988;259:3601–5.
9. Grant CS, Van Heerden J, Charboneau JW, James EM, Reading CC. Insulinoma: the value of intraoperative ultrasonography. *Arch Surg* 1988;123:843–8.
10. Böttger T, Weber W, Beyer J, Junginger T. Value of tumor localization in patients with insulinoma. *World J Surg* 1990; 14:107–14.
11. Doherty GM, Doppman JL, Shawker TH, et al. Results of a prospective strategy to diagnose, localize, and resect insulinomas. *Surgery* 1991;110:989–97.
12. Galiber AK, Reading CC, Charboneau JW, et al. Localization of pancreatic insulinoma: comparison of pre- and intraoperative US with CT and angiography. *Radiology* 1988;166: 405–8.
13. Proye C, Boissel P. Preoperative imaging versus intraoperative localization of tumors in adult surgical patients with hyperinsulinemia: a multicenter study of 338 patients. *World J Surg* 1988;12:685–90.
14. Rothmund M, Angelini L, Brunt M, et al. Surgery for benign insulinoma: an international review. *World J Surg* 1990;14: 393–9.
15. Vinik AI, Delbridge L, Moattari R, Cho K, Thompson N. Transhepatic portal vein catherization for localization of insulinomas: a ten-year experience. *Surgery* 1991;109:1–11.
16. Norton JA, Shawker TH, Doppman JL, et al. Localization and surgical treatment of occult insulinomas. *Ann Surg* 1990; 212:614–20.
17. Gianello P, Gigot JF, Berthet F, et al. Pre- and intraoperative localization of insulinomas: report of 22 observations. *World J Surg* 1988;12:389–97.
18. Roche A, Raisonnier A, Gillon-Savouret M-C. Pancreatic venous sampling and arteriography in localizing insulinomas and gastrinomas: procedure and results in 55 cases. *Radiology* 1982;145:621–7.
19. Sigel B, Duarte B, Coelho JCU, Nyhus LM, Baker RJ, Machi J. Localization of insulinomas of the pancreas at operation by real-time ultrasound scanning. *Surg Gynecol Obstet* 1983;156:145–7.
20. Norton JA, Cromack DT, Shawker TH, et al. Intraoperative ultrasonographic localization of islet cell tumors. *Ann Surg* 1988;207:160–8.
21. Klotter HJ, Rückert K, Kümmerle F, Rothmund M. The use of intraoperative sonography in endocrine tumors of the pancreas. *World J Surg* 1987;11:635–41.
22. Chapuis Y, Hernigou A, Plainfosse MC, Bonnette P. Exemples d'application de l'ultrasonographie-temps réel peropératoire en chirurgie endocrinienne. *Chirurgie* 1984;110: 92–104.
23. Rösch T, Lorenz R, Braig C, et al. Endoscopic ultrasound in pancreatic tumor diagnosis. *Gastrointest Endosc* 1991;37: 347–52.
24. Rösch T, Lightdale CJ, Botet JF, et al. Localization of pancreatic endocrine tumors by endoscopic ultrasonography. *N Engl J Med* 1992;326:1721–6.
25. Fajans SS, Floyd JC. Fasting hypoglycemia in adults. *N Engl J Med* 1976;294:766–72.
26. Turner RC, Oakley NW, Nabarro JDN. Control of basal insulin secretion, with special reference to the diagnosis of insulinomas. *Br Med J* 1971;2:132–5.

27. Cho KJ, Vinik AI, Thompson NW, et al. Localization of the source of hyperinsulinism: percutaneous transhepatic portal and pancreatic vein catheterization with hormone assay. *AJR* 1982;139:237–45.
28. Ingemanssson S, Kühl C, Larsson LI, Lunderquist A, Lundquist I. Localization of insulinomas and islet cell hyperplasias by pancreatic vein catheterization and insulin assay. *Surg Gynecol Obstet* 1978;146:725–34.
29. Palazzo L, Roseau G, Salmeron M. Endoscopic ultrasonography in the preoperative localization pancreatic endocrine tumors. *Endoscopy* 1992;24:350–3.
30. Hayashi Y, Nakazawa S, Kimoto E, Naito Y, Morita K. Clinicopathologic analysis of endoscopic ultrasonograms in pancreatic mass lesions. *Endoscopy* 1989;21:121–5.
31. Kobayashi M, Araki K, Ogata T. Usefulness of endoscopic ultrasonography for the localization of insulinomas. *Surgery* 1993;113:478.
