# Supplementary material for: Diagnostic value of endoscopic ultrasound for insulinoma localization: A systematic review and meta-analysis
Source: PLoS One. 2018 Oct 23;13(10):e0206099. doi: 10.1371/journal.pone.0206099 (PMC6198953; doi:10.1371/journal.pone.0206099)
Supplement: S2 File — (ZIP) [file pone.0206099.s002.zip › included studies data availability EUS/Endoscopic ultrasound for localisation of islet cell tumors.pdf]

Downloaded from http://gut.bmj.com/ on October 7, 2017 - Published by group.bmj.com

Gut, 1992, 33, 108–110

Endoscopic ultrasound for localisation of islet cell tumours

J R Glover, P J Shorvon, W R Lees

Department of Diagnostic Imaging, Middlesex Hospital, London W1N 8AA
J R Glover
P J Shorvon
W R Lees

Correspondence to:
Dr J R Glover, Department of Diagnostic Imaging, Middlesex Hospital, Mortimer Street, London W1N 8AA.

Accepted for publication 11 March 1991

Abstract

In a prospective study endoscopic ultrasound localisation of pancreatic endocrine tumours was attempted in 21 patients with clinically suspected islet cell tumours. Most patients were referred after the failure of conventional imaging methods. Endoscopic ultrasound correctly identified the site of 12 of 15 insulinomas, one glucagonoma, and a diffuse pancreatic abnormality in a patient with multiple endocrine adenopathy. There were two true negative examinations and one technical failure. The sensitivity of endoscopic ultrasound was much greater than that of computed tomography or conventional transabdominal ultrasonography.

Pancreatic endocrine tumours have well described characteristic clinical presentations,[1,2] but, as there is only limited success with medical management, surgery is usually necessary. Although islet cell tumours are often palpable at surgery,[3] 10% of the insulinomas and 60% of gastrinomas may be multiple or malignant. Therefore, preoperative localisation is desirable to reduce the duration and complexity of the operation. However, the visualisation of functioning pancreatic islet cell tumours has been problematic for most conventional imaging modalities because the lesions are generally small,[4] and they are located in an organ to which there is limited access. Whereas there are enthusiasts for localisation by computed tomography,[5,6] conventional ultrasound,[7,8] angiography,[9] venous sampling,[10,11] and more recently peroperative ultrasound,[3,7–9,12–14] none of these has achieved overall predominance, and reported success rates have not always been reproduced in other centres.[15,16] Endoscopic ultrasound allows the positioning of a high frequency ultrasound transducer in close proximity to the whole length of the pancreas without intervening abdominal wall and bowel to consistently produce high resolution images of the gland.

This study was set up to test the ability of the technique to demonstrate and localise islet cell tumours.

Patients and methods

All the patients were referred for endoscopic

TABLE I *Insulinoma patients*

Patient No	*Sex*	*Age (years)*	*Size of tumour (mm)*	*Endoscopic ultrasound*	*Ultrasound*	*Computed tomography*	*Angiography*	*Venous sampling*	*Other**	*Histology*	*Site*	*Comments*
1	F	76	15	+	–	–	0	0		+	Uncinate	
2	F	45	11	+	–	–	–	0	[1]	+	Neck	
3	M	23	40	–	–	–	0	0	[2]	+	Head	
4	F	16	10	–	–	–	–	–		+	Splenic hilum	
5	F	70	35	+	–	–	0	0		+	Head	
6	M	35	15	+	–	–	0	0		+	Tail	
7	M	55	27,15	+/+	+/–	+/–	0	0	[3]	+	Neck and body	Two lesions
8	M	69	7	+	–	–	0	0		0	Head	Patient died, no necropsy
9	M	76	15	+	–	–	0	0		+	Tail	Computed tomography+ retrospectively
10	F	41	10	+	+	+	0	0		+	Head	
11	M	62	11	+	–	–	–	0		+	Uncinate	Angiography located lesion in head
12	M	60	8	–	–	–	0	0		+	Splenic hilum	Endoscopic ultrasound showed reactive node in body
13	F	54	6	+	–	+	0	0		0	Neck	Not found surgery or at necropsy
14	M	73	6	+	–	–	–	0		+	Body	
15	M	49	20	+	–	–	0	0		+	Neck	
16	F	31	8	+	–	–	–	–		+	Body	

*1=magnetic resonance imaging also negative; 2=previous partial pancreatectomy. Computed tomography and angiography showed the lesion at the splenic hilum but it was interpreted as a splenunculus; 3=both shown in percutaneous pancreatogram.
+=true positive; –=negative; 0=not performed.

TABLE II *Non-insulinoma patients*

Patient No	*Sex*	*Age (years)*	*Size of tumour (mm)*	*Endoscopic ultrasound*	*Ultrasound*	*Computed tomography*	*Angiography*	*Venous sampling*	*Other*	*Histology*	*Site*	*Hormone*	*Comments*
17	M	29	20	+	–	+	0	0		+	Tail	Glucagon	Three others 5 mm missed
18	F	53	–	–	–	–	0	0		0	0	Gastrin	Spont.–>Normal
19	M	32	–	0	–	–	0	0		0	0	Gastrin	Technical failure
20	F	47	–	–	–	–	0	0		0	0	Vasoactive intestinal polypeptide	Biochemistry normal
21	F	39	Diffuse	+	+	–	0	0		0	All	Inactive	Multiple endocrine adenopathy

+=true positive; –=negative; 0=not performed.

Downloaded from http://gut.bmj.com/ on October 7, 2017 - Published by group.bmj.com

TABLE III *Endoscopic ultrasound* v *other investigations (insulinomas)*

	Angiography	*Computed tomography*	*Ultrasound*	*Venous sampling*	*Endoscopic ultrasound*
No of patients	5	14	14	2	14
True positive	0	3	2	0	11
False positive*	1	0	0	0	1
Sensitivity	0	21%	14%	0	79%

*Lesion incorrectly identified as insulinoma.

ultrasound imaging of the pancreas from this and other hospitals over a 30 month period. Most referrals were due to failure of other imaging techniques. All patients had biochemical evidence or there was a strong clinical suspicion of islet cell tumours. Sixteen patients had biochemical evidence of insulinomas (Table I), two had suspected gastrinomas, and one each suspected glucagonoma, vipoma, and multiple endocrine adenopathy (Table II). Ten patients were women and 11 were men, mean age 49 years (range 16–76 years). All had previously been investigated by a minimum of high resolution computed tomography and conventional transabdominal ultrasound. The endoscopic ultrasound probe used has a 7·5 MHz rotating transducer attached to the end of a side viewing endoscope (Olympus GF-UM2 Ultrasonic Endoscope, Keymed, Southend, UK). The transducer rotates at 10 revolutions per second producing a realtime 360° radial image at right angles to the long axis of the endoscope. It is passed down to the duodenal loop under direct vision after intravenous sedation and nasopharyngeal local anaesthesia, as for conventional endoscopy. Once in the descending duodenal loop air is sucked out of the bowel and a balloon covering the transducer is distended with water to facilitate maximum contact and acoustic coupling with the mucosa. The endoscope with attached transducer is then slowly withdrawn through the duodenum, pylorus, and stomach to the oesophageal hiatus, stopping at any suspicious lesions for careful local scanning, measurement, and accurate localisation of the lesion. This can easily be repeated if necessary. After recovery from the sedation patients were discharged the same day, unless their underlying clinical state required continued hospitalisation or admission. The procedure took 10–30 minutes and was no more uncomfortable for patients than conventional fibreoptic gastroduodenoscopy.

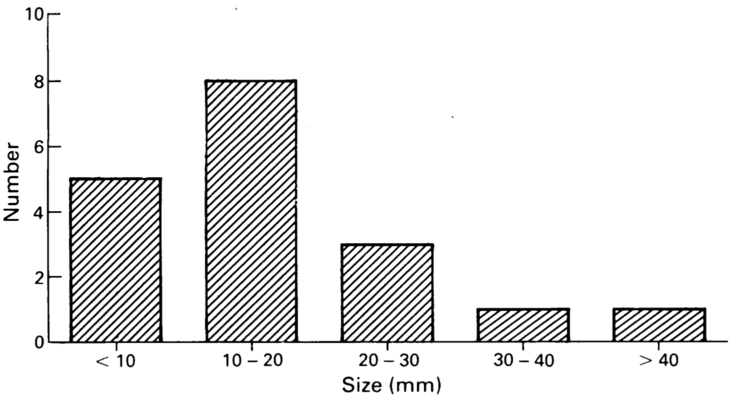

Figure 1: Size distribution of insulinomas.

Results

Of the 16 patients with biochemically suspected insulinomas, positive histology was obtained in 14 (Table I). There was no predilection for any single site in the pancreas. One patient with convincing biochemical evidence of insulinoma died of fulminating pancreatitis after surgery at which no tumour could be found, and no lesion was identified at subsequent necropsy, although endoscopic ultrasound had shown a 6 mm lesion in the neck of the pancreas (case 13). Another patient died without surgery and necropsy was refused (case 8). There were 15 tumours in the remaining 14 insulinoma patients, 12 of which were correctly identified and localised by endoscopic ultrasound. The three lesions missed consisted of a 40×15 mm lesion in the head, subsequently identified by intraoperative ultrasound, and two lesions in the splenic hilum. One of these was in a patient who had had a distal pancreatectomy. Of the remaining five patients, there was one technical failure due to duodenal scarring which prevented passage of the scope beyond the pylorus in a patient with the Zollinger-Ellison syndrome (case 19). There were two true negatives, one in a patient with suspected gastrinoma in whom the levels subsequently spontaneously returned to normal, and one in a patient with symptoms of a recurrent vipoma in whom blood levels of vasoactive intestinal polypeptide consistently remained normal. One patient had a 20 mm glucagonoma shown on endoscopic ultrasound but three further 5 mm lesions were also found at surgery which were not shown by endoscopic ultrasound. The patient with multiple endocrine adenopathy syndrome was shown to have a diffusely abnormal pancreas with multiple small tumours.

Conventional ultrasound detected only two islet cell tumours, and showed diffuse pancreatic abnormality in the patient with multiple endocrine adenopathy type 1. Computed tomography identified three insulinomas and showed the 20 mm glucagonoma.

The site of five lesions was suspected on conventional imaging before endoscopic ultrasound. No adverse effects were noted in any of the patients after endoscopic ultrasound.

Discussion

Most patients in this series were referred for endoscopic ultrasound because of the failure of other imaging modalities, which is reflected in the very low sensitivities of computed tomography (25%) and ultrasound (18%) (Table III) compared with other series.[5-8] Most of the lesions were less than 19 mm and five were less than 9 mm (Fig 1).

Some investigators advocate computed tomography and selective angiography for preoperative localisation of pancreatic endocrine tumours,[6] but angiography is more invasive than endoscopic ultrasound and has a greater risk of adverse events. In this series six of the insulinoma patients were investigated by angiography and computed tomography but none of the lesions was correctly located, and one of the lesions (case 11) was localised to the head rather

Downloaded from http://gut.bmj.com/ on October 7, 2017 - Published by group.bmj.com

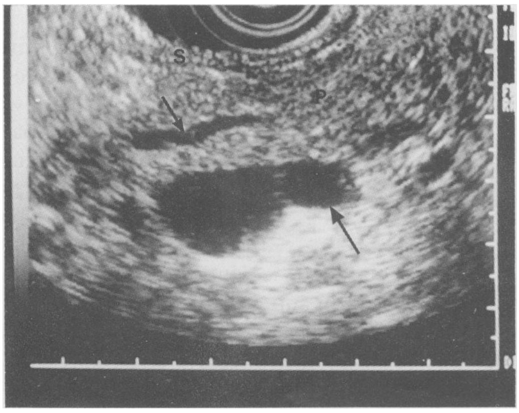

Figure 2: Transverse section through the body of the pancreas showing normal pancreatic tissue (P) and duct (small arrow) in front of splenic vein (large arrow). S=stomach wall.

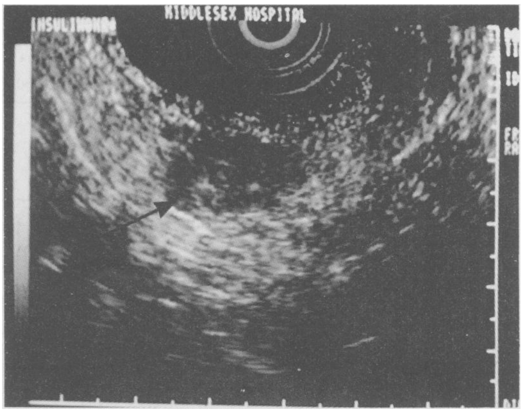

Figure 3: Well circumscribed insulinoma (arrowed) in the tail of the pancreas showing a complex internal low level echo pattern.

than the uncinate lobe and could not be found at subsequent laparotomy performed before endoscopic ultrasound.

Endoscopic ultrasound is safe and non-invasive compared with angiography and venous sampling. The depth of penetration of the ultrasound beam is limited to approximately 6 cm from the transducer, and it produces radial images which require experience to orientate and interpret (Fig 2). Islet cell tumours generally appear as well circumscribed lesions, hypoechoic compared with the surrounding normal pancreas (Fig 3).

A proven alternative is peroperative ultrasound scanning after full mobilisation of the pancreas. In recent reports peroperative ultrasound combined with surgical palpation has produced a sensitivity of up to 100%.[8 14] Peroperative scanning may lead the surgeon directly to the lesion but it prolongs surgery and requires a confident preoperative diagnosis which is often difficult to achieve. Localisation by endoscopic ultrasound can greatly reduce the extent of surgical mobilisation required and size of resection. Ultrasonic endoscopes are wider than simple side viewing scopes, with a longer non-flexible tip which can make entry into the duodenum difficult, especially if there is scarring (see case 19). However, future ultrasonic endoscopes are likely to be narrower and more flexible. As well as showing pancreatic lesions as small as 6 mm, endoscopic ultrasound also shows the bowel wall with great accuracy, which is important in gastrinoma patients whose lesions may be in the duodenal wall. Endoscopic ultrasound may have a blind spot in the region of the splenic hilum but so may other imaging modalities, as case 2 shows.

We believe that endoscopic ultrasound is a useful adjunct to the investigation and localisation of islet cell tumours, which will replace other more invasive techniques, and we recommend it as the next investigation after computed tomography and ultrasound. As pancreatic endocrine tumours may be small and multiple it should be performed in all such patients or small functioning lesions may be missed, particularly if they coexist with larger lesions visible with other modalities.

1 Turner RC. Hypoglycaemia. In: Wetherall DJ, Ledingham JGG, Warell, DA, eds. *Oxford textbook of medicine.* 2nd ed. Oxford: Oxford Medical Publications, 1987: 9.102–8.
2 Bloom SR, Long RG, Polak JMO. Hormones and the gastro-intestinal tract. In: Wetherall DJ, Ledingham JGG, Warell DA, eds. *Oxford textbook of medicine.* 2nd ed. Oxford: Oxford Medical Publications, 1987: 12.52–61.
3 Harrison TS. The hypoglycemic syndrome, endogenous hyperinsulinism. In: Friesen SR, Bolinger RE, eds. *Surgical endocrinology.* Philadelphia: JB Lippincott, 1978: 150–9.
4 Grant CS, van Heerden JA, Charboneau JW, *et al.* Insulinoma – the value of intraoperative ultrasonography. *Arch Surg* 1988; **123:** 843–9.
5 Wank SA, Doppman JL, Miller DL, *et al.* Prospective study of the ability of computed axial tomography to localize gastrinomas in patients with Zollinger-Ellison syndrome. *Gastroenterology* 1987; **92:** 905–12.
6 Rossi P, Allison DJ, Bezzi M, *et al.* Endocrine tumors of the pancreas. *Radiol Clin North Am* 1989; **27:** 129–61.
7 Gorman B, Charboneau JW, James EM, *et al.* Benign pancreatic insulinoma: preoperative and intraoperative localization. *AJR* 1986; **147:** 929–34.
8 Galiber AK, Reading CC, Charboneau JW, *et al.* Localization of pancreatic insulinoma: comparison of pre- and intraoperative US with CT and angiography. *Radiology* 1988; **166:** 405–8.
9 Gianello P, Gigot JF, Berthet F, *et al.* Pre- and intraoperative localization of insulinomas: report of 22 observations. *World J Surg* 1988; **12:** 389–97.
10 Doppman JL, Brennan MF, Dunnick NR, *et al.* The role of pancreatic venous sampling in the localization of occult insulinomas. *Radiology* 1981; **138:** 557–62.
11 Parkman HP, Malet PF, Ogorek CP, *et al.* Preoperative localization of a vasoactive intestinal peptide-secreting tumor by transhepatic portal venous sampling. *Am J Gastroenterol* 1988; **83:** 559–63.
12 Sigel B, Duarte B, Coelho JCU, *et al.* Localization of insulinomas of the pancreas at operation by real-time ultrasound scanning. *Surg Gynecol Obstet* 1983; **156:** 145–7.
13 Charboneau JW, James EM, van Heerden JA, *et al.* Intraoperative real-time ultrasonographic localization of pancreatic insulinoma: initial experience. *J Ultrasound Med* 1983; **2:** 251–4.
14 Norton JA, Cromack DT, Shawker TH, *et al.* Intraoperative ultrasonographic localization of islet cell tumours – a prospective comparison to palpation. *Ann Surg* 1988; **207:** 160–8.
15 Frucht H, Doppman JL, Norton JA, *et al.* Gastrinomas: comparison of MR imaging with CT, angiography, and US. *Radiology* 1989; **171:** 713–7.

Downloaded from http://gut.bmj.com/ on October 7, 2017 - Published by group.bmj.com

GUT

Endoscopic ultrasound for localisation of islet cell tumours.

J R Glover, P J Shorvon and W R Lees

Gut 1992 33: 108-110
doi: 10.1136/gut.33.1.108

Updated information and services can be found at:
http://gut.bmj.com/content/33/1/108

These include:

Email alerting service

Receive free email alerts when new articles cite this article. Sign up in the box at the top right corner of the online article.

Topic Collections

Articles on similar topics can be found in the following collections

Pancreas and biliary tract (1949)

Notes

To request permissions go to:
http://group.bmj.com/group/rights-licensing/permissions

To order reprints go to:
http://journals.bmj.com/cgi/reprintform

To subscribe to BMJ go to:
http://group.bmj.com/subscribe/
