# Supplementary material for: Diagnostic value of endoscopic ultrasound for insulinoma localization: A systematic review and meta-analysis
Source: PLoS One. 2018 Oct 23;13(10):e0206099. doi: 10.1371/journal.pone.0206099 (PMC6198953; doi:10.1371/journal.pone.0206099)
Supplement: S2 File — (ZIP) [file pone.0206099.s002.zip › included studies data availability EUS/Endoscopic Ultrasound Is Highly.pdf]

THE AMERICAN JOURNAL OF GASTROENTEROLOGY
© 2000 by Am. Coll. of Gastroenterology
Published by Elsevier Science Inc.

Vol. 95, No. 9, 2000
ISSN 0002-9270/00/$20.00
PII S0002-9270(00)01272-7

Endoscopic Ultrasound Is Highly Accurate and Directs Management in Patients With Neuroendocrine Tumors of the Pancreas

Michelle A. Anderson, M.D., Steven Carpenter, M.D., Norman W. Thompson, M.D., Timothy T. Nostrant, M.D., F.A.C.G., Grace H. Elta, M.D., F.A.C.G., and James M. Scheiman, M.D., F.A.C.G.

Division of Gastroenterology, Department of Internal Medicine and Division of Endocrine Surgery, Department of Surgery, University of Michigan School of Medicine, Ann Arbor, Michigan

OBJECTIVE: Preoperative localization of pancreatic neuroendocrine tumors with traditional imaging fails in 40–60% of patients. Endoscopic ultrasound (EUS) is highly sensitive in the detection of these tumors. Previous reports included relatively few patients or required the collaboration of multiple centers. We report the results of EUS evaluation of 82 patients with pancreatic neuroendocrine tumors.

METHODS: We prospectively used EUS early in the diagnostic evaluation of patients with biochemical or clinical evidence of neuroendocrine tumors. Patients had surgical confirmation of tumor localization or clinical follow-up of >1 yr.

RESULTS: Eighty-two patients underwent 91 examinations (cases). Thirty patients had multiple endocrine neoplasia syndrome type I. One hundred pancreatic tumors were visualized by EUS in 54 different patients. The remaining 28 patients had no pancreatic tumor or an extrapancreatic tumor. Surgical/pathological confirmation was obtained in 75 patients. The mean tumor diameter was 1.51 cm and 71% of the tumors were ≤2.0 cm in diameter. Of the 54 explorations with surgical confirmation of a pancreatic tumor, EUS correctly localized the tumor in 50 patients (93%). Twenty-nine insulinomas, 18 gastrinomas, as well as one glucagonoma, one carcinoid tumor, and one somatostatinoma were localized. The most common site for tumor localization was the pancreatic head (46 patients). Most tumors were hypoechoic, homogenous, and had distinct margins. EUS of the pancreas was correctly negative in 20 of 21 patients (specificity, 95%). EUS was more accurate than angiography with or without stimulation testing (secretin for gastrinoma, calcium for insulinoma), transcutaneous ultrasound, and CT in those patients undergoing further imaging procedures. EUS was not reliable in localizing extrapancreatic tumors.

CONCLUSIONS: In this series, the largest single center experience reported to date, EUS had an overall sensitivity and accuracy of 93% for pancreatic neuroendocrine tumors. Our results support the use of EUS as a primary diagnostic modality in the evaluation and management of patients with neuroendocrine tumors of the pancreas. (Am J Gastroenterol 2000;95:2271–2277. © 2000 by Am. Coll. of Gastroenterology)

INTRODUCTION

Although neuroendocrine tumors of the pancreas develop in relatively few patients, their small size and protean clinical manifestations both fascinate and frustrate clinicians. They occur with a prevalence of approximately 10 per 1 million persons, usually presenting in the fourth or fifth decade of life. The tumors cause distinct clinical syndromes associated with an elevated hormone level, in the case of functioning tumors, or with tumor mass effects in the case of nonfunctioning tumors (1). Endocrine tumors of the pancreas may produce a variety of hormones including insulin, gastrin, pancreatic polypeptide, vasoactive intestinal peptide, and somatostatin. The most common clinical syndrome is caused by insulinomas, which are located in the pancreas in >90% of patients (1), followed by gastrinomas, which are extrapancreatic in location in up to 44% of patients (2).

Approximately one-half of neuroendocrine tumors are malignant, a designation based on the presence of metastases as well as vascular or lymphatic invasion rather than tumor histology. Even in the case of malignant disease, the prognosis for patients with neuroendocrine tumors is far better than their counterparts with pancreatic adenocarcinoma and a significant number of patients can expect cure with surgical resection (3, 4). This feature, coupled with the fact that many of the tumors are very small, typically <2 cm, make preoperative localization both critical and highly challenging (5).

Preoperative localization studies using extracorporeal ultrasound, CT, and angiography fail to localize the primary tumor in 40–60% of patient, and frequently miss small tumors (<2 cm) (5, 6). Recently, arterial stimulation of the tumor with the appropriate secretagogue followed by selective portal venous sampling, has improved localization sen-

sitivity and specificity. Doppman and colleagues (7) reported a sensitivity of 88% for insulinoma localization using intra-arterial calcium stimulation, a result that surpassed abdominal ultrasound, CT, MRI), arteriography, and portal venous sampling, which had sensitivities of 9%, 17%, 43%, 36%, and 67%, respectively. Analogous studies by the same group using secretin injection for gastrinoma detection demonstrated reduced sensitivity (41%) but high specificity (98%) and was believed to have assisted the clinical management of 22% of patients (8).

Recent studies using somatostatin receptor scintigraphy have noted impressively high detection rates for neuroendocrine tumors (9–13). However, it is often not possible to differentiate uptake in pancreatic glandular tissue *versus* an adjacent lymph node, nor does the test localize the tumor to a specific anatomical region of the pancreas (11, 12). Another limitation of the study results is that up to 20% of insulinomas do not express somatostatin receptors, making the use of this imaging modality not useful in those patients (11, 12, 14, 15).

The use of endoscopic ultrasound (EUS) in the diagnosis and localization of pancreatic neuroendocrine tumors has become increasingly routine. It is relatively noninvasive with low morbidity and essentially no mortality. In a multicenter retrospective study, Rosch and colleagues (16) reported a sensitivity of 82% (32 of 39 patients) using radial scanning endoscopic ultrasound to localize pancreatic neuroendocrine tumors, a result that was significantly more sensitive than angiography (sensitivity, 27%). Although some centers have reported sensitivities of nearly 100% with the use of intraoperative ultrasound in combination with palpation by the surgeon, many surgeons find this technique cumbersome and associated with prolonged operating room time (17, 18, 20, 21).

Rapidly after the introduction of EUS at our center, the procedure has been prospectively used in all patients with suspected pancreatic neuroendocrine tumor early in the preoperative evaluation strategy (22). This report evaluates our results using EUS in the diagnosis, localization, and management of 82 patients with suspected or confirmed pancreatic neuroendocrine tumors.

PATIENTS AND METHODS

Patient Population

We prospectively performed 91 examinations in 82 patients during the period from March 1993 to April 1998 who were referred to our institution with biochemical, radiological, or clinical evidence of neuroendocrine tumors. EUS was used as the initial imaging technique per our previously published management algorithm (22). Thirty-nine men and 43 women with an average age of 46.3 yr (range, 16–79 yr) were included. Forty-six examinations were done for suspected gastrinoma; of these, 30 were in patients with known or suspected multiple endocrine neoplasia type I (MEN I) syndrome. Thirty-eight studies were performed for suspected insulinoma, two for nonfunctioning islet cell tumors, two for carcinoid tumors, one each for suspected somatostatinoma, glucagonoma, and nesidioblastosis. Confirmation of the final diagnosis was obtained, usually by surgical pathology, in 75 of 91 patients. Results from additional imaging studies, such as transabdominal ultrasound, CT, angiography, selective arterial secretin infusion (for gastrinomas), intra-arterial calcium stimulation (for insulinomas), somatostatin receptor scintigraphy, and portal venous sampling studies, in most cases performed within our institution after EUS, were reviewed. The endosonographer was not blinded to the results of previous imaging studies, which typically failed to identify a lesion.

Endoscopic Ultrasound Imaging

Patients with insulinoma underwent EUS as the initial, and often the only, preoperative imaging study, whereas patients with gastrinoma underwent CT scanning to detect metastatic disease, as previously described (22). EUS was performed with either the Olympus UM-20 (Olympus America, Inc., Lake Success, NY) or the Pentax FG 32 UA (Pentax Precision Instruments Corp., Orangeburg, NY) echoendoscopes. Eighty-five of the 91 examinations were believed to be complete. Of the five incomplete examinations, three were attributable to lack of or poor visualization of the tail region of the pancreas, one because of poor visualization of the uncinate process, and one examination was limited by the fact that the tumor discovered was too large to be adequately imaged. A single experienced endosonographer (JMS) performed >95% of the examinations. There was no difference in the accuracy of tumor localization with either instrument. Twelve examinations were performed using the Pentax linear array echoendoscope and 79 were performed using the Olympus radial array echoendoscope. Before the EUS examination, a routine upper endoscopy was performed in patients with gastrinoma to rule out submucosal tumors of the upper GI tract.

The echoendoscope was advanced to the descending duodenum beyond the major ampulla and slowly withdrawn from the duodenum to the stomach. To ensure that all regions of the gland were visualized, regional anatomy of the pancreas was verified by its relationship to the surrounding vessels and organs. The tumor's size, echotexture, location within the pancreas, involvement of the peripancreatic vessels, and the presence of regional lymph nodes were documented and compared to surgical findings. All examinations required approximately 30 to 60 min to complete. Conscious sedation was achieved with intravenous meperidine and diazepam.

If EUS results were negative and clinical suspicion warranted further investigation, patients underwent selective angiography with venous sampling to localize the tumor. The accuracy of tumor localization was based on operative description and pathological confirmation of the tumor. Clinical outcome was determined by review of postoperative visits in the medical record. Cure was defined as nor-

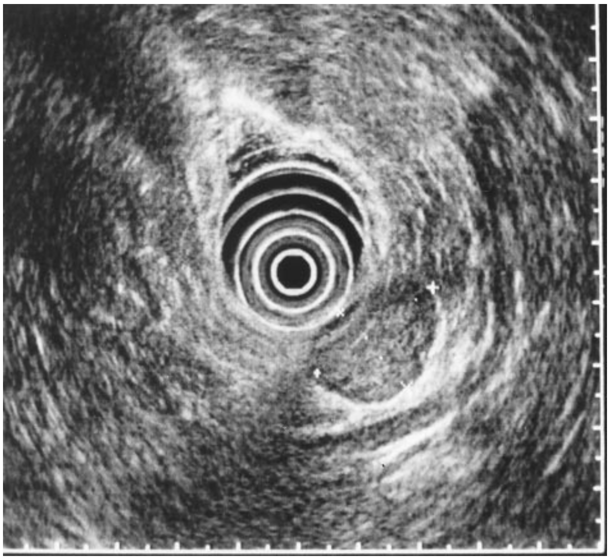

Figure 1. EUS appearance of a pancreatic neck 1.5 by 2.4-cm gastrinoma, imaged at a frequency of 7.5 MHz with the Olympus UM-20 echoendoscope positioned in the stomach.

Table 1. Localization of Pancreatic Neuroendocrine Tumors: The University of Michigan Experience. Preoperative EUS *vs* Final Diagnosis

	All* Tumors (n = 75)	Suspected Gastrinoma (n = 36)	Suspected Insulinoma† (n = 36)
Sensitivity	93%	100%	88%
Specificity	95%	94%	100%
PPV	98%	95%	100%
NPV	83%	100%	43%
Accuracy	93%	97%	89%

PPV = positive predictive value; NPV = negative predictive value.

* Includes one each: glucagonoma, somatostatinoma, and carcinoid tumor.

† Includes three patients with negative EUS: two with probable MEN I and nesidioblastosis, the third with repeat testing inconsistent with insulinoma.

malization of biochemical abnormalities and resolution of the relevant clinical syndrome. Statistical analysis used the χ^2 test with Yates' correction.

RESULTS

Endoscopic Ultrasound Imaging

Eighty-two patients underwent a total of 91 examinations (cases) by EUS. Nine patients underwent two EUS examinations because of recurrent clinical or biochemical abnormalities. One hundred tumors were visualized in 54 different patients, in 38 patients no tumor was found in the pancreas by EUS. The mean diameter of all tumors seen by EUS was 1.51 cm. The smallest lesion measured 0.4 cm and the largest 4.8 cm; seventy-one tumors (71%) were 2.0 cm or less in diameter.

The performance characteristics of EUS were defined by the results of 75 patients with definitive confirmation of tumor status. On the basis of the results of surgery, EUS correctly localized the pancreatic tumor in 50 of 54 patients (93%) in which tumors were present. Eighteen of 18 gastrinomas (Fig. 1), 29 of 33 insulinomas (Fig. 2), as well as one glucagonoma, one carcinoid tumor, and one somatostatinoma were localized by EUS. In a patient with MEN I syndrome, EUS identified two pancreatic tumors producing pancreatic polypeptide and vasoactive intestinal peptide. Table 1 reports the performance characteristics of EUS based on clinical syndrome. The specificity of EUS for pancreatic gastrinomas is quite high (94%), as is its accu-

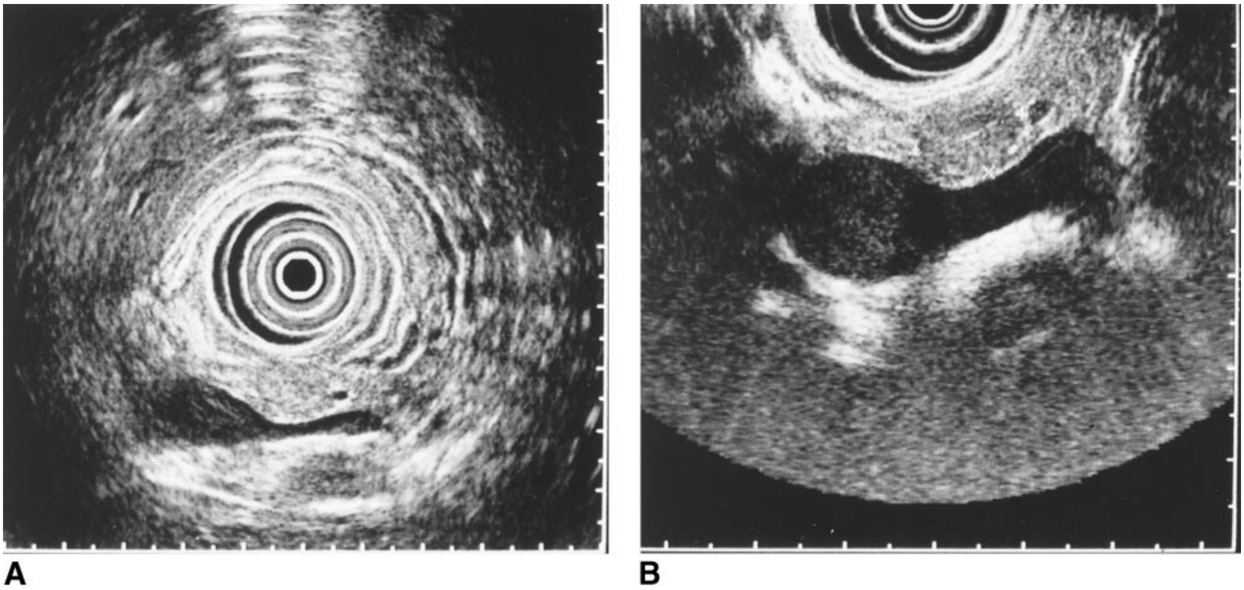

Figure 2. *(A)* EUS image of a 1.0 by 1.1-cm insulinoma in the pancreatic body seen with the Olympus UM-20 echoendoscope at a frequency setting of 7.5 MHz. *(B)* EUS appearance of the tumor in *(A)* imaged with the Olympus UM-20 echoendoscope at 12 MHz. The higher frequency and image magnification allows recognition of the subtle isoechoic nature of the tumor.

Table 2. Tumor Location in the Pancreas as Determined by EUS

Tumor Type	Head/Neck	Body	Tail	Uncinate
Gastrinoma	27	18	8	8
Insulinoma†	18	6	8	4
All*	46	24	16	13

* Combined data for all tumor types; does not include one patient with an ampullary tumor.
† Includes multiple tumors in some patients.

racy (97%). Furthermore, a negative EUS quite reliably predicted an extrapancreatic location for the tumor. Conversely, in patients with suspected insulinomas, a negative EUS examination is less reliable in excluding pancreatic disease (negative predictive value, 43%) and is a function of the nearly uniform pancreatic location for these tumors.

The most common location for the visualized tumors was the pancreatic head region (46) followed by the body (24) (Table 2). Overall, most tumors were hypoechoic, homogenous, and had distinct margins (Table 3). One tumor, an insulinoma, was hyperechoic with irregular margins. Only one ampullary neuroendocrine tumor was visualized. This tumor stained positive for somatostatin on immunohistochemistry and was hypoechoic, homogenous with smooth borders. The only glucagonoma detected had similar EUS features.

Three patients with multiple masses detected on EUS had additional tumors found on surgical excision. These were all in patients with multiple endocrine neoplasia and all additional tumors were very small ($<$2 mm). Despite this, EUS accurately directed surgical excision and all three patients are doing well $\geq$12 months after the procedure.

Among the 36 patients with gastrinoma, diagnostic endoscopy identified a tumor in the stomach or duodenum in four patients (11%). Three of these patients had the MEN I syndrome, whereas one had sporadic disease.

Table 3. Ultrasonographic Features of the Pancreatic Endocrine Tumors Detected by Endoscopic Ultrasound

Feature	Number of Tumors (%)
Insulinomas	36
Echogenic pattern	
Hypoechoic	28 (78)
Isoechoic	7 (19)
Hyperechoic	1 (3)
Ultrasonographic texture	
Homogenous	32 (89)
Inhomogenous	4 (11)
Tumor margin	
Smooth	29 (81)
Irregular	7 (9)
Gastrinomas	61
Echogenic pattern	
Hypoechoic	59 (97)
Isoechoic	2 (3)
Hyperechoic	0
Ultrasonographic texture	
Homogenous	59 (97)
Inhomogenous	2 (3)
Tumor margins	
Smooth	59 (97)
Irregular	2 (3)

Endoscopic Ultrasound Imaging Versus Alternative Imaging Modalities

Of the 14 patients who underwent angiography, a tumor, as depicted by a blush in the respective region of the pancreas, was seen in only four patients (44%). Endoscopic ultrasonography proved significantly more accurate and was positive in 13 of these 14 patients (93%) ($p < 0.05$ by χ^2 test). Only one tumor detected by angiography was not detected by EUS. Alternatively, EUS detected a tumor in four patients who had negative angiographic results.

Of the eight patients who underwent selective angiography with calcium infusion, a tumor, detected by a stepwise increase in insulin concentration in the portal venous system after calcium stimulation, was correctly identified in seven of eight patients (accuracy, 88%). Three additional tumors were detected that were not detected with EUS; however, in two patients testing was falsely positive and in two, testing agreed with EUS results.

Of the nine patients with suspected gastrinomas who underwent angiography, one tumor was correctly localized and the other eight were correctly negative giving a sensitivity and specificity for both of 100%. The one tumor visualized by angiography was also detected on EUS. Therefore, no additional tumors were detected by angiography, which were not localized by EUS.

Eight patients with suspected gastrinomas also underwent selective angiography with secretin infusion (SASI). Of these, SASI incorrectly suggested tumor presence in seven patients and was correctly negative in one patient (specificity, 12%). No additional tumors were detected using SASI that were not seen on EUS. In one patient, selective angiography with secretin infusion was performed after negative EUS and negative angiography and appeared to be positive. Both EUS and angiography were repeated and were again negative. A second SASI was then negative and no pancreatic tumor was found on subsequent surgical exploration.

Endoscopic ultrasonography of the pancreas was correctly negative in 20 of 21 patients (specificity, 95%). The single patient considered to have a false-positive EUS had MEN I syndrome with an elevated serum gastrin. No additional studies, for example, selective angiography with secretin infusion, were performed before surgery. On exploration, the lesions visualized on EUS were found to be cysts and no pancreatic mass was detected.

Patient Outcome

Follow-up data ranging from 9 to 60 months was available in $>$90% of patients. Of the patients with suspected MEN I syndrome, $>$90% with a positive EUS underwent surgery, typically a distal pancreatectomy with enucleation of proximal gland tumors, which resulted in cure as determined by resolution of the clinical syndrome and normalization of laboratory values. One patient with suspected MEN I syn-

drome and a negative EUS was explored and a peripancreatic lymph node positive for gastrinoma was removed. This node was identified on preoperative EUS. Because MEN I syndrome patients may continue to develop new tumors, these "cures" must be considered an outcome of uncertain duration because of the underlying genetic defect.

Three patients with a negative EUS also had negative surgical exploration. Of the patients who underwent EUS for suspicion of sporadic gastrinoma and then went on to laparotomy, cure was obtained in nearly 80% of patients. Three patients with sporadic gastrinoma and negative EUS had no findings on surgical exploration and have been managed medically. All patients with suspected insulinomas who had positive EUS findings were explored and cured. One patient with a negative EUS was found to have an extrapancreatic tumor at laparotomy. This was resected and has resulted in cure.

The largest tumor imaged by EUS, a nonfunctioning islet cell tumor, measured 4.8 by 3.9 cm and was located in the head region of the pancreatic gland. Initially, this patient presented with an episode of abdominal pain and a preliminary CT scan revealed a large pancreatic head mass. Despite concern over possible superior mesenteric artery invasion, a Whipple procedure was attempted because of the patient's young age (47 yr). On surgical exploration the tumor was found to invade only the superior mesenteric vein sparing the superior mesenteric artery and the portal vein. A successful Whipple procedure with partial resection of the involved portion of the superior mesenteric vein and primary anastomosis was performed. The patient remains well with no clinical or radiographic recurrence of the tumor >18 months after surgery.

DISCUSSION

Currently, there is a multitude of potential imaging methods available for the localization of neuroendocrine tumors of the pancreas. EUS has the ability of producing high-resolution images of the pancreas and surrounding structures. It has the added advantage over conventional imaging techniques, such as CT or MRI, of recognizing even small tumors (1 cm or less) with remarkable accuracy. Arterial stimulation with venous sampling, using either secretin or calcium, is invasive, technically demanding, and expensive.

Previous studies evaluating the usefulness of EUS reported a high level of sensitivity in the diagnosis of neuroendocrine tumors of the pancreas, but included relatively few patients or required the collaboration of multiple centers. (5, 16, 18, 20, 21, 23–25) This adds the potential confounding factors of varying operator experience and different imaging techniques from center to center, issues that are known to have a significant impact on image quality and, hence, test sensitivity. This study presents our experience with the use of EUS for localizing neuroendocrine tumors of the pancreas and currently is the largest published single center experience worldwide.

We have previously reported, in abstract form, that EUS had an overall sensitivity of 83% and an accuracy of 89% for pancreatic neuroendocrine tumors and 100% accuracy in excluding pancreatic gastrinoma. Our current analysis is consistent with these previous observations with sensitivity of 93% and overall accuracy of 93% for all neuroendocrine tumor types combined. Our sensitivity for pancreatic gastrinomas continues to be 100%, a figure seldom duplicated by other imaging modalities. As previously stated, a significant proportion of gastrinomas lie outside the pancreas and it is our practice to routinely perform upper endoscopy before EUS in patients with a suspected gastrinoma. We identified additional tumors in 11% of patients with this approach. A negative EUS of the pancreas invariably predicted an extrapancreatic location and helped to direct the surgical exploration for the tumor.

Endoscopic ultrasonography detected 29 of 34 pancreatic insulinomas giving sensitivity slightly below that of the calcium stimulation but a much higher specificity. In this series, the calcium stimulation test gave false-positive results in two of seven patients; these results may have misguided surgery. For insulinomas, EUS performed significantly better than conventional angiography, which had a sensitivity of only 44%. Furthermore, we report a high positive predictive value (100%) but a low negative predictive value (43%). This can be explained in part by the prestudy selection bias making the prevalence of insulinoma in our study population quite high. The majority of the false-negative EUS examinations occurred early in our experience, supporting the value of previous procedural experience.

Of the four false-negative EUS findings, all occurred in patients with insulinomas. In the first patient, the pancreatic head was believed to be "inhomogenous" without a discreet mass. Selective angiography with calcium infusion was performed in this patient and was negative as well. During the initial surgery, no discrete mass was appreciated but head resection did show positive immunohistochemistry for insulinoma. This patient developed postsurgical hypoglycemia and on repeated exploration a Whipple procedure was done revealing a 3-cm mass. In the second patient, EUS was believed to be suboptimal because of retroperitoneal fat, but did not reveal a discrete pancreatic mass. A subsequent angiography with calcium infusion was positive; however, a repeat EUS was suboptimal and again did not show a pancreatic lesion. On surgical exploration, the patient was found to have a pedunculated insulinoma arising from the head region of the pancreas. In the case of the most recent false-negative EUS, both selective arterial angiography and angiography with calcium infusion were positive. At surgery, the patient was found to have a 1.2-cm tumor within the uncinate process 3 cm from the wall of the duodenum. A CT scan performed before EUS was also falsely negative. We believe that all of our false-positive results are a function of the individual tumor characteristics or location.

Recently, somatostatin receptor scintigraphy (SRS) has

undergone technical improvements and has gained more widespread use in the diagnosis and management of gastroenteric neuroendocrine tumors. This technique uses an octreotide analog labeled with indium 111 (Octreoscan 111) administered intravenously (19). The radionucleotide identifies neuroendocrine tumors by binding to somatostatin receptors expressed by the tumors. SRS is capable of examining the entire body at one time, thereby detecting possible metastatic foci. The fact that up to 20% of neuroendocrine tumors, particularly insulinomas, do not express somatostatin receptors somewhat limits its applicability (26). This is most pronounced for insulinomas where up to 40% of tumors will not express somatostatin receptors and cannot be visualized by SRS (14, 15). Another limitation of scintigraphy is an inability to distinguish disease presence in a peripancreatic lymph node *versus* in the pancreatic gland itself. These problems can be reduced, but not eliminated by the use of single-photon emission CT.

In a study of gastrinomas, Gibril *et al.* (9) compared SRS to CT, MRI, and transabdominal ultrasound and reported an overall sensitivity for SRS of 70% and 92% sensitivity for detecting hepatic metastases. The sensitivity for SRS was greater than all other imaging modalities combined but it still missed 20% of gastrinomas. This study did not evaluate the usefulness of EUS. In another study, the ability of SRS to detect duodenal wall and peripancreatic lymph node gastrinomas was compared to other imaging modalities including EUS (27). The investigators report low but identical sensitivities of 58% for both EUS and SRS and illustrate the difficulty of localizing duodenal gastrinomas. Combining these two modalities resulted in a sensitivity of 90% (17 of 19 patients) for tumor localization. Endoscopic ultrasound detected all tumors visualized by any other conventional technique (excluding SRS) calling into question the real usefulness of these other imaging procedures.

In a recent study by Lebhati *et al.* (12), somatostatin receptor scintigraphy for the detection of gastroenteropancreatic tumors was compared to other imaging techniques including EUS. One-half of the study population of 160 patients had Zollinger-Ellison syndrome, 38 had a carcinoid tumor, and 44 patients had other types of neuroendocrine tumors. Patients with insulinomas were not included. Only 21 patients in the group without known metastases underwent surgery, limiting an accurate comparison between SRS and EUS. Overall, conventional imaging including EUS was positive in 71% of 160 patients compared to 78% with positive SRS. Results of EUS alone were not reported. These researchers note a clear influence of tumor size on detection rates and report a sensitivity of only 38% for resected tumors that were $<$10 mm in size. This factor has a much less pronounced effect on EUS detection rates.

In summary, report of the largest single-center experience to date demonstrates the impressive success of EUS in accurately localizing pancreatic neuroendocrine tumors and directing their surgical management. Our analysis reveals a high sensitivity and specificity for EUS in the detection of pancreatic neuroendocrine tumors. Currently, endoscopic ultrasonography plays a primary and central role at our center in localizing these tumors preoperatively (18). The accuracy of EUS is in part related to endosonographer experience and undoubtedly contributes to our center's success with this technique. Extensive experience with pancreatic EUS ($\geq$100 cases/yr) is likely a key ingredient to the success seen in this study. We believe that frequent communication between the surgeon and the endosonographer is essential to develop expertise in evaluating these lesions. Further prospective studies comparing EUS to other imaging modalities, such as somatostatin receptor scinitigraphy and positron emission tomography scanning are needed. A prospective study comparing EUS with SRS is currently ongoing at our institution. Currently, approaches to preoperative localization of these tumors should be dictated by local preference and expertise, and ideally should include EUS if available.

Reprint requests and correspondence: James M. Scheiman, M.D., Director, Endoscopic Ultrasound Program, Division of Gastroenterology, Taubman Center Box 0362, University of Michigan Medical Center, Ann Arbor, MI 48109.
Received Nov. 10, 1999; accepted Apr. 27, 2000.

REFERENCES

1. Jensen RT, Norton JA. Endocrine neoplasms of the pancreas. In: Yamada T, ed. Textbook of gastroenterology, vol 2, 2nd ed. Philadelphia: JB Lippincott, 1995:2131–60.
2. Norton JA, Doppman JL, Cohen MJ, et al. Prospective study of gastrinoma localization and resection in patients with Zollinger-Ellison syndrome. Ann Surg 1986;204:468–79.
3. Sheppard BC, Norton JA, Doppman JL, et al. Management of islet cell tumors in patients with multiple endocrine neoplasia: A prospective study. Surgery 1989;106:1108–18.
4. Maton PN, Gradner JD, Jensen RT. Diagnosis and management of Zollinger-Ellison syndrome. Endocrinol Metab Clin North Am 1989;18:519.
5. Zimmer T, Ziegler K, Bader M, et al. Localisation of neuroendocrine tumours of the upper gastrointestinal tract. Gut 1994;35:471–5.
6. Galiber AK, Reading CC, Charboneau JW, et al. Localisation of pancreatic insulinoma:comparison of pre-and intraoperative US with CT and angiography. Radiology 1988;166:405–8.
7. Doppman JL, Chang R, Fraker DL, et al. Localization of insulinomas to regions of the pancreas by intra-arterial stimulation with calcium. Ann Intern Med 1995;123:269–73.
8. Gibril F, Doppman JL, Chang R, et al. Metastaticgastrinomas. localization with selective arterial injection of secretin. Radiology 1996;198:77–84.
9. Gibril F, Reynolds JC, Doppman JL, et al. Somatostatin receptor scintigraphy: Its sensitivity compared with that of other imaging methods in detecting primary and metastatic gastrinomas. Ann Intern Med 1996;125:26–34.
10. Modlin IM, Cornelius E, Lawton GP. Use of an isotopic somatostatin receptor probe to image: Gut endocrine tumors. Arch Surg 1995;130:367–74.
11. Bader M, Koppenhagen K. Somatostatin receptor scintigraphy in the diagnosis of neuroendocrine gastroenteropancreatic tumors. Recent Results Cancer Res 1996;142:137–62.

12. Lebtahi R, Cadiot G, Sarda L, et al. Clinical impact of somatostatin receptor scintigraphy in the management of patients with neuroendocrine gastroenteropancreatic tumors. J Nucl Med 1997;38:853–8.
13. Corleto VD, Scopinaro F, Angeletti S, et al. Somatostatin receptor localization of pancreatic endocrine tumors. World J Surg 1996;20:241–4.
14. Modlin IM, Tang LH. Approaches to the diagnosis of gut neuroendocrine tumors: The last word (Today). Gastroenterology 1997;112:583–90.
15. Kvols LK. Somatostatin-receptor imaging of human malignancies: A new era in the localization staging, and treatment of tumors. Gastroenterology 1993;105:1909–14.
16. Rosch T, Lightdale CJ, Botet JF, et al. Localization of pancreatic endocrine tumors by endoscopic ultrasonography. N Engl J Med 1992;326:1721–6.
17. Grant CS, van Heerden J, Charboneau JW, et al. Insulinoma. The value of intraoperative ultrasonography. Arch Surg 1988; 123:843–8.
18. Bansal R, Tierney W, Carpenter S, et al. Cost-effectiveness of endoscopic ultrasound for preoperative localization of pancreatic neuroendocrine tumors. Gastrointestinal Endoscopy 1999; 49:19–25.
19. Krenning EP, Kooij PPM, Pauwels S, et al. Somatostatin receptor. Scintigraphy and radionuclide therapy. Digestion 1996;57(suppl):57–61.
20. Pasieka JL, McLeod MK, Thompson NW, et al. Surgical approach to insulinomas: Assessing the need for preoperative localization. Arch Surg 1992;127:442–7.
21. Bottger TC, Junginger T. Is preoperative radiographic localization of islet cell tumors in patients with insulinoma necessary? World J Surg 1993;17:427–32.
22. Thompson NW, Czako PF, Fritts LL, et al. Role of endoscopic ultrasonography in the localization of insulinomas and gastrinomas. Surgery 1994;116:1131–8.
23. Ruszniewski P, Amouyal P, Amouyal G, et al. Localization of gastrinomas by endoscopic ultrasonography in patients with Zollinger-Ellison syndrome. Surgery 1995;117:629–35.
24. Lightdale CJ, Botet JF, Woodruff JM, et al. Localization of endocrine tumors of the pancreas with endoscopic ultrasonography. Cancer 1991;68:1815–20.
25. Bolondi L, Li Bassi S, Gaiani S, et al. Diagnosis of islet cell tumor by means of endoscopic ultrasonography. J Clin Gastroenterol 1990;12:218–21.
26. Reubi JC, Kvols LK, Waser B, et al. Detection of somatostatin receptors in surgical and percutaneous needle biopsy samples of carcinoids and islet cell carcinomas. Cancer Res 1990;50: 5969–77.
27. Cadiot G, Lebtahi R, Sarda L, et al. Preoperative detection of duodenal gastrinomas and peripancreatic lymph nodes by somatostatin receptor scintigraphy. Gastroenterology 1996;111: 845–54.
