# Supplementary material for: Diagnostic value of endoscopic ultrasound for insulinoma localization: A systematic review and meta-analysis
Source: PLoS One. 2018 Oct 23;13(10):e0206099. doi: 10.1371/journal.pone.0206099 (PMC6198953; doi:10.1371/journal.pone.0206099)
Supplement: S2 File — (ZIP) [file pone.0206099.s002.zip › included studies data availability EUS/EUS-guided FNA in the diagnosis of pancreatic.pdf]

EUS-guided FNA in the diagnosis of pancreatic neuroendocrine tumors before surgery

José Celso Ardengh, MD, Gustavo Andrade de Paulo, MD, Angelo Paulo Ferrari, MD

São Paulo, Brazil

Background: **The use of EUS for precise preoperative evaluation of pancreatic neuroendocrine tumors is well established; up to 80% of insulinomas can be localized. However, the EUS appearance of pancreatic neuroendocrine tumors can be similar to that of benign peripancreatic lymph nodes. The aim of this study was to evaluate the role of EUS-guided FNA in this setting.**

Methods: **Thirty patients (18 women, 12 men) with 33 pancreatic/peripancreatic lesions confirmed by surgery underwent EUS-guided FNA between February 1997 and September 2002. Transabdominal US and CT were obtained in all patients before EUS. The diagnosis of pancreatic neuroendocrine tumor was established based on morphologic appearance and immunohistochemical staining of cytologic and surgical specimens.**

Results: **EUS detected 32 of the 33 (96.9%) lesions (mean diameter 20 mm, range 5-97 mm). There was one complication (abdominal pain). For the 30 patients, the following diagnoses were made: functioning pancreatic neuroendocrine tumor (16 patients), non-functioning pancreatic neuroendocrine tumor (7), peripancreatic lymph node (5), inflammatory intrapancreatic nodule (1), and peripancreatic splenosis (1). Sensitivity, specificity, positive and negative predictive values, and accuracy of EUS-guided FNA were 82.6%, 85.7%, 95%, 60%, and 83.3%, respectively. There was one false-positive diagnosis by EUS-guided FNA and 4 false-negative diagnoses. In two of the latter cases, EUS-guided FNA was unsuccessful.**

Conclusions: **EUS-guided FNA is accurate and safe for the diagnosis of pancreatic neuroendocrine tumor and may have a role in determining management strategy. (Gastrointest Endosc 2004;60: 378-84.)**

Pancreatic neuroendocrine tumors (PNT) have a broad and often misleading clinical spectrum because of the production and the release of various hormones. Currently available laboratory techniques can identify the precise nature of a PNT in most cases: insulinoma, gastrinoma, somatostatinoma, glucagonoma, and carcinoid tumor. Diagnosis depends on clinical symptoms and identification of the hormone produced. However, between 15% and 30% of PNTs are non-functioning.[1] Patients with non-functioning PNTs may be asymptomatic, with the tumor being found by chance, or they may have symptoms because of a mass effect, including abdominal pain and jaundice.[1] The overall prevalence of functioning PNTs is low, approximately 10 per 1 million population. In contrast, the prevalence of PNTs in autopsy studies is higher (0.5%-1.5%). The annual incidence of PNTs is reported to be 1 to 4 per 1 million.[2]

Precise localization of PNTs is of utmost importance, because surgical resection is the only curative treatment.[3-5] The ability of standard imaging studies such as transcutaneous US, CT, and magnetic resonance imaging (MRI) to localize a PNT depends on the size of the tumor. CT and MRI localize fewer than 10% of PNTs less than 1 cm in diameter, 30% to 40% of PNTs 1 to 3 cm in size, and more than 50% of those more than 3 cm in diameter.[1,6-10] Of the standard imaging studies, selective abdominal angiography is among the most sensitive for localization of the primary tumor; it identifies 60% of small PNTs, such as insulinomas, and 73% of other PNTs.[6] However, it is a relatively expensive and invasive procedure that has a significant potential for complications.[11-14] Somatostatin receptor scintigraphy (SRS) can detect up to 90% of PNTs.[1] However, gastrinomas in the duodenal wall and insulinomas are not identified by this technique (sensitivity approximately 60%).[1] Up to 90% of insulinomas are identified by intra-operative US,[12,15] but this is technically difficult, and injury to the splenic vein because of pancreatic mobilization is a possible complication.[11]

EUS is highly accurate for pre-operative localization of PNTs, mainly insulinomas, and is a good

Received October 6, 2003. For revision January 25, 2004. Accepted March 19, 2004.

Current affiliation: EUS Section, Endoscopy Unit, Department of Gastroenterology, Universidade Federal de São Paulo, (UNIFESP - EPM), São Paulo, Brazil.

Reprint requests: José Celso Ardengh, MD, Alameda dos Arapanés 881/110, Moema - São Paulo, SP - Brazil 04524-001.

Copyright © 2004 by the American Society for Gastrointestinal Endoscopy
0016-5107/$30.00

PII: S0016-5107(04)01807-3

alternative to other more invasive methods.[11,13,16-20] In the study by Rösch et al.,[13] EUS had a sensitivity of 82% and a specificity of 95% for localization of PNTs.[13] In a cohort study of asymptomatic patients with multiple endocrine neoplasia (MEN) type 1, screening EUS identified 82% of PNTs removed surgically before the development of significant biochemical test abnormalities.[21] At EUS, however, PNTs may resemble peripancreatic lymph nodes, and differentiation of these two entities can be difficult.[22] Cytologic and/or histopathologic evaluation of specimens obtained by EUS-guided FNA (EUS-FNA) can confirm the diagnosis, thereby minimizing the rate of false-positive results.[22,23] With the development of laparoscopic PNT enucleation, correct localization has become essential. Thus, documentation of the presence of multifocal disease by EUS and EUS-FNA mandates a change in the therapeutic approach.

The aim of the present study was to assess the value of EUS and EUS-FNA in the diagnosis of PNTs and the differentiation of PNTs from peripancreatic lymph nodes before surgery.

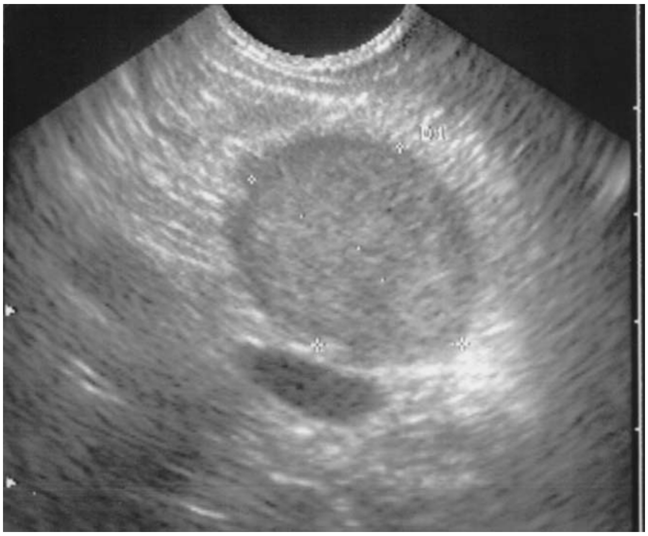

Figure 1. EUS image of well-demarcated, hypoechoic, regular, 2.5 × 2.0-cm nodule with posterior hyperechoic reinforcement. EUS-FNA confirmed the diagnosis of islet-cell tumor.

PATIENTS AND METHODS

A retrospective search of medical records identified 30 patients (18 women, 12 men; mean age 53.2, range 14-87 years) who underwent pre-operative EUS-FNA for suspected PNTs between February 1997 and September 2002. All patients but three had symptoms attributed to the lesion. Transabdominal US and CT were obtained in all patients before EUS, and 21 underwent MRI before EUS; most had imaging findings suggestive of PNT. All patients underwent surgery, and the results of EUS-FNA were compared with the final surgical diagnosis.

Twenty patients had hormonal disturbances, including hypoglycemia (12 patients), Zollinger-Ellison syndrome (5), carcinoid syndrome with high urinary 5-hydroxyindole acetic acid levels (2), and MEN type I (1). Seven had symptoms secondary to a tumor mass, including abdominal pain (5) and jaundice (2), and 3 were asymptomatic (pancreatic nodule found incidentally during check-up examination).

EUS was performed to localize the lesion and to obtain samples for cytologic and histopathologic diagnosis. All procedures were performed on an out-patient basis; patients remained in the endoscopy unit for 2 hours after EUS. Antibiotics were not administered prophylactically. EUS was performed by a single endoscopist (J.C.A.), who has over 10 years of experience (>6000 EUS procedures). All patients were sedated by intravenous administration of midazolam and propofol. Before EUS, an examination was performed with a standard forward-viewing endoscopy to search for lesions in the wall of the GI tract; a side-viewing endoscope was not used. Ulcer, thickened gastric folds, and/or other evidence of hypersecretion was not observed in any patient. EUS was performed with a linear echoendoscope (FG 32-UA, FG 36-UX, or FG 38-UX [Pentax Precision Instrument Corp., Orangeburg, N.Y.], with a Hitachi EUB 515 processor [Mitsubishi, Conshockon, Pa.] or a UCT 160 with Exera EU-C60 processor [Olympus America Corp., Melville, N.Y.]).

At EUS, a PNT was suspected if a regular, homogeneous lesion was found with well-demarcated margins and hyperechoic posterior reinforcement (caused by vascularization) (Fig. 1). A considerable proportion of the lesions were anechoic (Fig. 2). The entire pancreas and surrounding tissues were thoroughly examined for multifocal tumors.

After identification and staging of the lesion, EUS-FNA was performed by using 22-gauge needles (Hancke/Vilmann, Medizintechnik GmbH, Grassau, Germany [17 patients]; NA-10J-1 [3 patients], Olympus; NA-10J-KB [10 patients], Olympus) as follows: localization and alignment of the tumor with the axis of the needle; introduction of the needle (with stylet) into the tumor, under EUS guidance; removal of the stylet and confirmation that the needle tip was within the lesion, application of negative pressure with a 20-mL syringe; and to-and-fro motions of the needle. EUS-FNA was repeated if visual examination revealed that the aspirate was insufficient for cytologic diagnosis. A cytopathologist was not present during the procedure to evaluate the specimens. The mean number of needle passes was 2 (range 1-4). Color Doppler US was routinely used to identify any blood vessel between the GI wall and the lesion. The aspirated material was placed in a 10% solution of formalin and was submitted for preparation of a cell block. The sample was centrifuged and placed in agarose; slides were prepared in a standard manner. Immunohistochemical staining, including chromogranin and synaptophysin stains, was used in all cases.

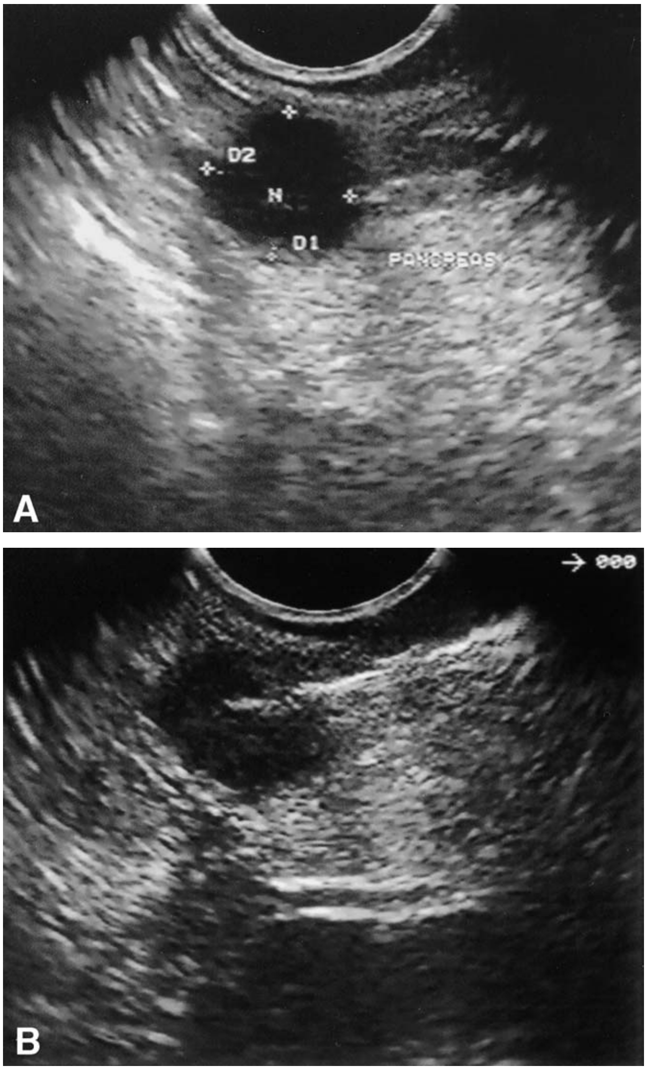

Figure 2. A, EUS image of anechoic, regular, 10.6 × 10.9-mm nodule. **B,** EUS-FNA of nodule.

Statistical analysis

The diagnosis by EUS-FNA was compared with the final diagnosis as determined by histopathologic evaluation of surgical resection specimens. Sensitivity, specificity, positive and negative predictive values, and accuracy were calculated. The 95% confidence interval was calculated by the exact binomial method.

RESULTS

Transcutaneous US identified lesions (mean diameter 3.26 cm) in 6 patients (20%). Dual-phase helical CT with intravenous contrast identified lesions in 18 patients (60%). In the 12 patients for whom CT was negative, mean lesion size was 1.0 cm. MRI depicted lesions in 6 of 21 patients (28.6%); mean lesion size was 2.75 cm. In 11 patients (36.6%), all imaging studies obtained before EUS were negative. Mean lesion size in these patients was 1.0 cm (Table 1).

At EUS, 28 patients had solitary lesions and two had multifocal lesions. Mean lesion size was 2.0 cm (range 0.5-9.7 cm). EUS revealed a homogenous, hypoechoic, well-demarcated, round nodule in 16 patients (53.3%); a homogeneous, isoechoic, round, well-demarcated nodule with posterior reinforcement in 8 patients (26.6%); a heterogeneous, hypoechoic mass with an irregular margin in 4 patients (13.3%); and a solid-cystic lesion in two patients (6.6%). Sixteen patients (53.3%) had a lesion in the body of the pancreas; in 10 (33.3%), the lesion was in the head; and, in 4 (13.3%), the lesion was in the tail. EUS-FNA was performed in 28 patients (93.3%). In two patients, it was not possible to insert the needle into the tumor; both had lesions in the body of the pancreas.

One patient had severe abdominal pain after EUS-FNA. He was hospitalized and discharged 24 hours later without pain.

Cytopathologic evaluation of the material obtained by EUS-FNA revealed an islet-cell tumor in 13 patients, insulinoma in 5, gastrinoma in one, and carcinoid in one. The aspirate was negative for PNT in 8 of the 28 patients.

All patients underwent surgery subsequent to EUS-FNA. In one patient, there was a mismatch between the number of lesions detected by EUS and the number found at surgical exploration. Enucleation was feasible in 17 patients. Seven patients underwent distal pancreatectomy, and 6 underwent pancreaticoduodenectomy (Whipple operation).

Final diagnoses, based on histopathologic evaluation of the resection specimens, were the following: functioning PNT (9 benign insulinoma, 3 malignant insulinoma, 2 gastrinoma, 1 carcinoid tumor, 1 somatostatinoma) (16/30 patients, 53.3%); nonfunctioning PNT (7/30 patients, 23.3%); benign peripancreatic lymph nodes/lymphoid tissue (5/30, 16.6%); inflammatory intrapancreatic nodule (1/30, 3.3%); and peripancreatic splenosis/nodule (1/30, 3.3%). The remaining 3 patients with Zollinger-Ellison syndrome had no PNT found at surgery and continue to have symptoms (Fig. 3).

In 5 patients, there was a discrepancy between the EUS-FNA findings and the assessment of the resection specimen. EUS in one patient with abdominal pain identified a well-defined nodule in the tail of the pancreas. Examination of the cell-block material obtained by EUS-FNA revealed an islet-cell tumor (immunohistochemical staining was not performed). At surgery, an intrapancreatic splenic nodule was found (Fig. 4). The EUS-FNA diagnosis, therefore, was false positive. In another patient, EUS-FNA of

Table 1. Clinical and tumor characteristics for 30 patients with suspected PNTs

Case no.	Age/gender	US/CT/MRI	EUS findings	Site/size (cm)	EUS-FNA	Surgery	Histology
1	49/F	+/+/NA	Solid cyst	Body/4.2	Islet cell	DP	MI*
2	63/F	-/+/+	Nodule	Body/1.0	Gastrinoma	DP	G
3	68/F	-/+/-	Nodule	Body/1.0	Negative	EN	LN
4	58/M	-/+/-	Nodule	Head/2.5	Islet cell	W	PNT
5	47/F	+/+/NA	Mass	Head/9.7	Negative	W	S
6	64/F	-/+/+	Mass	Head/2.5	Islet cell	W	PNT
7	58/M	-/+/-	Nodule	Body/1.6	Islet cell	DP	I
8	49/F	-/+/+	Nodule	Body/5.4	Insulinoma	DP	MI*
9	77/F	+/+/-	Nodule	Body/1.4	Iselt cell	EN	PNT
10	51/M	+/+/+	Mass	Body/3.1	Negative	EN	LN
11	57/M	-/+/-	Nodule	Tail/0.6	Islet cell	EN	PNT
12	48/M	-/+/-	Mass	Head/2.4	Insulinoma	W	I
13	58/M	-/+/-	Nodule	Head/2.9	Islet cell	W	PNT
14	35/M	-/+/-	Nodule	Head/1.6	Islet cell	EN	PNT
15	70/F	-/+/+	Nodule	Tail/1.5	Islet cell	DP	SPL
16	14/M	-/+/-	Mass	Tail/2.2	Insulinoma	DP	I
17	45/F	+/-/NA	Nodule	Body/1.1	Failure	EN	PNT
18	65/F	-/+/+	Solid cyst	Head/3.0	Carcinoid	W	C
19	78/F	+/+/-	Nodule	Body/1.0	Negative	EN	LN
20	23/F	-/-/NA	Nodule	Body/1.0	Negative	EN	I
21	62/F	-/-/-	Nodule	Body/0.7	Failure	EN	I
22	87/F	-/-/-	Nodule	Body/1.2	Negative	EN	LN
23	38/M	-/-/NA	2 nodules	Body/1.2 and 0.8	Negative	EN	PN
24	54/M	-/-/NA	Nodule	Head/1.9	Negative	EN	LN
25	36/F	-/-/-	Nodule	Head/1.2	Islet cell	EN	I
26	51/F	-/-/NA	Nodule	Tail/0.8	Islet cell	EN	MI*
27	36/F	-/-/-	Nodule	Body/1.6	Insulinoma	EN	I
28	55/F	-/-/-	Nodule	Head/1.0	Islet cell	EN	I
29	66/M	-/-/-	Nodule	Body/0.5	Islet cell	EN	G
30	36/M	-/-/NA	3 nodules	Body/0.8, 0.7, and 0.6	Insulinoma	DP	I

PNT, Pancreatic neuroendocrine tumor; *MRI*, magnetic resonance imaging; *EUS-FNA*, EUS-guided FNA; *NA*, not available; *DP*, distal pancreatectomy; *MI*, malignant insulinoma; *G*, gastrinoma; *EN*, enucleation; *LN*, lympdh node; *W*, whipple; *S*, somatostatinoma; *I*, insulinoma; *SPL*, splenosis; *C*, carcinoid; *PN*, pancreatitis nodule.

*An insulinoma was considered malignant when there was anaplasia, high mitotic index, and vascular invasion.

a mass in the head of the pancreas was negative for a PNT. However, examination of the resection specimen revealed a somatostatinoma. In the third case, insulinoma was suspected. EUS identified a nodule in the body of the pancreas, but EUS-FNA was negative for PNT. However, examination of the resection specimen confirmed the diagnosis of insulinoma. In two patients, EUS-FNA was not possible: in one, the nodule was too small; in the other, it was too hard and could not be punctured with the needle.

The diagnoses at surgery in these cases were insulinoma and non-functioning PNT.

The overall (30 patients) sensitivity, specificity, positive and negative predictive values, and accuracy of EUS-FNA for the diagnosis of PNT, as well as the 11 patients in whom a nodule was identified only by EUS, are listed in Table 2.

DISCUSSION

The diagnosis of PNT is based on the clinical presentation and the results of laboratory tests. Because these tumors are usually small, they are difficult to identify with standard imaging techniques such as transcutaneous US (sensitivity 15%) and CT (sensitivity 64%).[10,11,15] Only two studies found CT and angiography to be highly accurate, and the results in both were influenced by patient selection.[24,25]

Other imaging techniques are being used to identify PNTs, e.g., SRS, but success rates vary widely. Intra-arterial secretin injection with portal venous sampling was shown in one study to have high positive and negative predictive values for the identification of gastrinoma.[26] However, this procedure is highly invasive and does not provide a tissue diagnosis. Zimmer et al.[27] compared EUS and SRS in the localization of gastrinoma and insulinoma and found a higher accuracy for EUS (79% for gastrinoma, 93% for insulinoma).

The present series includes 33 lesions (mean diameter 2.0 cm) localized by EUS in 30 patients. Because no patient underwent total pancreatectomy,

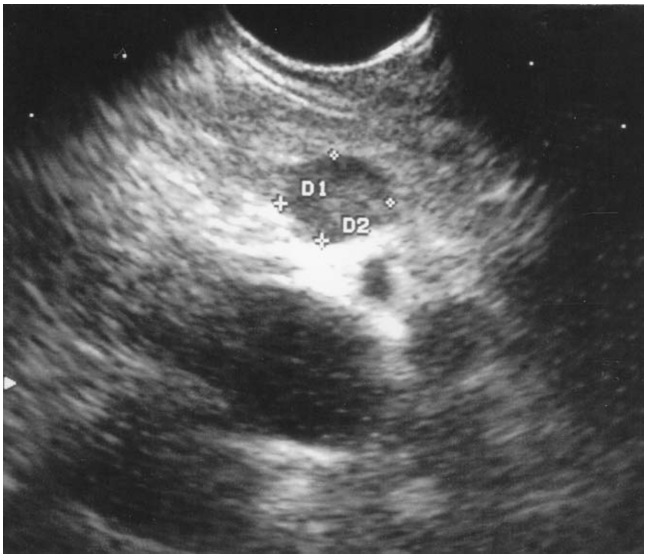

Figure 3. EUS image showing hypoechoic, regular, 7 × 10-mm nodule with well-defined margins and posterior reinforcement in body of pancreas in patient with Zollinger-Ellison syndrome. Transabdominal US and CT demonstrated a pancreatic nodule. EUS-FNA disclosed benign epithelial cells and many lymphocytes. Histopathologic examination of the enucleated nodule revealed a peripancreatic lymph node without malignancy.

other multifocal lesions might have been missed. The accuracy of transabdominal US, CT, and MRI was low, 20%, 60%, and 28.6%, respectively, for all lesions (functioning PNT, non-functioning PNT, peripancreatic lymph nodes). EUS can identify lesions as small as 2 to 3 mm in diameter within the pancreas or the wall of the GI tract. Moreover, EUS is cost effective when used early in the preoperative localization algorithm; it reduces the need for additional invasive tests, thereby decreasing morbidity, and it conserves resources.[18]

The major limitation of EUS is that it is operator-dependent; the greater the experience, the higher the probability of finding a tumor, especially lesions less than 2 cm in size. However, even experienced endosonographers have difficulty in differentiating pancreatic nodules and peripancreatic lymph nodes. In the present series, no EUS feature was identified that could be used to make this distinction. Experience is even more essential for EUS-FNA, a technique that enhances the positive predictive value of EUS. In the current study, only 76.6% of the patients suspected of having a PNT actually had such a tumor.

EUS-FNA is safe and effective, and enhances the ability to distinguish benign from malignant lesions.[28] However, the accuracy of EUS and EUS-FNA depends on the nature of the tumor; it is lower

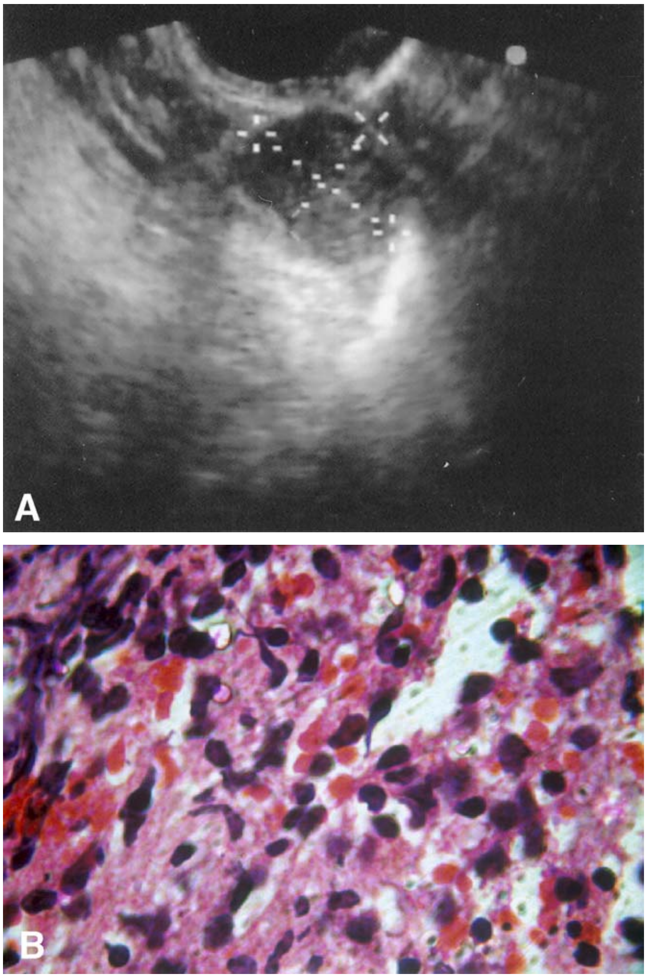

Figure 4. A, EUS image showing hypoechoic, regular, 15.1 × 11.7-mm nodule in tail of pancreas. **B,** Photomicrograph of resection specimen, showing intrapancreatic splenosis nodule; small, round blue cells are present in sinusoidal stroma (H&E, orig. mag. ×400).

Table 2. Sensitivity, specificity, accuracy, positive and negative predictive values of EUS-FNA in the overall diagnosis of PNTs (30 patients) and in 11 patients in whom only EUS found a nodule

EUS-FNA	n = 30	%	95% CI	n = 11	%	95% CI
Sensitivity	19/23	82.6	60.5, 94.3	6/8	75	35.6, 95.5
Specificity	6/7	85.7	42.0, 99.0	3/3	100	31.0, 100.0
Accuracy	25/30	83.3	64.5, 93.7	9/11	81.8	NA
PPV	19/20	95.0	73.1, 99.7	6/6	100	51.7, 100.0
NPV	6/10	60.0	27.9, 86.9	3/5	60	17.0, 92.7

EUS-FNA, EUS-guided FNA; *PNT*, pancreatic neuroendocrine tumor; *CI*, confidence interval; *NA*, not available; *PPV*, positive predictive value; *NPV*, negative predictive value.

for PNTs compared with pancreatic adenocarcinoma.[29] Several explanations for this lower accuracy are possible: cytologic samples obtained from PNTs tend to be more hemorrhagic, and it is technically

more difficult to obtain specimens from small lesions. However, when cytopathologic assessment is combined with immunohistochemistry, the diagnostic accuracy for PNTs is enhanced.[30,31] But even in this setting, a false-positive result is possible. One of our patients had EUS findings suggestive of PNT; EUS-FNA confirmed this diagnosis, but examination of the surgical resection specimen revealed a splenosis nodule (Fig. 4).

Curative surgical resection of PNTs relies on precise pre-operative localization. EUS allows identification of tiny lesions that are difficult to find by palpation during surgery. In one of our patients, gastrinoma was diagnosed by EUS-FNA, but the tumor could not be found at surgery. However, intraoperative US confirmed the presence of the lesion. To avoid this problem, small lesions are tattooed by us before surgery.[17] A determination as to whether a single lesion or multiple lesions are present is pivotal for surgical planning. In the present study, EUS identified multiple lesions in two cases. In the study of Ginès et al.,[22] a greater proportion of patients had multiple lesions.

Peripancreatic lymph nodes and splenosis nodules are small, regular lesions with variable echogenic patterns that can be confused at EUS with PNTs. This has serious implications, because 5 of our patients with these types of lesions underwent surgery based on a strong clinical suspicion for PNT. Although the EUS images were suggestive of PNT, cytologic evaluation of the aspirated specimens obtained by EUS-FNA revealed benign cells. Although EUS was highly specific (85.7%) for the diagnosis of PNT, the negative predictive value was 60%. Thus, EUS-FNA could not definitively exclude PNT.

To date, there is no study of EUS-FNA accuracy for small PNTs not identified by other imaging modalities. In the present study, there were 11 patients in whom US and CT were negative for PNT; MRI was negative in 6 and not available in 5. Mean tumor size in these 11 patients was 1.08 cm. In this subgroup of patients, EUS-FNA had a sensitivity of 75%, a specificity of 100%, a positive predictive value of 100%, a negative predictive value of 60%, and an accuracy of 81.8%, and it was superior to any of the other imaging modalities.

The patients in the present study may not be representative of the overall population of patients at risk for PNT. Many such patients undergo EUS without EUS-FNA; others have EUS-FNA but not surgery, and some undergo surgery without prior EUS. In our unit, all patients with a pancreatic nodule undergo EUS and EUS-FNA before any treatment.

EUS-FNA is safe and highly accurate for confirmation/exclusion of PNTs, and it enhances the overall results of EUS. If a malignant lesion is found before surgery, the choice of operation is optimized. If the result of EUS-FNA is negative, the lesion may be a benign peripancreatic lymph node. Unfortunately, because EUS-FNA cannot absolutely exclude a PNT, surgery may still be indicated if the EUS appearance is consistent with PNT and the level of clinical suspicion for PNT is high.

REFERENCES

1. Modlin IM, Tang LH. Approaches to the diagnosis of gut neuroendocrine tumors: the last word (today). Gastroenterology 1997;112:583-90.
2. Jensen RT, Norton JA. Pancreatic endocrine tumors. In: Feldman M, Scharschmidt BF, Sleisenger MH, editors. Sleisenger and Fordtran's gastrointestinal and liver disease: pathophysiology/diagnosis/management. 7th ed. Philadelphia: WB Saunders; 2002. p. 988-1016.
3. Broughan TA, Leslie JD, Soto JM, Hermann RE. Pancreatic islet cell tumors. Surgery 1986;99:671-8.
4. Fraker DL, Norton JA. The role of surgery in the management of islet cell tumors. Gastroenterol Clin North Am 1989; 18:805-30.
5. Azimuddin K, Chamberlain RS. The surgical management of pancreatic neuroendocrine tumors. Surg Clin North Am 2001; 81:511-25.
6. Jensen RT, Norton JA. Endocrine tumors of the pancreas. In: Feldman M, Scharschmidt BF, Sleisenger MH, editors. Sleisenger and Fordtran's gastrointestinal and liver disease: pathophysiology/diagnosis/management. 6th ed. Philadelphia: WB Saunders; 1998. p. 871.
7. Owen NJ, Sohaib SA, Peppercorn PD, Monson JP, Grossman AB, Besser GM, et al. MRI of pancreatic neuroendocrine tumours. Br J Radiol 2001;74:968-73.
8. Gunther RW, Klose KJ, Ruckert K, Kuhn FP, Beyer J, Klotter HJ, et al. Islet-cell tumors: detection of small lesions with computed tomography and ultrasound. Radiology 1983; 148:485-8.
9. Stark DD, Moss AA, Goldberg HI, Deveney CW. CT of pancreatic islet cell tumors. Radiology 1984;150:491-4.
10. Frucht H, Doppman JL, Norton JA, Miller DL, Dwyer AJ, Frank JA, et al. Gastrinomas: comparison of MR imaging with CT, angiography, and US. Radiology 1989;171:713-7.
11. Ardengh JC, Rosenbaum P, Ganc AJ, Goldenberg A, Lobo EJ, Malheiros CA, et al. Role of EUS in the preoperative localization of insulinomas compared with spiral CT. Gastrointest Endosc 2000;51:552-5.
12. Pitre J, Soubrane O, Palazzo L, Chapuis Y. Endoscopic ultrasonography for the preoperative localization of insulinomas. Pancreas 1996;13:55-60.
13. Rösch T, Lightdale CJ, Botet JF, Boyce GA, Sivak MV Jr, Yasuda K, et al. Localization of pancreatic endocrine tumors by endoscopic ultrasonography. N Engl J Med 1992;326: 1721-6.
14. Galiber AK, Reading CC, Charboneau JW, Sheedy PF 2nd, James EM, Gorman B, et al. Localization of pancreatic insulinoma: comparison of pre- and intraoperative US with CT and angiography. Radiology 1988;166:405-8.
15. King CM, Reznek RH, Dacie JE, Wass JA. Imaging islet cell tumours. Clin Radiol 1994;49:295-303.

16. Zimmer T, Scherubl H, Faiss S, Stolzel U, Riecken EO, Wiedenmann B. Endoscopic ultrasonography of neuroendocrine tumours. Digestion 2000;62(Suppl 1):45-50.
17. Gress FG, Barawi M, Kim D, Grendell JH. Preoperative localization of a neuroendocrine tumor of the pancreas with EUS-guided fine needle tattooing. Gastrointest Endosc 2002; 55:594-7.
18. Bansal R, Tierney W, Carpenter S, Thompson N, Scheiman JM. Cost effectiveness of EUS for preoperative localization of pancreatic endocrine tumors. Gastrointest Endosc 1999;49:19-25.
19. Hancke S. Localization of hormone-producing gastrointestinal tumours by ultrasonic scanning. Scand J Gastroenterol Suppl 1979;53:115-6.
20. Kann P, Bittinger F, Engelbach M, Bohner S, Weis A, Beyer J. Endosonography of insulin-secreting and clinically non-functioning neuroendocrine tumors of the pancreas: criteria for benignancy and malignancy. Eur J Med Res 2001;6:385-90.
21. Wamsteker EJ, Gauger PG, Thompson NW, Scheiman JM. EUS detection of pancreatic endocrine tumors in asymptomatic patients with type 1 multiple endocrine neoplasia. Gastrointest Endosc 2003;58:531-5.
22. Ginès A, Vazquez-Sequeiros E, Soria MT, Clain JE, Wiersema MJ. Usefulness of EUS-guided fine needle aspiration (EUS-FNA) in the diagnosis of functioning neuroendocrine tumors. Gastrointest Endosc 2002;56:291-6.
23. Santo E, Giovannini M. Role of endoscopic ultrasonography (EUS) and EUS guided fine needle aspiration in the evaluation of neuroendocrine tumors of the pancreas [abstract]. Digestion 1998;5:42.
24. van Heerden JA, Edis AJ, Service FJ. The surgical aspects of insulinomas. Ann Surg 1979;189:677-82.
25. Maton PN, Miller DL, Doppman JL, Collen MJ, Norton JA, Vinayek R, et al. Role of selective angiography in the management of patients with Zollinger-Ellison syndrome. Gastroenterology 1987;92:913-8.
26. Thom AK, Norton JA, Doppman JL, Miller DL, Chang R, Jensen RT. Prospective study of the use of intraarterial secretin injection and portal venous sampling to localize duodenal gastrinomas. Surgery 1992;112:1002-8; discussion 1008-9.
27. Zimmer T, Stolzel U, Bader M, Koppenhagen K, Hamm B, Buhr H, et al. Endoscopic ultrasonography and somatostatin receptor scintigraphy in the preoperative localisation of insulinomas and gastrinomas. Gut 1996;39:562-8.
28. Wiersema MJ, Vilmann P, Giovannini M, Chang KJ, Wiersema LM. Endosonography-guided fine-needle aspiration biopsy: diagnostic accuracy and complication assessment. Gastroenterology 1997;112:1087-95.
29. Voss M, Hammel P, Molas G, Palazzo L, Dancour A, O'Toole D, et al. Value of endoscopic ultrasound guided fine needle aspiration biopsy in the diagnosis of solid pancreatic masses. Gut 2000;46:244-9.
30. Yang GC, Slott S, LiVolsi VA, Gupta PK. Rapid assessment of Diff-Quik-stained pancreatic aspirates. A retrospective study of 40 intraoperative fine needle aspiration consultations, with measurement of nuclear size of look-alike small tissue fragments by image analysis. Acta Cytol 1994;38:37-42.
31. Prosser JM, Dusenbery D. Histocytologic diagnosis of neuroendocrine tumors in the liver: a retrospective study of 23 cases. Diagn Cytopathol 1997;16:383-91.

Moving?

To ensure continued service please notify us of a change of address at least 6 weeks before your move.

Phone Subscription Services at 800-654-2452 (outside the U.S. call 407-345-4000), fax your information to 407-363-9661, or e-mail elspcs@elsevier.com.
