# Supplementary material for: Diagnostic value of endoscopic ultrasound for insulinoma localization: A systematic review and meta-analysis
Source: PLoS One. 2018 Oct 23;13(10):e0206099. doi: 10.1371/journal.pone.0206099 (PMC6198953; doi:10.1371/journal.pone.0206099)
Supplement: S2 File — (ZIP) [file pone.0206099.s002.zip › included studies data availability EUS/localization of pancretic tumors by endoscoptic ultrasound.pdf]

The New England Journal of Medicine

©Copyright, 1992, by the Massachusetts Medical Society

Volume 326 JUNE 25, 1992 Number 26

LOCALIZATION OF PANCREATIC ENDOCRINE TUMORS BY ENDOSCOPIC ULTRASONOGRAPHY

THOMAS RÖSCH, M.D., CHARLES J. LIGHTDALE, M.D., JOSE F. BOTET, M.D., GREGORY A. BOYCE, M.D., MICHAEL V. SIVAK, JR., M.D., KENJIRO YASUDA, M.D., NORBERT HEYDER, M.D., LAURENT PALAZZO, M.D., HENRYK DANCYGIER, M.D., VOLKER SCHUSDZIARRA, M.D., AND MEINHARD CLASSEN, M.D.

Abstract *Background.* After a pancreatic endocrine tumor has been diagnosed on the basis of clinical signs and the results of laboratory tests, localization of the tumor by the usual imaging procedures fails in as many as 40 to 60 percent of patients. Endoscopic ultrasonography, a sensitive test for small carcinomas of the pancreas, might also be useful in patients with endocrine tumors of the pancreas that cannot be localized by conventional methods.

Methods. We studied 37 patients later shown to have 39 endocrine tumors of the pancreas who had negative results on transabdominal ultrasonography and CT. All the patients underwent endoscopic ultrasonography, and 22 also underwent selective angiography. All the tumors were confirmed by surgical excision and immunohistologic examination; they consisted of 31 insulinomas, 7 gastrinomas, and 1 glucagonoma, 0.5 to 2.5 cm (mean, 1.4 cm) in diameter. All but one of the patients were cured of their disease, as ascertained by at least six months of clinical and laboratory follow-up.

Results. Using endoscopic ultrasonography, we were able to localize 32 of the 39 tumors (sensitivity, 82 percent); no tumor was incorrectly localized. The size of the tumors was very similar (within 2 mm) to that predicted by endoscopic ultrasonography. Among the 22 patients who underwent both angiography and endoscopic ultrasonography, ultrasonography was significantly more sensitive than angiography for tumor localization (sensitivity, 82 percent vs. 27 percent). Among 19 control patients without pancreatic endocrine tumors, endoscopic ultrasonography was negative in 18 (specificity, 95 percent).

Conclusions. Endoscopic ultrasonography is a highly sensitive and specific procedure for the localization of pancreatic endocrine tumors. It should be considered for the preoperative localization of such tumors once the clinical and laboratory diagnosis has been established. (N Engl J Med 1992;326:1721-6.)

ONCE an endocrine tumor of potential pancreatic origin has been diagnosed on the basis of clinical signs and laboratory tests, surgery has to be performed in order to cure the disease, at least in patients who do not have metastases. Of the various endocrine tumors affecting the pancreas, insulinomas and gastrinomas are the most common. Insulinomas are located in the pancreas in 99 percent of cases,[1] whereas gastrinomas are located in extrapancreatic sites in 28 to 44 percent of cases,[2,3] most frequently in the duodenum, stomach, or lymph nodes.[3-6] Insulinomas are not localized by palpation at the time of surgery in up to 20 percent of cases,[7] but the failure rate can be reduced to 10 percent or less with intraoperative ultrasonography of the pancreas.[8,9] In up to 40 percent of patients with Zollinger–Ellison syndrome,[7] a gastrinoma is not found during surgery, possibly because of its extrapancreatic location in some cases.

In order to facilitate surgery, preoperative imaging procedures such as ultrasonography, CT, and angiography have been used to localize the primary tumor and detect metastases. Depending on their size, however, insulinomas and gastrinomas cannot be identified in 10 to 30 percent of patients.[3,6,10-26] These rather disappointing results have led to reliance on surgical exploration with the help of intraoperative ultrasonography, rather than on preoperative imaging procedures.[27] If the precise location of an endocrine tumor is known preoperatively, however, surgery is made considerably easier.

The introduction of endoscopic ultrasonography has allowed high-resolution imaging of the pancreas that can distinguish structures as small as 2 to 3 mm in diameter.[28] The accuracy of this procedure in diagnosing small pancreatic carcinomas has been reported to be close to 100 percent.[29,30] The method also seems to

From the Department of Internal Medicine II, Technical University of Munich, Munich, Germany (T.R., V.S., M.C.); the Memorial Sloan-Kettering Cancer Center, New York (C.J.L., J.F.B.); the Division of Digestive Diseases and Nutrition, College of Medicine, University of South Florida, Tampa (G.A.B.); the Department of Gastroenterology, Cleveland Clinic, Cleveland (M.V.S.); the Department of Gastroenterology, Kyoto Second Red Cross Hospital, Kyoto, Japan (K.Y.); the Department of Internal Medicine I, University Hospital Erlangen, Erlangen, Germany (N.H.); Conchon Gastroenterology Hospital, University of Paris V, Paris (L.P.); and the Department of Internal Medicine II, Municipal Hospital, Offenbach, Germany (H.D.). Address reprint requests to Dr. Rösch at the II. Medizinische Klinik und Poliklinik der Technischen Universität München, Klinikum rechts der Isar, Ismaningerstr. 22, 8000 München 80, Germany.

The New England Journal of Medicine
Downloaded from nejm.org at Philadelphia College of Osteopathic Medicine on October 5, 2017. For personal use only. No other uses without permission.
Copyright © 1992 Massachusetts Medical Society. All rights reserved.

be useful for the preoperative localization of small endocrine tumors of the pancreas, given the experience in small series.[31-34] We report here the collective experience at six centers where endoscopic ultrasonography has been used for the preoperative localization of small endocrine tumors of the pancreas. Only patients with normal results on transabdominal ultrasonography and CT were included.

Methods

Patients

We studied 17 men and 20 women, 22 to 75 years old (mean, 48), who had pancreatic endocrine tumors and in whom the results of preoperative transabdominal ultrasonography and CT were negative (with the exception of 1 patient with a gastrinoma who had hepatic metastases but negative results on imaging procedures to detect the primary pancreatic tumor). The patients were studied at six different centers from 1984 to 1991; none of the five patients at one center had undergone transabdominal ultrasonography. The diagnoses of insulinoma (29 patients), gastrinoma (7 patients), and glucagonoma (1 patient) were established on the basis of signs and symptoms and unequivocal results of laboratory tests before any imaging procedures were performed. The manifestations included hypoglycemia and elevated serum insulin and C-peptide concentrations occurring either spontaneously or, in the case of patients with insulinomas, during prolonged fasting; recurrent peptic ulcer disease plus elevated serum gastrin concentrations and, in doubtful cases of gastrinoma, a positive secretin test; and diabetes mellitus and necrolytic migratory erythema accompanied by an elevated serum pancreatic glucagon concentration in the patient with a glucagonoma.

All the patients later underwent surgery. The results of the endoscopic ultrasonography were not used in reaching the decision to operate. The endocrine tumors removed from the pancreas were measured, and portions were examined by routine histopathological and immunohistologic techniques. In one patient with a gastrinoma who was known preoperatively to have liver metastases, surgery was performed to reduce the size of the tumor. All the patients were followed for at least six months after surgery.

Nineteen patients in whom the possibility of a pancreatic endocrine tumor was at first suspected but eventually dismissed were also studied by endoscopic ultrasonography (11 men and 8 women, 17 to 75 years old [mean, 45]). All had symptoms suggesting the presence of an endocrine tumor of possible pancreatic origin, an elevated serum hormone concentration, or both. The presumptive diagnosis was insulinoma in nine patients, gastrinoma in eight, glucagonoma in one, and elevation of the serum pancreatic polypeptide level in one. In the patient with an elevated level of pancreatic polypeptide, the diagnosis of multiple endocrine neoplasia type 1 was suspected. In all 19 patients, further studies and follow-up did not confirm the presence of an endocrine tumor of the pancreas. The final diagnosis was either functional hypoglycemia (demonstrated by a negative fasting study and no further documented episodes of hypoglycemia) or elevated gastrin levels due to other causes (atrophic gastritis or H_2-antagonist drug treatment; the secretin test was negative in all the patients). All the patients were followed for at least six months. In the patient whose serum glucagon level was elevated, the value was normal on three subsequent occasions. The patient with an elevated serum pancreatic polypeptide concentration underwent laparotomy because endoscopic ultrasonography suggested multiple tumors in the pancreas.

Imaging Methods

Transabdominal ultrasonography was performed with modern real-time ultrasonic scanners, a 3.5-MHz linear-array scanner and a 5-MHz mechanical sector scanner. CT was performed with third-generation instruments; the thickness of the slice was 5 mm, and contrast material was administered orally and intravenously for better delineation of the pancreas. The rates of detection of pancreatic endocrine tumors by transabdominal ultrasonography and CT ranged from 30 percent to 50 percent at the various centers during the last five years of the study.

Endoscopic ultrasonography was performed with Olympus GF-UM2, GF-UM3, and JF-UM3 echoendoscopes. The technique has been described in detail elsewhere.[35] The echoendoscope is introduced into the descending duodenum, and starting at the level of the ampulla of Vater, the different parts of the pancreas are carefully scanned by slowly withdrawing the instrument. The head of the pancreas is visualized from the duodenum and the body and tail from the stomach. A water-filled balloon at the tip of the instrument is used to create the necessary fluid interface between the transducer and the gastrointestinal wall. In the stomach, additional water (200 to 600 ml) was sometimes necessary for this purpose. The procedure lasted between 10 and 45 minutes; as expertise was gained, the time became shorter at each center. Premedication, consisting of 5 to 15 mg of midazolam or diazepam, was usually administered.

The criteria used to evaluate endoscopic ultrasonography were its accuracy in localizing the tumor and in indicating its size, as compared with the measurements made after excision. The examiners were not aware of the operative and histopathological results. Photographs or videotapes from each examination were used to analyze the ultrasonographic features of the tumors.

Angiography was performed in 22 patients by selective catheterization of the celiac trunk and the superior mesenteric artery as well as the gastroduodenal artery, with special attention being paid to the pancreas. Generally, 60 to 90 ml of contrast medium was used. A presumptive diagnosis of endocrine tumor was made if a contrast blush was seen in either region of the pancreas. The angiography was performed after endoscopic ultrasonography in all but seven patients, who had negative results on angiography.

Results

Operative Findings and Follow-up

Thirty-nine endocrine tumors were found and removed from the 37 patients at the time of surgery. In all the patients the clinical classification of the tumor was confirmed by immunohistologic analysis. Among the 39 tumors, 20 were located in the head of the pancreas, 9 in the body, and 10 in the tail. Two patients each had two insulinomas. In one of them the tumors measured 0.5 and 1.7 cm in diameter and were in the pancreatic tail, and in the other the tumors measured 1.4 and 1.5 cm and were in the head of the pancreas. The mean diameter of all the tumors was 1.4 cm. The smallest lesion measured 0.5 cm and the largest 2.5 cm; 67 percent were 1.5 cm or less in diameter and 43 percent 1.0 cm or less (Table 1). Only two of the tumors, one a gastrinoma and the other an insulinoma, were malignant, as indicated by lymph-node metastases detected during surgery in both patients and by liver metastases diagnosed preoperatively by CT in the patient with the gastrinoma. The lymph-node metastases had been

Table 1. Diameter of 39 Endocrine Tumors in the 37 Study Patients, as Measured in the Resected Specimens.

Diameter (cm)	No. of Tumors
0.5–0.9	6
1.0	11
1.1–1.5	10
1.6–2.0	10
2.5	2

The New England Journal of Medicine
Downloaded from nejm.org at Philadelphia College of Osteopathic Medicine on October 5, 2017. For personal use only. No other uses without permission.
Copyright © 1992 Massachusetts Medical Society. All rights reserved.

detected by endoscopic ultrasonography in only one of the two patients.

All the patients were followed for at least six months. All but one were cured of their disease, as confirmed by the disappearance of symptoms and the normalization of laboratory-test results at six months. The patient with a malignant insulinoma was also asymptomatic eight months after surgery, but was then lost to follow-up. The patient with the malignant gastrinoma underwent tumor debulking (removal of the pancreatic tumor, lymphadenectomy, and partial hepatectomy). His serum gastrin concentration decreased by about 40 percent after surgery, but then began to increase four months later.

Results of Imaging Studies

Endoscopic ultrasonography correctly localized 32 of the 39 tumors (82 percent) (Table 2). Twenty-five of 31 insulinomas (in 23 of 29 patients) and 6 of the 7 gastrinomas, as well as 1 glucagonoma, were local-

Table 2. Sensitivity of Endoscopic Ultrasonography and Angiography in Detecting Pancreatic Endocrine Tumors Not Detected by Transabdominal Ultrasonography and CT.

Sensitivity of Endoscopic Ultrasonography in All Patients (N = 37)	Sensitivity in Patients Who Underwent Both Procedures (N = 22)	
	Endoscopic ultrasonography	Angiography
	tumors detected/all tumors (%)	
32/39 (82)	18/22 (82)	6/22 (27)

ized by endoscopic ultrasonography. In both patients with two tumors, both the tumors were correctly identified by endoscopic ultrasonography. Among the seven tumors (in seven patients) that were not found, one (diameter, 0.8 cm) was located in the head of the pancreas, two (1.0 cm each) in the body, and four (1.0, 1.2, 1.3, and 2.5 cm) in the tail. The reasons for failure differed, being related to location (pedunculated insulinomas adjacent to the body of the pancreas in one patient and adjacent to the tail of the pancreas in two patients), technical problems with the echoendoscope (in one patient), and insufficient visualization of the respective region of the pancreas (in two patients).

The size of the tumors as measured by endoscopic ultrasonography was close (within 2 mm) to that measured after surgical excision. The echo pattern of the tumors is shown in Table 3. Most were hypoechoic and clearly demarcated, but some were echo-rich or similar to the surrounding pancreatic tissue or had irregular margins (Fig. 1, 2, and 3.).

In the 22 patients who underwent angiography, a tumor, suggested by a blush in the respective region of the pancreas, was found in only 6 (27 percent) (Table 2). Endoscopic ultrasonography was positive in 18 of these 22 patients (82 percent) ($P<0.05$ by the chi-

Table 3. Ultrasonographic Features of the 32 Pancreatic Endocrine Tumors Detected by Endoscopic Ultrasonography.

Feature	No. of Tumors (%)
Ultrasonographic pattern*	
Homogeneous	26 (81)
Echo-poor	22
Similar to rest of pancreas	2
Echo-rich	2
Inhomogeneous	6 (19)
Cystic areas	3
Calcifications	2
Tumor margin	
Smooth	27 (84)
Irregular or indistinct	5 (16)
Echo-poor border	2 (6)

*As compared with the rest of the pancreas.

square test). Angiography, performed in four of the seven patients in whom endoscopic ultrasonography was negative, localized the tumor in only one of them. On the other hand, endoscopic ultrasonography detected the tumor in 13 of the 16 patients (81 percent) who had negative angiographic results; tumors were identified by endoscopic ultrasonography in 5 of the 6 patients in whom tumors were identified by angiography.

Endoscopic ultrasonography was correctly negative in 18 of the 19 control patients (specificity, 95 percent). In the patient with an increased serum polypeptide concentration in whom endoscopic ultrasonography was thought to show multiple tumors in the pancreas, the lesions proved at laparotomy to be small (0.8 to 1.2 cm) parapancreatic lymph nodes. No tumor of the pancreas was detected on careful surgical explo-

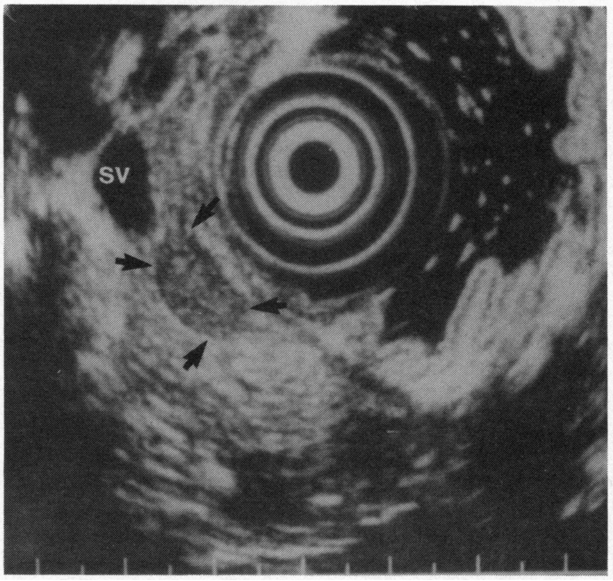

Figure 1. Ultrasound Image of an Insulinoma in the Body of the Pancreas.

Endoscopic ultrasonography from the stomach shows a hypoechoic mass (arrows). SV denotes splenic vein.

The New England Journal of Medicine
Downloaded from nejm.org at Philadelphia College of Osteopathic Medicine on October 5, 2017. For personal use only. No other uses without permission.
Copyright © 1992 Massachusetts Medical Society. All rights reserved.

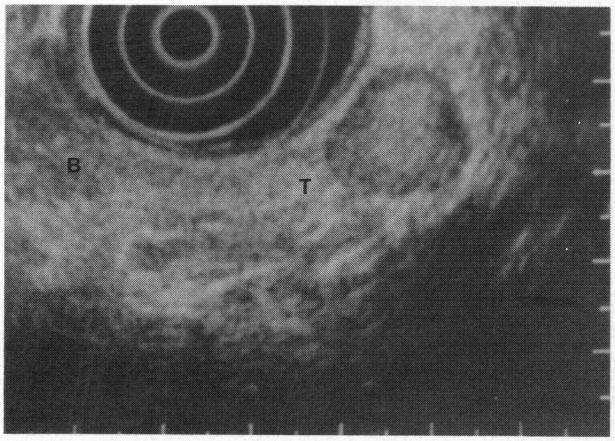

Figure 2. Ultrasound Image of a Gastrinoma in the Tail of the Pancreas.

Endoscopic ultrasonography of the tumor shows a central echo-rich area but a perimeter that is hypoechoic as compared with the rest of the pancreas. B denotes the pancreatic body, and T the pancreatic tail.

ration. Histologic examination of the resected lymph nodes did not reveal endocrine cells or malignant disease. The patient had no symptoms of a pancreatic endocrine tumor during the next six months.

Discussion

This study at six different centers was a retrospective analysis of data on patients with pancreatic endocrine tumors confirmed by surgery. A collaborative study such as this, although necessary to gather a sufficient number of cases of these rather rare tumors, has several disadvantages. The imaging procedures used at the various centers may not be uniform. The retrospective nature of the study might lead to a distortion of the results, especially of endoscopic ultrasonography, due to examiner bias. For this reason we excluded all patients who had positive findings on transabdominal ultrasonography or CT; the examiners were thus not aware of the location of the endocrine tumors within the pancreas, and the endoscopic ultrasonographic diagnoses were made before surgery.

We also excluded a few patients who were not operated on because they refused surgery despite clear biochemical and ultrasonographic evidence of a pancreatic tumor. We did so because we wanted to evaluate the accuracy of the procedure only in patients with surgically confirmed tumors. Our selection might therefore not be representative, but the patients included were those in whom preoperative localization of tumors was difficult. We found that endoscopic ultrasonography was highly sensitive in this subgroup of patients. The problem of specificity and false positive results can be addressed in two ways. In the case of pancreatic endocrine tumors, false positive results can mean incorrect localization of tumors or identification of a tumor in a patient who does not have one. In the 37 patients with 39 proved endocrine tumors, endoscopic ultrasonography localized 32 tumors correctly. In a further 19 patients without pancreatic endocrine tumors, the specificity was 95 percent. The high specificity of endoscopic ultrasonography is further substantiated by a recent study of endoscopic ultrasonography in the diagnosis of pancreatic cancer, in which there were no false positive results in 30 control patients.[30]

Detection of endocrine tumors that arise at extrapancreatic sites, most of which are gastrinomas, was not within the scope of this study. Tumors within the duodenal or gastric wall could probably also be detected, but ultrasonographic screening of the stomach and duodenum with submaximal balloon filling and water rinsing is time-consuming and probably not as effective as the visualization of the pancreas. In the literature, positive[33] and negative[34] results of the diagnosis of duodenal-wall gastrinomas by endoscopic ultrasonography have been reported. With transhepatic venous hormone sampling it is often not possible to differentiate reliably between duodenal-wall and pancreatic-head lesions, since both regions are drained through the same vessels. Endoscopic ultrasonography, on the other hand, is accurate in excluding a tumor in the head of the pancreas. However, neither method is sufficiently accurate in localizing gastrinomas at different sites in the duodenum. Intraoperative transduodenal endoscopic illumination, which was recently described in the localization of duodenal-wall gastrinomas,[36] might be more helpful in this respect.

Endoscopic ultrasonography detected approximately 80 percent of small endocrine tumors of the pancreas that were not detected by transabdominal ultrasonography or CT. This sensitivity level is probably a slight underestimate, since some of the failures were due to technical problems with the echoendoscope. This view is supported by the fact that two large tumors (2.0 and 2.5 cm), which are usually detected easily by endoscopic ultrasonography,[29,30] were overlooked in two patients in our series. The learning curve with endoscopic ultrasonography is steep, leading to substantially better results after 50 to 100 examinations. Our results also showed that even small tumors (1 cm or less) could be recognized reliably and that the accuracy rate was similar in all parts of the pancreas. On the other hand, it is possible that transabdominal ultrasonography and CT would have detected a few of these small tumors if the studies had been repeated with an improved technique (e.g., thinner slice or the spiral technique).

In most studies of the accuracy of transabdominal ultrasonography, CT, and angiography in the detection of insulinomas and gastrinomas, 40 to 60 percent of the tumors were not localized, irrespective of their size.[3,6,10-26] Only a few studies report somewhat higher rates of accuracy for CT and angiography.[21,26] These better results may be due at least in part to patient selection. We studied only patients who had negative results on transabdominal ultrasonography and CT.

Transhepatic venous sampling was reported to have 94 percent sensitivity and 97 percent specificity in

The New England Journal of Medicine
Downloaded from nejm.org at Philadelphia College of Osteopathic Medicine on October 5, 2017. For personal use only. No other uses without permission.
Copyright © 1992 Massachusetts Medical Society. All rights reserved.

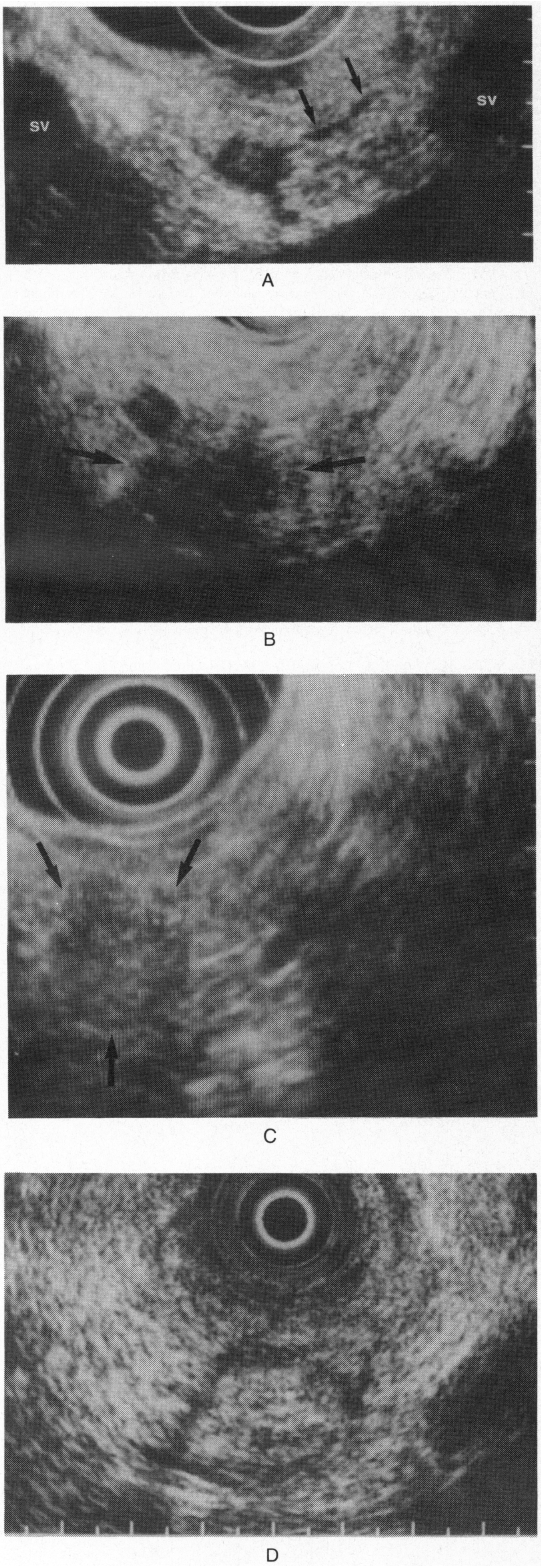

Figure 3. Aspects of Pancreatic Endocrine Tumors on Endoscopic Ultrasonography.

Panel A shows an insulinoma in the body of the pancreas that appears as a hypoechoic, smoothly demarcated tumor 5 mm in diameter (arrows indicate the pancreatic duct). SV denotes splenic vein. Panel B shows an insulinoma in the tail of the pancreas that has irregular or indistinct margins (arrows). Panel C shows a 1.2-cm insulinoma in the head of the pancreas (arrows) that is less well demarcated and almost isoechoic as compared with the rest of the pancreas. Panel D shows a glucagonoma with a hypoechoic tumor margin in the tail of the pancreas.

a recent study.[37] This, however, applied only to differentiation between the head of the pancreas and duodenum and the body and tail of the pancreas. More precise localization was not possible with this technique, and the correlation between the maximal hormone gradient and the location of the tumor was poor (sensitivity, 35 percent; specificity, 89 percent). Transhepatic venous sampling was not performed in any of the cases in our collective series, so a direct comparison of this technique with endoscopic ultrasonography cannot be made. Generally, venous sampling cannot distinguish between a tumor's location in the duodenal wall and one in the head of the pancreas. In such cases the clinical situation determines whether the duodenum and the head of the pancreas have to be removed surgically. In our opinion, venous sampling is more invasive, time-consuming, and arduous for the patient than endoscopic ultrasonography. No major complication of endoscopic ultrasonography has been reported, although the procedure has been used with increasing frequency for about 10 years. The rate of complications with transhepatic portography, which uses the same technique as transhepatic venous sampling, is in the range of 2 to 24 percent.[38,39]

In summary, we found that endoscopic ultrasonography is superior to angiography for the preoperative detection of small pancreatic tumors in patients in whom transabdominal ultrasonography and CT have not detected such tumors. It is therefore the most sensitive preoperative imaging method for the detection of small endocrine tumors of the pancreas. We suggest that endoscopic ultrasonography be used at an early stage in the localization of endocrine tumors of potential pancreatic origin, but only after the presence of such lesions has been virtually assured by laboratory testing.

References

1. Delvalle J, Yamada T. Secretory tumors of the pancreas. In: Sleisenger MH, Fordtran JS, eds. Gastrointestinal disease: pathophysiology, diagnosis, management. 4th ed. Philadelphia: W.B. Saunders, 1989:1884-900.
2. Ellison EH, Wilson SD. The Zollinger-Ellison syndrome: re-appraisal and evaluation of 260 registered cases. Ann Surg 1964;160:512-30.
3. Norton JA, Doppman JL, Collen MJ, et al. Prospective study of gastrinoma localization and resection in patients with Zollinger-Ellison syndrome. Ann Surg 1986;204:468-79.
4. Hofmann JW, Fox PS, Wilson SD. Duodenal wall tumors and the Zollinger-Ellison syndrome: surgical management. Arch Surg 1973;107:334-9.
5. Wolfe MM, Alexander RW, McGuigan JE. Extrapancreatic, extraintestinal gastrinoma: effective treatment by surgery. N Engl J Med 1982;306:1533-6.

The New England Journal of Medicine
Downloaded from nejm.org at Philadelphia College of Osteopathic Medicine on October 5, 2017. For personal use only. No other uses without permission.
Copyright © 1992 Massachusetts Medical Society. All rights reserved.

6. Wank SA, Doppman JL, Miller DL, et al. Prospective study of the ability of computed axial tomography to localize gastrinomas in patients with Zollinger-Ellison syndrome. Gastroenterology 1987;92:905-12.
7. Stefanini P, Carboni M, Patrassi N, Basoli A. Beta-islet cell tumors of the pancreas: results of a study on 1,067 cases. Surgery 1974;75:597-609.
8. Norton JA, Cromack DT, Shawker TH, et al. Intraoperative ultrasonographic localization of islet cell tumors: a prospective comparison to palpation. Ann Surg 1988;207:160-8.
9. Grant CS, van Heerden J, Charboneau JW, James EM, Reading CC. Insulinoma: the value of intraoperative ultrasonography. Arch Surg 1988;123:843-8.
10. Günther RW, Klose KJ, Rückert K, et al. Islet-cell tumors: detection of small lesions with computed tomography and ultrasound. Radiology 1983;148:485-8.
11. Galiber AK, Reading CC, Charboneau JW, et al. Localization of pancreatic insulinoma: comparison of pre- and intraoperative US with CT and angiography. Radiology 1988;166:405-8
12. Hemmingson A, Lindgren PG, Lörelius LE, Öberg K. Diagnosis of endocrine gastrointestinal tumours. Acta Radiol 1981;22:657-62.
13. Dunnick NR, Long JA Jr, Krudy A, Shawker TH, Doppman JL. Localizing insulinomas with combined radiographic methods. AJR Am J Roentgenol 1980;135:747-52.
14. Gorman B, Charboneau JW, James EM, et al. Benign pancreatic insulinoma: preoperative and intraoperative sonographic localization. AJR Am J Roentgenol 1986;147:929-34.
15. Pälvänsalo M, Mäkäräinen H, Siniluoto T, Stahlberg M, Jalovaara P. Ultrasound compared with computed tomography and pancreatic arteriography in the detection of endocrine tumours of the pancreas. Eur J Radiol 1989;9:173-8.
16. Proye C. Surgical strategy in insulinoma of adults: clinical review. Acta Chir Scand 1987;153:481-91.
17. Stark DD, Moss AA, Goldberg HI, Deveney CW. CT of pancreatic islet cell tumors. Radiology 1984;150:491-4.
18. Cho KJ, Vinik AI, Thompson NW, et al. Localization of the source of hyperinsulinism: percutaneous transhepatic portal and pancreatic vein catheterization with hormone assay. AJR Am J Roentgenol 1982;139:237-45.
19. Roche A, Raisonnier A, Gillon-Savouret MC. Pancreatic venous sampling and arteriography in localizing insulinomas and gastrinomas: procedure and results in 55 cases. Radiology 1982;145:621-7.
20. Giercksky KE, Halse J, Mathisen W, Gjone E, Flatmark A. Endocrine tumors of the pancreas. Scand J Gastroenterol 1980;15:129-35.
21. van Heerden JA, Edis AJ, Service FJ. The surgical aspects of insulinomas. Ann Surg 1979;189:677-82
22. Katz LB, Aufses AH Jr, Rayfield E, Mitty H. Preoperative localization and intraoperative glucose monitoring in the management of patients with pancreatic insulinoma. Surg Gynecol Obstet 1986;163:509-12.
23. Broughan TA, Leslie JD, Soto JM, Hermann RE. Pancreatic islet cell tumors. Surgery 1986;99:671-8.
24. Krudy AG, Doppman JL, Jensen RT, et al. Localization of islet cell tumors by dynamic CT: comparison with plain CT, arteriography, sonography, and venous sampling. AJR Am J Roentgenol 1984;143:585-9.
25. Hancke S. Localization of hormone-producing gastrointestinal tumours by ultrasonic scanning. Scand J Gastroenterol Suppl 1979;53:115-6.
26. Maton PN, Miller DL, Doppman JL, et al. Role of selective angiography in the management of patients with Zollinger-Ellison syndrome. Gastroenterology 1987;92:913-8.
27. Böttger TC, Weber W, Beyer J, Junginger T. Value of tumor localization in patients with insulinoma. World J Surg 1990;14:107-14.
28. Rösch T, Classen M. Endoscopic ultrasonography in pancreato-biliary disease. In: Herrlinger H, Megibow A, eds. Advances in gastrointestinal radiology. Vol. 2. Chicago: Mosby-Year Book (in press).
29. Yasuda K, Mukai H, Fujimoto S, Nakajima M, Kawai K. The diagnosis of pancreatic cancer by endoscopic ultrasonography. Gastrointest Endosc 1988;34:1-8.
30. Rösch T, Lorenz R, Braig C, et al. Endoscopic ultrasound in pancreatic tumor diagnosis. Gastrointest Endosc 1991;37:347-52.
31. Heyder N. Localization of an insulinoma by ultrasonic endoscopy. N Engl J Med 1985;312:860-1.
32. Bolondi L, Li Bassi S, Gaiani S, Campione O, Marrano D, Barbara L. Diagnosis of islet cell tumor by means of endoscopic ultrasonography. J Clin Gastroenterol 1990;12:218-21.
33. Lightdale CJ, Botet JF, Woodruff JM, Brennan MF. Localization of endocrine tumors of the pancreas with endoscopic ultrasonography. Cancer 1991;68:1815-20.
34. Ruszniewski P, Amouyal P, Combes R, et al. Endoscopic ultrasonography (EUS) is useful for the localization of primary gastrinomas (PG). Gastroenterology 1991;100:A297. abstract.
35. Rösch T, Braig C, Gain T, et al. Staging of pancreatic and ampullary carcinoma by endoscopic ultrasonography: comparison with conventional sonography, computed tomography, and angiography. Gastroenterology 1992;102:188-99.
36. Frucht H, Norton JA, London JF, et al. Detection of duodenal gastrinomas by operative endoscopic transillumination: a prospective study. Gastroenterology 1990;99:1622-7.
37. Vinik AI, Moattari AR, Cho K, Thompson N. Transhepatic portal vein catheterization for localization of sporadic and MEN gastrinomas: a ten-year experience. Surgery 1990;107:246-55.
38. Hoevels J, Lunderquist A, Owman T. Complications of percutaneous transhepatic catheterization of the portal vein and its tributaries. Acta Radiol 1980;21:593-601.
39. Viamonte M Jr, LePage J, Lunderquist A, et al. Selective catheterization of the portal vein and its tributaries: preliminary report. Radiology 1975;114:457-60.

The New England Journal of Medicine
Downloaded from nejm.org at Philadelphia College of Osteopathic Medicine on October 5, 2017. For personal use only. No other uses without permission.
Copyright © 1992 Massachusetts Medical Society. All rights reserved.
