# Supplementary material for: Diagnostic value of endoscopic ultrasound for insulinoma localization: A systematic review and meta-analysis
Source: PLoS One. 2018 Oct 23;13(10):e0206099. doi: 10.1371/journal.pone.0206099 (PMC6198953; doi:10.1371/journal.pone.0206099)
Supplement: S2 File — (ZIP) [file pone.0206099.s002.zip › included studies data availability EUS/MDCT vs endoscopic sonography comparison in preoperative localization of insulinomas.pdf]

(2):511
7 Oehrer PJ, Wick MR. Thymic malignancies. Cancer Treat Res,2001,105(2):277
8 Omiyama N, Muller NL, Ellis SJ, *et al*. Invasive and noninvasive thymoma: distinctive CT features. Comput Assist Tomogr,2001,25(3):388
9 Osado-de-Christenson ML, Galobardes , Moran CA. Thymoma: radiologic-pathologic correlation. Radiographics,1992,12(1):151
10 Rasmus JJ, McAdams HP, Donnelly LF, *et al*. MR imaging of mediastinal masses. MRI Clin North Am,2000,8(8): 59
11 Olina P, Siegel, Siegel MJ, Glazer HS. Thymic masses on MR imaging. AJR,1990,155(3): 495
12 Amera L, Brunetti A, Romano M, *et al*. Morphological imaging of thymic disorders. Ann Med, 1999,31(Suppl 2): 57

(2008-04-28 收稿 2009-03-16 修回)

多层螺旋CT与内镜超声对胰岛素瘤术前定位诊断的比较研究

薛华丹[1] 刘炜[1] 孙昊[1] 王萱[1] 陈钰[1] 金征宇[1] 杨爱明[2] 周炜洵[3]

【摘要】 **目的**: 比较多层螺旋CT与内镜超声对于胰岛素瘤术前定位诊断的临床价值。**材料和方法**: 15个月内低血糖症患者共35名(男:女=19:16, 平均年龄46.9岁),在2~7天内先后接受多层螺旋CT及内镜超声检查。两名放射科医师共同阅片做出CT诊断,两名消化内科医师共同做出内镜超声诊断。符合手术适应症的患者最终接受手术治疗并获得病理结果。将多层螺旋CT及内镜超声的诊断结果与病理结果进行对照分析。**结果**: 35名患者中共25名患者经术前评估行手术切除治疗,24名患者经病理确诊为胰岛素瘤(胰头钩突部8处,颈体部7处,尾部9处);术前多层螺旋CT检查共发现肿瘤24处(胰头钩突部9处,颈体部6处,尾部9处),术前内镜超声共发现肿瘤30处(胰头钩突部12处,颈体部9处,尾部9处)。以病理诊断为金标准、部位为统计单位,多层螺旋CT术前诊断胰岛素瘤的敏感度、特异度、阳性预测值、阴性预测值及准确度分别为95.8%(23/24)、100%(51/51)、100%(23/23)、98.1%(51/52)及98.7%(74/75),内镜超声的上述数值分别是79.2%(19/24)、86.3%(44/51)、73.1%(19/26)、89.8%(44/49)及84.0%(63/75);以临床诊断为金标准、病灶为统计单位,多层螺旋CT术前诊断胰岛素瘤的敏感度、特异度、阳性预测值、阴性预测值及准确度分别为95.8%(23/24)、98.8%(80/81)、95.8%(23/24)、98.8%(80/81)及98.1%(103/105),内镜超声分别是79.2%(19/24)、86.4%(70/81)、63.3%(19/30)、93.3%(70/75)及84.8%(89/105)。**结论**: 与内镜超声相比,多层螺旋CT有着更好的胰岛素瘤术前定位诊断价值。

关键词 胰岛素瘤;多层螺旋CT;内镜超声
中国图书资料分类法分类号 R 730.44

MDCT v. s. Endoscopic Sonography: Comparison in Preoperative Localization of Insulinomas

XUE Hua-dan, LIU Wei, SUN Hao, WANG Xuan, CHEN Yu, JIN Zheng-yu , YANG Ai-min, ZHOU Wei-xun (Department of Radiology, Peking Union Medical College Hospital, Beijing 100730)

[Abstract] **Purpoes**: To compare the value of MDCT and endoscopic sonography for preoperative detection of insulinomas. **Materials and Methods**: Patients (n=35, M:F=19:16, mean age=46.9) with suspicious insulinoma underwent MDCT (Somatom Sensation 64, Germany) and endoscopic sonography were included in this study over a 15 months period. Time interval between two examinations was 2 days to 1 week. CT scans were interpreted by two radiologists independently. Preoperative tumor localization capability of these two methods was analyzed. **Results**: Further clinical findings of ten patients showed neither typical laboratory results (GLU / INS < 0.3) nor typical clinical symptoms (negative result of starve experiment). Only 25 patients underwent operation after comprehensive preoperation evaluation, in which 24 had pathological diagnosis of insulinoma. Totally 24 lesions were found by MDCT scan and 30 lesions were found by endoscopic sonography examinations. Take pathology as the gold standard, sensitivity, specificity, positive predictive value, negative predictive value and accuracy of MDCT are 95.8%(23/24),100%(51/51),100%(23/23),98.1%(51/52) and 98.7%(74/75) by lesions, those value of endoscopic sonography are 79.2%(19/24)、86.3%(44/51)、73.1%(19/26)、89.8%(44/49) and 84.0%(63/75) by lesions. Take clinical diagnosis as the gold standard, sensitivity, specificity, positive predictive value, negative predictive value and accuracy of MDCT are 95.8%(23/24),98.8%(80/81),95.8%(23/24),98.8%(80/81) and 98.1%(103/105), those value of endoscopic sonography are 79.2%(19/24),86.4%(70/81),63.3%(19/30),93.3%(70/75) and 84.8%(89/105). **Conclusion**: Appropriate MDCT scanning pro-

作者单位 1. 100730 北京 协和医院放射科(通讯作者 金征宇) 2. 消化内科 3. 病理科

万方数据

tocol makes MDCT a relatively better method for preoperational revealing of insulinomas than endoscopic sonography.

Key words insulinoma; multislice CT; endoscopic sonography

胰岛素瘤是胰腺起源的最常见的神经内分泌肿瘤类型,手术切除是解除患者症状并治愈疾病的唯一方法。曾有部分学者认为一旦胰岛素瘤被定性诊断即无需进行术前的肿瘤定位[1,2],因为大部分肿瘤都位于胰腺实质内可通过术中观察、触诊及术中超声定位即可。但更多的学者肯定了术前定位诊断的重要性[3,4],一方面可以帮助确定手术方案,如朝向腹侧的胰体部外生型胰岛素瘤只需进行腹腔镜肿瘤剔除术,而深在胰腺实质内的内生型胰岛素瘤则可能需要行胰腺部分切除术;另一方面还可以显著缩短手术耗时,降低患者的手术风险。然而,胰岛素瘤术前定位诊断对于医学影像来说是一项较为艰巨的任务。几项影像学检查方法虽都可以用于胰岛素瘤的定位诊断,但多因敏感性较低、检查方法繁琐、昂贵不能令人满意。血管造影合并动脉血钙刺激肝静脉取样(Arterial Stimulation Venous Sampling, ASVS)的敏感性多在 50% 左右[5],但这是一种有创性检查,且操作复杂、禁忌症及术后并发症多。高场 MRI 检查的敏感性在 74% 到 85% 之间[6],但高场 MRI 设备昂贵、序列复杂且空间分辨率不够理想易造成漏诊。内镜超声及多层螺旋 CT 是近年来较为临床接受的两种不同的影像诊断手段,但二者的敏感度高低报道不一[7,8]。本研究旨在以临床定性诊断及手术病理结果为金标准,前瞻性的比较两种方法对于胰岛素瘤术前定位诊断的临床价值。

1 材料和方法

1.1 一般资料 2007-12 ~ 2009-02 前瞻性收集因低血糖症就诊的患者 34 例,男性 18 人,女性 16 人,年龄在 19 ~ 74 岁,平均年龄 46.9 岁。有症状出现至就诊间的时间间隔为 1.5 年至 12 年,平均间隔 5.8 年。均接受多层螺旋 CT 检查及内镜超声检查以除外胰岛素瘤的存在。两项检查间隔时间为 2 ~ 7 天。

1.2 仪器和方法

1.2.1 多层螺旋 CT

1.2.1.1 扫描方案 使用西门子 64 层螺旋 CT(Somatom Sensation64, Forchheim, Germany)。采用改良的多层螺旋 CT 胰腺双期增强扫描方案:扫描包括平扫期、动脉期及胰腺实质期扫描,各期扫描均为屏气扫描。其中,动脉期扫描参数为:管电流/管电压 160 mAs/120kv,螺距因子 1.5,准直 0.6mm × 64,扫描时间 3 ~ 4 秒,动脉期延迟启动扫描方式位于腹主动脉双肾动脉水平团注示踪,触发后 5s 启动(阈值 100Hu)。造影剂采用 350mgI 或 370mgI 非离子碘型造影剂(350mgI 欧乃派克 Omnipaque [iohexol], Nycomed, Oslo, Norway 或 370mgI 优维显 Ultravist [Iopromide], Bayer Schering Healthcare)70ml,注射速度 5ml/s,后追加 20ml 生理盐水冲洗锁骨下静脉。重建图像层厚/层间隔为 1.0mm/0.7mm,Kernel 值为 B30f。

1.2.1.2 图像分析及统计学评估 图像调入西门子工作站(Leonardo, Siemens AG Medical Solutions, Forchheim, Germany)中进行分析及诊断。两名腹部放射学专业放射诊断学医师分别读片决定诊断及测量结果并记录,之后进行讨论得到统一结论后作为最终的诊断结果。两名医师阅片时并不了解患者最终的病理诊断结果,但他们知道 CT 检查旨在发现胰岛素瘤。诊断标准为胰腺实质内发现动脉期和/或门脉期强化高于正常胰腺实质的病灶区。两位医师分别记录基于 CT 图像所得到的影像诊断结果及肿瘤部位。根据临床综合检查结果及手术病理结果计算多层螺旋 CT 对于胰岛素瘤术前定位诊断的准确度、敏感度、特异度、阳性预测值及阴性预测值。计算医师间最初诊断的一致性(kappa 值)。

1.3 内镜超声 采用 JF-UM20、GIF-UMQ 240 或 GF-UM2000(Olympus, Tokyo, Japan)内镜超声仪,频率调节范围为 5、7.5、12、20MHz。术前患者常规禁食 8 小时,局部麻醉,将内镜先端部送入十二指肠降部后逐渐退镜,于十二指肠内观察十二指肠、胰头、胰体及其周围结构,于胃体、胃底部观察胰体、胰尾及其周围结构。检查时用气囊接触法或腔内无气水充盈法。两位医师分别记录基于内镜超声所见的影像诊断结果及肿瘤部位,之后进行讨论得到统一结论后作为最终诊断结果。诊断标准为胰腺实质内发现低回声病灶。根据临床综合检查结果及手术病理结果计算内镜超声检查对于胰岛素瘤术前定位诊断的准确度、敏感度、特异度、阳性预测值及阴性预测值。计算医师间最初诊断的一致性(kappa 值)。

1.4 手术适应症及病理分析 满足下列情况之一的患者接受手术治疗:(1)经 CT 扫描或经内镜超声检查发现阳性病灶的患者;(2)经 CT 扫描或内镜超声检查未发现阳性病灶,但经进一步的磁共振检查、核素扫描/血管造影 + ASVS 检查进行定位诊断发现阳性病

万方数据

灶的患者;(3)经上述检查均未发现阳性病灶,但患者仍坚持手术探查者。另外接受手术的患者均不应有肝内转移灶的发现。术前有明确定位诊断的病例,根据患者肿瘤定位情况及其与血管、胰胆管系统的关系等选择术式(如腹腔镜或开腹手术)进行手术。术前无明确定位诊断的病例,采用术中对指触诊及术中超声的方法进行定位诊断,若发现病灶,则行切除或摘除。根据患者的手术结果记录术中肿瘤的大小、部位,并由内分泌肿瘤方面的专业病理医师审阅患者肿瘤HE染色及免疫组化病理切片,确定患者肿瘤的病理诊断。

2 结果

10名患者进一步的临床检查结果示GLU/INS < 0.3、饥饿试验阴性且没有典型的临床症状,其中临床诊断5例为反应性低血糖、2例药物性低血糖、1例多囊卵巢综合症、1例自身免疫性胰岛素综合症及1例癔症,最终未接受手术治疗。其余25名患者经完善的术前评估后接受手术治疗,1名患者术后病理诊断为胰岛细胞增生,24名患者病理诊断为胰岛素瘤,术中共发现并切除病灶24处(胰头钩突部8处,颈体部7处,尾部9处),病灶平均大小1.9 ±0.5cm。35名患者术前多层螺旋CT检查共发现肿瘤24处(胰头钩突部9处,颈体部6处,尾部9处),病灶平均大小1.8 ± 0.6cm,两位诊断医师间的诊断一致性为0.88。术前内镜超声共发现肿瘤30处(胰头钩突部12处,颈体部9处,尾部9处),病灶平均大小2.0 ±0.5cm,两位诊断医师间的诊断一致性为0.79(图1~3)。病理诊断为金标准、部位为统计单位,多层螺旋CT术前诊断胰岛素瘤的敏感度、特异度、阳性预测值、阴性预测值及准确度分别为95.8%(23/24)、100%(51/51)、100%(23/23)、98.1%(51/52)及98.7%(74/75),内镜超声的上述数值分别是79.2%(19/24)、86.3%(44/51)、73.1%(19/26)、89.8%(44/49)及84.0%(63/75);以临床诊断为金标准、病灶为统计单位,多层螺旋CT术前诊断胰岛素瘤的敏感度、特异度、阳性预测值、阴性预测值及准确度分别为95.8%(23/24)、98.8%(80/81)、95.8%(23/24)、98.8%(80/81)及98.1%(103/105),内镜超声分别是79.2%(19/24)、86.4%(70/81)、63.3%(19/30)、93.3%(70/75)及84.8%(89/105)。

3 讨论

胰岛素瘤是胰岛细胞瘤中最常见的病理类型。虽然有一定的复发率,但治愈手段仍然为手术切除。因此,胰岛素瘤的术前定位诊断是非常重要的。十年前,临床最常用的术前诊断方法是经皮经肝门脉取血、血管造影或动脉刺激静脉取血等有创性检查,但是这些方法的禁忌症和并发症较多且往往仍不能准确地进行肿瘤的三维立体定位,实际上对于手术来说仅起到了半定位的作用。目前在临床上包括MRI、血管造影、CT及超声内镜等很多影像学方法均被用于胰岛素瘤的术前定位。近期有研究表明内镜超声是胰岛细胞瘤最准确的诊断方法之一[9]。而高端多层螺旋CT扫描速度快,能够一次性采集多期三维数据,理论上能够有效地提高CT对胰岛细胞瘤定位诊断的敏感度。

3.1 CT与超声内镜对于胰岛素瘤的术前定位的比较

3.1.1 CT 关于超声内镜与CT对于胰岛素瘤的术前定位比较的文献结果差异较大,这很可能与CT设备的不断更新及文献作者的专业经验有关。64层螺旋CT的准直可达到0.6mm,图像空间分辨率也较既往的多层螺旋CT有了很大的提高,且扫描速度快、能够在3~4秒内完成上腹部的三维数据采集,这都为胰岛素瘤的定位诊断提供了非常重要的提升空间。因为胰岛素瘤体积多较小,临床确诊时常在2cm以下,如

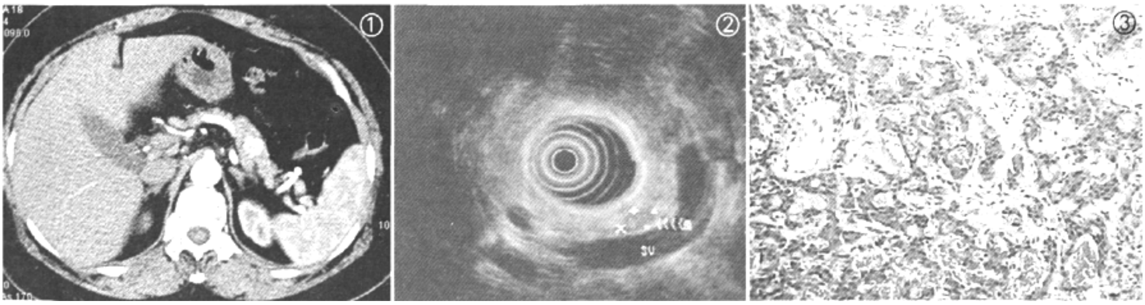

图1~3 胰体部胰岛素瘤的多层螺旋CT表现(图1)、超声内镜表现(图2)及病理HE染色切片(图3,150×)。CT动脉期病灶较正常胰腺实质有更明显的强化,超声内镜病灶呈稍低回声,形态呈类圆形,边界均较清晰。

万方数据

不采用三维容积观察方法则极易与胰周血管断面混淆或因部分容积效应造成漏诊或造成误诊。单层螺旋 CT 的敏感度为 63.3%(19/30),图像层厚 2.5 ~ 5mm[10],4 层螺旋 CT 敏感度提高到了 84.2%,图像层厚为 3.75mm [11],针对 64 层螺旋 CT 对胰岛素瘤定位诊断的准确度及敏感度的文献尚未见有统计学结论性的报道。本研究采用 64 层螺旋 CT 层厚为 1.0mm 的图像,将敏感度进一步提高到了 95.8%。当然,胰岛素瘤的强化特征也是多种多样的,有极少部分胰岛素瘤的强化特征为等强化甚至低强化[12],其中等强化者显然会是造成多层螺旋 CT 漏诊的重要原因之一。因此多层螺旋 CT 显然不是唯一有效的胰岛素瘤影像诊断手段,对于某些特殊的病例尚需包括超声内镜在内的多种影像手段的相互补充和验证。

3.1.2 超声内镜　超声内镜(EUS) 因探头可以放置于与胰腺紧邻的胃和十二指肠,探头距胰腺表面的距离不超过 1.0 cm,减少了胃肠道气体及其它软组织对胰腺检查的遮挡和干扰,提高了胰腺成像的清晰度,使胰腺小病变的检出成为可能。内镜超声对胰腺内分泌肿瘤的定位准确性较高[13,14],但超声内镜的患者耐受性相对较差,检查禁忌症相对较多,且一些研究发现内镜超声有较为显著的操作者依赖性,可能对定位的准确性产生明显的副作用[15]。此外,胰岛细胞瘤在超声内镜下的特点也不尽相同,虽大部分表现为低回声为主的特征,但也有部分呈现中等回声或中等偏强回声者,需要操作者及诊断医师有较好的临床经验。由于胰腺与胃壁的位置关系的不一致性,内镜超声对于胰头及胰体部的病灶更容易发现,而胰尾部则容易出现漏诊[16]。本研究的结果也支持上述观点,但主要表现在胰头及胰体部发现了更多的假阳性病灶。另外,近年来有学者认为超声内镜与 CT 的结合能够达到接近 100% 的胰岛素瘤术前定位能力,CT 做为一线检查手段,超声内镜则适合对有疑问或 CT 没能准确定位的病例进行进一步的定位诊断[17,7],这种综合诊断的方法不失为一种很好的临床实用性强的思路。

3.2 本研究的局限性　本研究的局限性在于样本量较小,尚需要大样本多中心的验证;另外对于临床诊断为阴性的患者没有进一步进行病理诊断,因此实际上其中 4 例内镜超声发现的病灶无法证实其真实性。此外,对于多层螺旋 CT 及超声发现小胰岛素瘤(<1cm)的能力比较有待于进一步评估。

总之,本研究表明内镜超声的假阳性率及假阴性率均相对较高,且操作者依赖性较强,而多层螺旋 CT 有着更高的敏感性和特异性,且患者痛苦小、结果客观性较好,操作者间一致性较高,更适合于作为胰岛素瘤术前定位诊断的影像学方法。

参 考 文 献

1 Boukhman MP, Karam JM, Shaver J, *et al.* Localization of insulinomas. Arch Surg ,1999,134(8):818
2 Hashimoto LA, Wash RM. Preoperative localization of insulinomas is not necessary. J Am Coll Surg ,1999,189(4):368
3 Kuzin NM, Egorov AV, Kondrashin SA, *et al.* Preoperative and intraoperative topographic diagnosis of insulinomas. World J Surg ,1998,22(6):593
4 Ravi K, Britton BJ. Surgical approach to insulinomas: are pre-operative localization tests necessary? Ann R Coll Surg Engl ,2007,89(3):212
5 Vinik AI, Delbridge L, Moattari R, *et al.* Transhepatic portal vein catheterization for localization of insulinomas: a ten-year experience. Surgery, 1991,109(1):1
6 Thoeni RF, Mueller-Lisse UG, Chan R, *et al.* Detection of small, functional islet cell tumors in the pancreas: selection of MR imaging sequences for optimal sensitivity. Radiology, 2000,214(2):483
7 Herve G, Olivier V, Joelle A, *et al.* CT, Endoscopic Sonography, and a combined protocol for preoperative evaluation of pancreatic insulinomas. AJR, 2003,181(4):987
8 杨爱明,陆星华,钱家鸣,等. 超声内镜在胰岛细胞瘤定位诊断中的作用. 中华消化内镜杂志,2006,23(3):169
9 Nesje LB, Varhaug JE, Husebye ES, *et al.* Endoscopic ultrasonography for preoperative diagnosis and localization of insulinomas. Scand J Gastroenterol, 2002,37(6) :732
10 Fidler JL, Fletcher JG, Reading CC, *et al.* Preoperative detection of pancreatic insulinomas on multiphasic helical CT. AJR Am J Roentgenol, 2003,181(3):775
11 Rappeport ED, Hansen CP, Kjaer A, *et al.* Multidetector computed tomography and neuroendocrine pancreaticoduodenal tumors. Acta Radiol,2006,47(3):248
12 Sheth S, Hruban RK, Fishman EK. Helical CT of islet cell tumours of the pancreas: typical and atypical manifestations. AJR Am J Roentgenol, 2002,179(3):725
13 Pitre J, Soubrane O, Palazzo L, *et al.* Endoscopic ultrasonography for the preoperative localization of insulinomas. Pancreas, 1996,13(1):55
14 Varas Lorenzo MJ, Miquel Collell JM, Maluenda Colomer, *et al.* Preoperative detection of gastrointestinal neuroendocrine tumors using endoscopic ultrasonography. Rev Esp Enferm Dig, 2006,98(11):828
15 Schumacher B, Lübke HJ, Frieling T, *et al.* Prospective study on the detection of insulinomas by endoscopic ultrasonography. Endoscopy, 1996,28(3):273
16 Buscail L. Endoscopic ultrasonography in pancreatobiliary disease using radial instruments. Gastrointest Endosc Clin N Am, 1995,5(4):781
17 Alison McLean. Endoscopic ultrasound in the detection of pancreatic islet cell tumours. Cancer Imaging, 2004,4(2):84

(2009-04-15 收稿　2009-05-26 修回)

万方数据
