# Supplementary material for: Diagnostic value of endoscopic ultrasound for insulinoma localization: A systematic review and meta-analysis
Source: PLoS One. 2018 Oct 23;13(10):e0206099. doi: 10.1371/journal.pone.0206099 (PMC6198953; doi:10.1371/journal.pone.0206099)
Supplement: S2 File — (ZIP) [file pone.0206099.s002.zip › included studies data availability EUS/Noninvasive imaging of insulinomas.pdf]

Noninvasive imaging of insulinomas and gastrinomas with endoscopic ultrasonography and somatostatin receptor scintigraphy

Charles Proye, MD, FRCS(Edin)(Hon), Philippe Malvaux, MD, François Pattou, MD, Bernard Filoche, MD, Jean-Michel Godchaux, MD, Vincent Maunoury, MD, Laurent Palazzo, MD, Damien Huglo, MD, Jean Lefebvre, MD, *and* **Jean-Claude Paris, MD,** *Lille, Lomme, and Paris, France*

Background. *Classic morphological techniques are of limited value for imaging endocrine duodenopancreatic tumors, and invasive procedures such as intraarterial stimulation are often used. Two noninvasive procedures, endoscopic ultrasonography (EUS) and somatostatin receptor scintigraphy (SRS), were recently described with promising results.*
Methods. *In this study we correlated the results of preoperative EUS (n = 34) and SRS (n = 30) with operative findings in patients with histologically proven insulinoma (n = 20) or gastrinoma (n = 21).*
Results. *The sensitivity and positive predictive value (PPV) of EUS were respectively 77% and 94% for pancreatic tumors (insulinomas and gastrinomas), 40% and 100% for duodenal gastrinomas, and 58% and 78% for metastatic lymph nodes. The sensitivity and PPV of SRS for insulinoma were 60% and 100%, respectively. In patients with gastrinoma, the sensitivity and PPV of SRS were respectively 25% and 100% for pancreatic gastrinomas, 72% and 100% for duodenal gastrinomas or periduodenal metastatic lymph nodes, and 67% and 80% for liver metastasis. In patients with multiple endocrine neoplasia, neither one of the two techniques detected all tumors. Overall sensitivity of combined EUS and SRS was 89% for insulinoma (n = 9) and 93% for gastrinoma (n = 14).*
Conclusions. *EUS and SRS for gastrinomas and insulinomas should be considered as the initial preoperative imaging procedures and may render invasive procedures unnecessary for most patients. (Surgery 1998;124:1134-44.)*

From the Department of General and Endocrine Surgery, Department of Gastroenterology, Laboratory of Nuclear Medicine, and Department of Endocrinology, University Hospital, Lille, the Department of Gastroenterology, Hopital Saint Philibert, Lomme, and the Clinique Turin, Paris, France

THE CLINICAL MANAGEMENT OF insulin- and gastrin-secreting duodenopancreatic tumors remains a challenging issue.[1] Numerous tools such as intraoperative ultrasound, endoscopic transillumination, and quick hormone measurements are available to guide the operating surgeon, but preoperative localization of the tumor(s) remains of paramount importance for proper surgical strategy. The results of conventional morphologic imaging modalities including percutaneous ultrasound, computed tomography, angiography, and magnetic resonance imaging have remained disappointing during the last decade.[2,3] The best technique currently described is the functional localization of the tumor(s) by hormone measurement in the suprahepatic veins after sequential intra-arterial stimulation as proposed for gastrinoma by Imamura and Takahashi[4] and modified by Strader et al[5] for insulinoma. However, these techniques do not localize the tumor but rather regionalize the hypersecretion. They are invasive and demanding and remain poorly available.

A great breakthrough took place with the recent advent of 2 remarkable noninvasive localization studies: endoscopic ultrasonography (EUS)[6] and somatostatin receptor scintigraphy (SRS).[7] The outstanding results reported by the pioneering authors rapidly led to the widespread use of these 2 methods for imaging endocrine duodenopancreatic tumors,[7-10] especially for gastrinomas.[11-16] EUS was also proved of particular value for insulino-

Presented at the 19th Annual Meeting of the American Association of Endocrine Surgeons, Orlando, Fla, Apr 26-28, 1998.

Reprint requests: Professeur Charles Proye, Service de Chirurgie Générale et Endocrinienne, Clinique Chirurgicale Adulte Est, Centre Hospitalier et Universitaire de Lille, 1 Place de Verdun, 59037 Lille, France.

Copyright © 1998 by Mosby, Inc.

0039-6060/98/$5.00 + 0 **11/6/93109**

Table I. Endocrine dudodenopancreatic tumors operated in the Department of General and Endocrine Surgery, March 1998

Tumor type	*No.*	*%*	*No. of patients with MEN 1*
Insulinomas	60	43	8
Gastrinomas	48	35	9
Glucagonomas	8	6	6
Vipomas	6	4	3
Human pancreatic polypeptide tumors	4	3	1
Nonfunctional	13	9	0
Overall	139	100	27

mas.[15,16] The overall performances of these noninvasive procedures rapidly led to their wide acceptance.[17,18] Despite numerous reports the precise description of their outcome remains scarce and eventually contradictory in the literature.

In this study we report the correlation of pathologic findings with the preoperative results of EUS or SRS in patients operated on in our department since 1991 for proven gastrinoma or insulinoma.

MATERIAL AND METHODS

Patients. In this retrospective study we examined the medical records of 40 patients fulfilling the following criteria among 139 patients who underwent surgical exploration in the Department of Endocrine Surgery at the University Hospital of Lille for duodenopancreatic endocrine tumors (Table I): (1) a clinical and biologic diagnosis of insulinoma or gastrinoma confirmed by immunohistochemical analysis of the specimen and (2) preoperative imaging with EUS or SRS.

Imaging procedures. Somatostatin receptor scintigraphy was performed with indium 111-pentetreotide (Octreo Scan; Mallinckrodt, Bondoufle, France). The mean administered radioactivity amounted to 111 to 185 MBq. Planar images were acquired with a gamma camera Sophy DS7 (Sopha Medical, Buc, France) fitted with a medium energy and high resolution collimator. Spectrometry was calibrated on the 2 peaks of indium (173% ± 20% keV and 247% ± 20% keV). Planar anterior and posterior images of the thorax, abdomen, and pelvis were acquired 4 and 24 hours after administration of the tracer. Additional cranial profile images were obtained at 24 hours. A hepatic tracer (phytates-technetium 99m) was administered when an equivocal finding was observed on abdominal images at 24 hours. Single photon emission computed tomography was performed in some cases (360 degrees, 64 projections, 30 seconds for each step). Slices were reconstructed

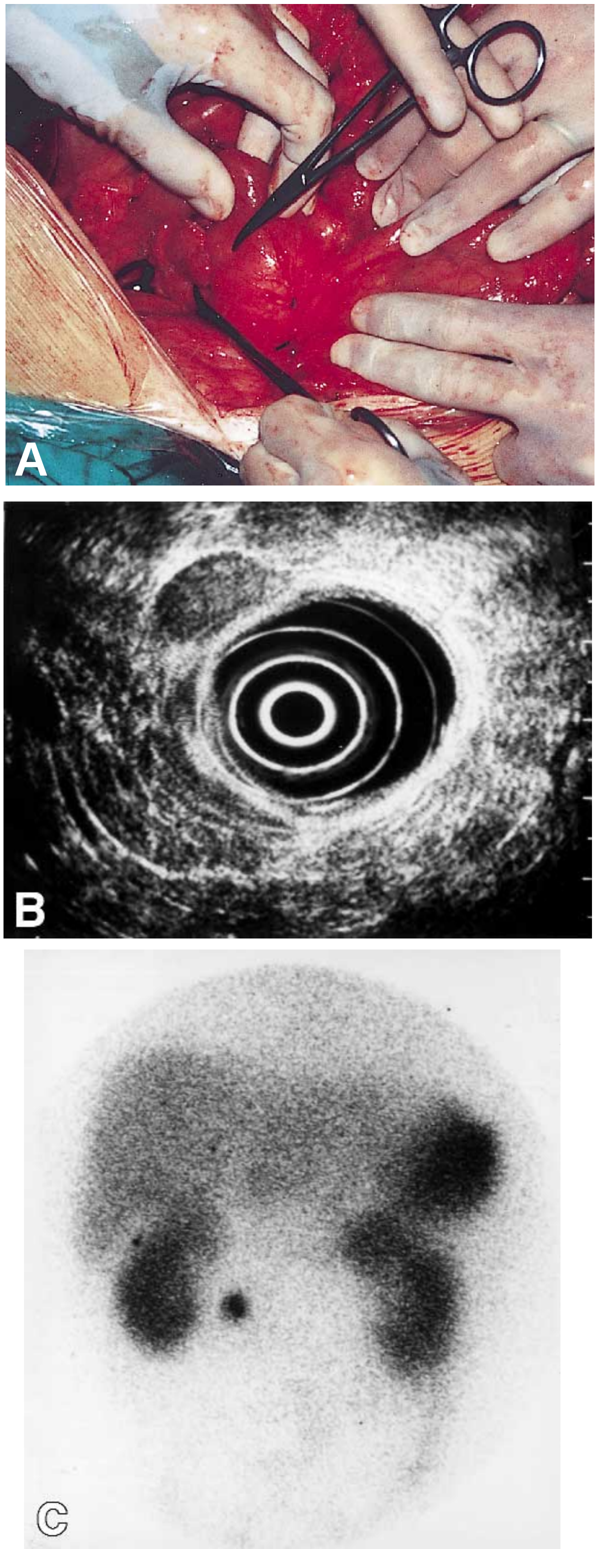

Fig 1. Forty-eight-year-old man with hypergastrinemia cured by the surgical excision of a 4-mm duodenal wall gastrinoma *(right forceps)* and an 11-mm metastatic periduodenal lymph node *(left forceps)* **(A)**. Both the primary lesion *(left)* and the node *(top left)* were preoperatively depicted by EUS **(B)**. SRS showed a pathologic uptake within the triangle area **(C).**

Table II. Correlation of operative findings with results of EUS or SRS in 20 patients with insulinoma

			Operative findings	
Patient no.	*Age-sex (y-M/F)*	*Clinical characteristics*	*Primary (size)*	*Metastasis (no.)*
I-34	49-M	Sporadic	Left pancreas (10 mm)	None
I-35	63-F	Sporadic	Head (11 mm)	None
I-36	31-M	Sporadic	Head (15 mm)	None
I-42	46-F	Sporadic	Left pancreas (20 mm)	None
I-43	76-F	MEN 1	Head (12 mm)and left pancreas (multiple)	Lymph node (1)
I-44	47-F	Sporadic	Head (5 mm)	None
I-46	43-F	Sporadic	Left pancreas (15 mm)	None
I-47	62-F	Sporadic	Left pancreas (8 mm)	None
I-48	44-F	MEN 1	Head (20 mm)	None
I-49	16-F	Sporadic	Head (14 mm)	
I-50	20-M	Sporadic	Left pancreas (15 mm)	None
I-51	53-F	Sporadic	Left pancreas (11 mm)	None
I-52	65-M	Sporadic	Left pancreas (20 mm)	None
I-53	59-F	MEN 1	Left pancreas (12 mm)	None
I-54	12-M	Sporadic	Left pancreas (10 mm)	None
I-55	65-F	Sporadic	Head (10 mm)	None
I-56	60-F	Sporadic	Left pancreas (8 mm)	None
I-58	35-F	Sporadic	Head (14 mm)	None
I-59	22-F	MEN 1	Head (5 mm) and left pancreas (multiple)	None
I-60	47-F	Sporadic	Left pancreas (15 mm)	Lymph node (3)

NA, Not available.

*True positive for 1 pancreatic tumor and false positive for node.

after back projection with a Parzen filter. Endoscopic ultrasonography was performed by 1 of 4 experienced operators (J. M. G., V. M., B. F., L. P.). With the patient under general anesthesia, the tests were performed with a forward viewing echoendoscope (Olympus, Tokyo, Japan) using ultrasound frequency of 7.5 or 12 MHz.

Operative procedure. Standardized surgical exploration was performed as previously described for insulinoma[19] and gastrinoma.[20] The operation started with exhaustive visual inspection and palpation from the inframesocolic area to the supramesocolic area, including the liver. A Kocher's maneuver was performed, and the lesser sac was opened from the hepatic to the splenic flexure. The posterior aspect of the tail was dissected free before performing the palpation of the whole gland and completed with intraoperative ultrasonography. In patients with insulinoma, any detectable pancreatic tumor was then resected by enucleation when deemed feasible or formal pancreatic resection. In patients with gastrinoma, a systematic node clearance of the triangle was carried out. Also in this latter setting, intraoperative endoscopy and duodenal transillumination were performed and followed by duodenotomy for intraluminal palpation. All tumors detected and potentially secreting gastrin were eventually resected. The completeness of the operation was assessed at the end of the procedure with intraoperative hormone measurements.[19,20]

Data analysis. To evaluate the results of EUS we specifically considered the description of the duodenal wall, the pancreas, and pathologic lymph nodes. For SRS, nonphysiologic hot spots were searched for in the gastrinoma triangle (periduodenal and pancreatic head area), the left pancreas, and the liver. For the specific analysis, a test was considered as a true positive when at least one lesion present in the considered region was detected. We considered as a false positive any imaging finding that was not confirmed by pathologic examination. For the overall analysis, we considered as a true positive a test that detected at least one lesion confirmed at operation in a given patient. The sensitivity was calculated according to the following formula: (Number of true positives/Number of patients who had the test). The predictive positive value was calculated according to the following formula: (True positives/[True positive + False positive]). Outcome of operation was assessed at 1 year after operation in all patients except for the most recent cases. Patients without clinical or biochemi-

Operation	EUS	SRS	Surgical outcome
Enucleation	Positive	NA	Cured
Enucleation	Negative	NA	Cured
Enucleation	Negative	Positive	Cured
Left pancreatectomy	Positive	NA	Cured
Left pancreatectomy, enucleation	NA	Positive	Cured
Enucleation	Positive	NA	Cured
Enucleation	Negative	Negative	Cured
Enucleation	Negative	Positive	Cured
Enucleation	Positive	Positive	Cured
Enucleation	Positive	NA	Cured
Left pancreatectomy	Positive	Negative	Cured
Enucleation	Positive	NA	Cured
Enucleation	Positive	NA	Cured
Enucleation	Positive	Negative	Cured
Enucleation	Positive	NA	Cured
Enucleation	Positive	NA	Cured
Left pancreatectomy	Positive	NA	Cured
Enucleation	Positive	Negative	Cured
Enucleation, left pancreatectomy	Positive*	Positive	Cured
Left pancreatectomy, adenectomy	Positive	Positive	Cured

cal evidence of recurrence / persistence of insulin or gastrin hypersecretion 1 year after operation were considered cured for the purpose of this study.

The results of continuous variables are expressed as the mean ± standard deviation.

RESULTS

The age of patients at the time of operation was 46.0 ± 2.7 years (range, 12 to 76 years). The female to male sex ratio was 1.5. The disease was sporadic in 35 patients and occurred in the setting of multiple endocrine neoplasia type 1 (MEN 1) in 5 patients. Twenty patients were confirmed during operation to have insulinoma (Table II), and 21 patients had gastrinoma (Table III). One of them (I-43) with MEN 1 had simultaneous hypergastrinemia and hyperinsulinemia and an intrapancreatic insulinoma, multiple intrapancreatic gastrinomas, and 1 gastrin-positive metastatic lymph node found at operation. Operative findings in the 40 patients are summarized in Table IV. All patients with insulinoma were biochemically cured after operation.

EUS was performed in 19 patients with insulinoma and 15 patients with gastrinoma. Table V summarizes the results obtained with this technique for pancreatic, duodenal, and lymph node tumors. Most intrapancreatic tumors were correctly detected with EUS except for 4 insulinomas with a size of 8 to 15 mm and 1 intrapancreatic 20 mm gastrinoma. In 1 case (G-48), EUS falsely indicated a 6-mm pancreatic tumor that was not confirmed during operation by palpation and intraoperative sonography. This patient was biochemically cured after the excision of a duodenal primary gastrinoma and 1 metastatic lymph node. Fig 1 depicts the typical aspect of a hypoechogenic tumor seen in 4 patients with submucosal dudodenal gastrinoma. EUS also missed duodenal gastrinomas measuring 8 to 15 mm in 6 patients, and its sensitivity was only 40% in that setting. EUS predicted the presence of metastatic lymph node in more than half of cases, as illustrated by Fig 2 in a patient with insulinoma. Undetected lymph nodes were situated within the gastrinoma triangle (n = 2) and around the splenic artery (n = 2). In 1 patient an incomplete EUS also failed to recognize a voluminous and apparently primary lymph node gastrinoma adjacent to the proximal jejunum. Nonspecific lymph nodes were falsely considered as metastatic by EUS in 2 patients.

The results of SRS are summarized in Table VI. SRS was performed in only 10 patients with insulinoma and was positive in 6 cases. The results in

Table III. Correlation of operative findings with results of EUS or SRS in 21 patients with gastrinoma

			Operative findings		
Patient No.	*Age-sex (y-M/F)*	*Clinical characteristic*	*Primary (size)*	*Metastasis (No.)*	*Operation*
G-16	43-F	Sporadic	Duodenum (15 mm)	Lymph node (3), liver (1)	Duodenectomy, metastasectomy
G-22	65-M	Sporadic	Left pancreas (20 mm)	Lymph node (multiple), liver (multiple)	Left pancreatectomy
G-23	14-M	Sporadic	None	Lymph node (1)	Duodenectomy
G-24	60-F	Sporadic	None	Liver (multiple)	Liver biopsies
G-25	53-F	Sporadic	None	Lymph node (2)	Left pancreatectomy
G-27	39-F	Sporadic	Duodenum (20 mm)	Lymph node (2)	Duodenectomy
G-29	45-M	Sporadic	Duodenum (6 mm)	None	Duodenectomy
G-31	58-F	Sporadic	Left pancreas (20 mm)	Lymph node (1)	Left pancreatectomy
G-32	56-M	Sporadic	duodenum (25 and) 15 mm)	Lymph node (2), liver (2)	Liver resection
G-33	64-F	Sporadic	Duodenum (25 mm)	None	Duodenectomy
G-34	56-M	Sporadic	Duodenum (20 mm)	None	Duodenectomy
G-38	40-F	Sporadic	None	Lymph node (1), liver (multiple)	Enucleation
G-41	48-M	Sporadic	Duodenum (4 mm)	Lymph node (1)	Duodenectomy
G-42	65-M	Sporadic	None	Lymph node (1)	Duodenectomy
G-43	45-M	Sporadic	Duodenum (7 mm)	None	Duodenectomy
I-43	76-F	MEN 1	Left pancreas (20 mm)	Lymph node (1)	Left pancreatectomy
G-44	30-M	MEN 1	None	Lymph node (1), liver (multiple)	Liver biopsies
G-45	41-F	Sporadic	Duodenum (8 mm)	None	Duodenectomy
G-46	33-F	Sporadic	Duodenum (3 mm), pancreas (multiple)	Lymph node (2)	Duodenectomy, enucleation, left pancreatectomy
G-47	37-F	Sporadic	None	Lymph node (6)	Duodenectomy
G-48	42-M	Sporadic	Duodenum (4 mm)	Lymph node (1)	Duodenectomy

NA, Not available.

*False positive.

these patients were not influenced by the location of the tumors within the pancreas or by their size (15 ± 5 mm vs 14 ± 2 mm, $P = .69$, Mann Whitney test). Twenty patients with gastrinoma had preoperative SRS. SRS did not show any uptake focus within the gastrinoma triangle in 5 cases. Three patients had an isolated duodenal gastrinoma (6 to 8 mm), and one had a 20-mm duodenal lesion associated with a metastatic lymph node. SRS also showed multiple liver lesions but failed to predict the presence of a metastatic periduodenal lymph node in 1 patient with MEN 1. SRS was negative in 3 of 4 patients with primary gastrinoma within the left pancreas. Tumors were multiple in 2 cases. In the third one, SRS correctly detected a metastatic periduodenal lymph node, but a 20-mm pancreatic primary tumor and liver metastasis did not uptake the tracer. Among the 6 patients found to have liver metastasis at operation, SRS did not show any pathologic hepatic uptake in 2 cases, despite positive magnetic resonance imaging. In only 1 case with previous gastric operation (G-48), a pathologic octreotide uptake was not confirmed at operation but corresponded to a nonspecific inflammatory focus within the gallblader area.

Overall results of EUS and SRS are summarized in Table VII. Nine patients with insulinoma and 14 patients with gastrinomas had both procedures before operation. In that setting, preoperative imaging depicted at least 1 pathologic finding in all cases except for 1 insulinoma and 1 gastrinoma. Pathologic tracer uptake was seen with SRS despite a negative EUS in 2 patients with insulinoma. SRS also successfully detected the intrapancreatic gastrinoma not depicted by EUS and the isolated jejunal lymph node in patient G-42. In patients with negative SRS, EUS correctly depicted 3 intrapancreatic insulinomas and 3 isolated intraduodenal gastrinomas.

Multiple lesions were found at operation in 16 patients, 2 with insulinomas, 13 with gastrinomas, and 1 with both types of tumors. Three of them had MEN 1. Preoperative imaging with EUS or SRS accu-

EUS			SRS			
Duodenum	*Pancreas*	*Node*	*Triangle*	*Tail*	*Liver*	*Surgical outcome*
Negative	Negative	Positive	Positive	Negative	Positive	Not cured
Negative	Negative	Negative	Positive	Negative	Negative	Not cured
Negative	Negative	Negative	NA	NA	NA	Cured
NA	NA	NA	Negative	Negative	Negative	Not cured
NA	NA	NA	Positive	Negative	Negative	Not cured
Negative	Negative	Negative	Negative	Negative	Negative	Not cured
Positive	Negative	Negative	Negative	Negative	Negative	Cured
Negative	Positive	Negative	Negative	Positive	Negative	Cured
Negative	Negative	Positive	Positive	Negative	Positive	Not cured
NA	NA	NA	Positive	Negative	Negative	Cured
Positive	Negative	Negative	Positive	Negative	Negative	Cured
NA	NA	NA	Positive	Negative	Positive	Not cured
Positive	Negative	Positive	Positive	Negative	Negative	Cured
Negative	Negative	Negative	Positive	Negative	Negative	Cured
Negative	Negative	Positive*	Negative	Negative	Negative	Cured
NA	NA	NA	Positive	Negative	Negative	Cured
NA	NA	NA	Negative	Negative	Positive	Not cured
Positive	Negative	Negative	Negative	Negative	Negative	Cured
Negative	Positive	Positive	Positive	Negative	Negative	Not cured
Negative	Negative	Positive	Positive	Negative	Negative	Cured
Negative	Positive*	Positive	Positive	Negative	Positive*	Cured

rately detected all lesions in only 3 of these patients, all with sporadic disease. EUS showed all lesions of patient I-60 including 3 metastatic lymph nodes and both primary duodenal gastrinoma and 1 invaded lymph node in patient G-41. SRS showed 1 hot spot within the triangle area and multiple uptake liver foci in patient G-38 with lymph node and liver metastasis.

DISCUSSION

Preoperative imaging of islet cell duodenopancreatic tumors remains the ultimate challenge for abdominal imaging procedures. Numerous previous studies have already clearly indicated the value of EUS and SRS in that setting. In this study, we report the performances of these techniques for both insulinomas and gastrinomas.

All patients with proven insulinoma or gastrinoma since the introduction of EUS and SRS at our institution (1991) were included. This retrospective study can therefore be assumed to accurately reflect the outcome of these techniques in unselected patients. Outcome after operation confirmed the completeness of resection in all patients with insulinoma and 57% of patients with gastrinoma. Lymph node or liver metastatic spread of the disease was identified at operation in all patients in whom hypergastrinemia persisted. In contrast with many previous reports including nonoperated patients,[13,21] the comprehensive surgical exploration and pathologic confirmation in all patients of this study allowed us to accurately correlate the imaging findings to the clinical disease, especially for gastrinoma.

When performed by experienced endoscopists, EUS is an accurate morphologic tool able to predict both the site and the size of most neuroendocrine tumors.[7] As illustrated in Fig 2, this technique may prove of particular value, guiding the surgeon toward both the primary tumor and the metastatic lymph nodes. The excellent overall sensitivity of EUS for intrapancreatic tumors[7] was confirmed in our patients, both for insulinomas (79%) and for the less frequent intrapancreatic gastrinomas

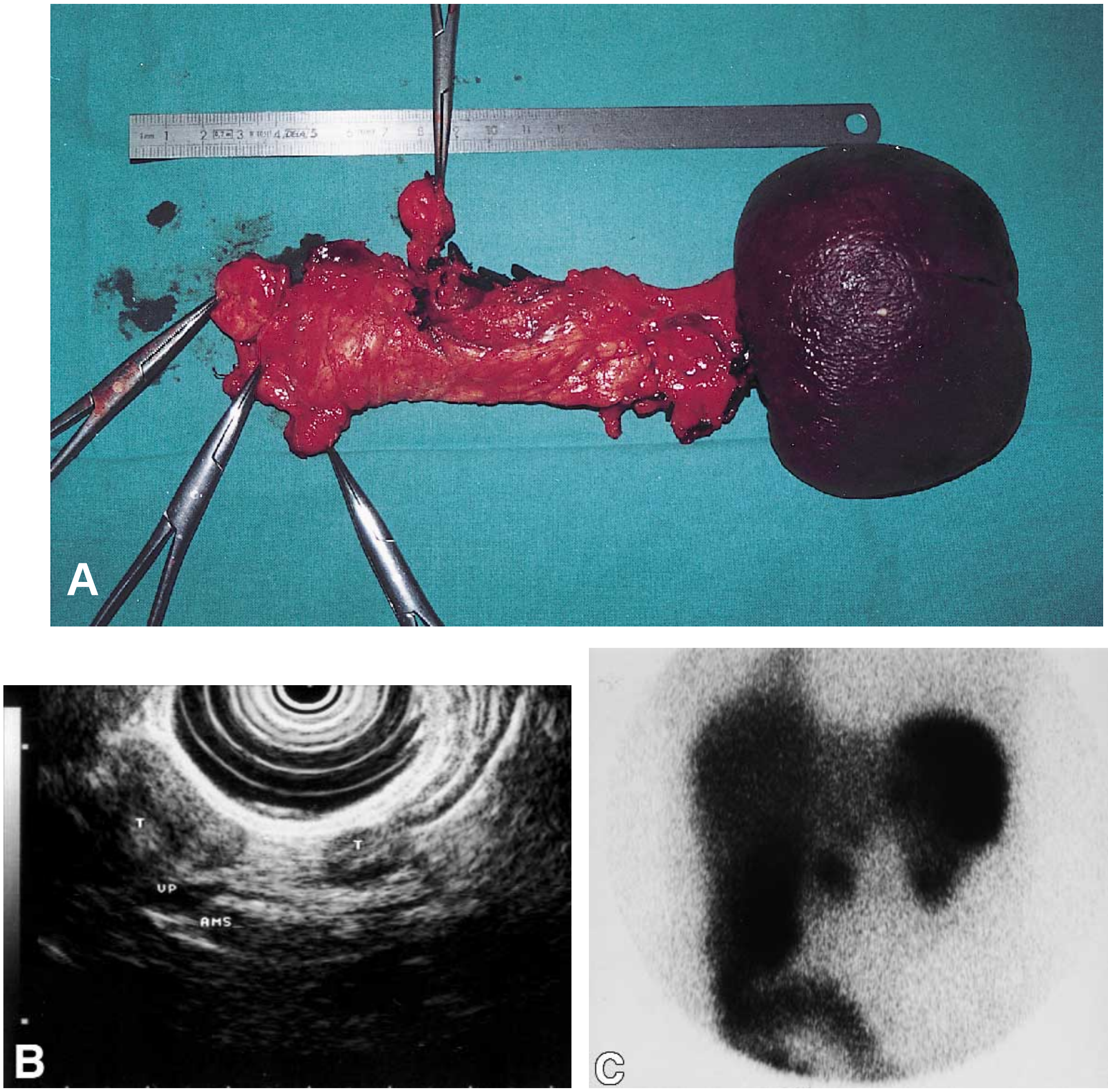

Fig 2. Fifty-nine-year-old woman with hyperinsulinemia cured by the surgical excision of a 15-mm pancreatic neuroendocrine tumor and 3 metastatic peripancreatic lymph nodes **(A)**. Both the primary lesion and the nodes were preoperatively depicted by EUS **(B)**; *T* (left-hand side), tumor; *T* (right-hand side), lymph node; *VP*, portal vein; *AMS*, superior mesenteric artery. SRS showed pathologic uptakes within the triangle area and the left pancreatic area **(C)**.

(67%). All 5 intrapancreatic tumors missed by EUS, including 2 pedunculated and 1 juxta-ampullary insulinomas, another insulinoma embedded in an area of pancreatitis in a redo case, and 1 pancreatic gastrinoma embedded within the splenic hilum, were successfully detected intraoperatively by palpation or intraoperative ultrasonography. On the other hand, the positive predictive value of EUS was not absolute in our experience, underlining the lack of specificity of sonography within the pancreas. Pancreatic resection for a nonpalpable intrapancreatic tumor diagnosed by preoperative EUS would therefore appear unwise without confirmation by intraoperative ultrasonography.[22]

One of the more challenging tasks for preoperative imaging is the detection of the small duodenal gastrinomas. In our series, such lesions were found in 52% of patients, as compared with 19% of intrapancreatic tumors. These figures, in accordance with the most recent surgical series,[12,14] may reflect the accuracy of intraoperative maneuvers such as transillumination and dudodenotomy performed in most of our patients to detect these frequent but elusive lesions. EUS correctly detected 40% of the duodenal gastrinomas confirmed during operation, showing a typical hypoechoic, round shaped, and well-limited tumor within the submucosa of the duodenal wall (Fig 2). In 3 cases, EUS

Table IV. Operative findings in 21 patients with gastrinoma and 20 patients with insulinoma

	Gastrinoma		*Insulinoma*	
Localization	*n*	*%*	*n*	*%*
Primary tumor(s)				
Duodenum	11	52	0	0
Pancreas	4	19	20	100
None	7	33	0	0
Metastasis				
Lymph node	14	67	2	10
Liver	6	29	0	0
None	6	29	18	90

Table V. Results of preoperative EUS

Tumor type	*Patients (n)*	*True positive (n)*	*False negative (n)*	*False positive (n)*	*Sensitivity (%)*	*Positive predictive value (%)*
Insulinoma						
Pancreas	19	15	4	0	79	100
Lymph node	1	1	0	1	100	50
Gastrinoma						
Duodenum	10	4	6	0	40	100
Pancreas	3	2	1	1	67	67
Lymph node	11	6	5	1	55	86
Overall						
Pancreas	22	17	5	1	77	94
Lymph node	12	7	5	2	58	78

Table VI. Results of preoperative SRS

Tumor type	*Patients (n)*	*True positive (n)*	*False negative (n)*	*False positive (n)*	*Sensitivity (%)*	*Positive predictive value (%)*
Insulinoma	10	6	4	0	60	100
Gastrinoma						
Triangle	18	13	5	0	72	100
Left pancreas	4	1	3	0	25	100
Liver	6	4	2	1	67	80

Table VII. Overall sensitivity of preoperative EUS and SRS

Test	*Insulinoma*	*Gastrinoma*
EUS	15/19 (79)	11/15 (73)
SRS	6/10 (60)	15/20 (75)
Combined	8/9 (89)	13/14 (93)

Data are expressed as number of true positives/tests performed (percent).

Table VIII. Outcome of preoperative imaging in 12 patients with gastrinoma cured by surgery

Test	*n*	*Positive*	*Negative*
EUS	10	7	3
SRS	11	8	3
Combined	9	8*	1

*Both procedures were positive in 5 patients and 1 procedure was positive in 3 patients.

was the only positive preoperative imaging procedure in patients who were cured by surgical resection of a unique duodenal gastrinoma. The preoperative detection of these small intraduodenal tumors by EUS remains extremely demanding, especially when their size is less than 10 mm.[3,12] As illustrated in 6 of our patients, endoscopic transillumination and duodenotomy remain mandatory during operation for proper duodenal exploration and discovery of small submucosal tumors that may be overlooked by EUS. Conversely we recently operated on a patient with equivocal biologic diagnosis of Zollinger-Ellison syndrome 3 years after a previous duodenotomy for bleeding ulcer in whom

EUS showed a typical duodenal submucosal lesion (not shown). Duodenotomy failed to detect any tumor except a nonspecific granuloma of the duodenal wall from previous operation.

Most insulinomas are benign, but lymph node invasion is frequent in patients with gastrinoma and occurred in two thirds of our patients. EUS detected enlarged lymph nodes, strongly suggesting their malignant nature, in 58% of patients during operation. The more distant nodes such as those situated within the splenic artery loops are less likely to be detected than their counterparts within the triangle. Hopefully these are also more readily accessible to intraoperative palpation. This study confirms that EUS should include a comprehensive and extensive exploration of the entire duodenum and the first jejunal loop to increase the likelihood of detection of pathologic lymph nodes and pedunculated pancreatic tumors.[15] Once again, positive predictive value of EUS for invaded lymph nodes was not absolute. As already suggested,[12] EUS can hardly distinguish nonspecific hypertrophic lymph nodes with tumoral metastasis. For the lesion situated within 6 cm from the duodenum and measuring at least 5 mm, endosonography-guided fine-needle aspiration biopsy may increase the positive predictive value of the test near 100%.[23]

The value of preoperative SRS has been well demonstrated in various types of neuroendocrine tumors, especially for carcinoid tumors[8,9] and gastrinomas.[13,14,16,21] Octreoscan uptake is significantly less frequent in insulinomas.[16] In our study, SRS was nevertheless positive in 60% of patients with insulinoma, including a metastatic lymph node in 1 case. The results of SRS did not depend on size or location of the tumor, and the more likely explanation for this selective octreotide uptake in insulin-secreting tumors has been shown to be the presence of type 2 somatostatin receptors.[17] It is remarkable that although the sensitivity of SRS appeared more limited for insulinoma, it accurately located an intrapancreatic lesion in 2 patients with false-negative EUS results.

One of the major advantages of SRS is certainly its high specificity for neuroendocrine tumors.[24] Although the design of our study did not allow us to address the specificity of SRS, only one positive uptake was not confirmed to correspond to any tumoral process at operation. False-positive results of SRS remain possible, but we found that overall positive predictive value of SRS may exceed 95%. It is also noteworthy that a positive SRS indicates the presence of somatostatin receptors in the tumor and is highly suggestive of efficacy of hormonal inhibition by octreotide therapy.[11]

Although extra-abdominal metastases are rare in gastrinomas and were not encountered in our patients, SRS may prove of particular value to detect lesions outside the abdomen such as in the lung.[21] Nevertheless, false-positive scans have been reported for granulomatous diseases.[10] We also have seen a false-positive scan in a patient with factitious organic hypoglycemia and a lung abscess (not shown). SRS is also generally considered to be an excellent tool to detect liver metastasis.[10,17] A sensitivity greater than 90% has been reported for hepatic lesions in patients with advanced disease not accessible to surgery.[13,21] The value of preoperative SRS to detect occult metastasis in unselected patients with gastrinoma is not known and is probably much inferior. Because of the background caused by physiologic hepatic uptake, the detection of small foci within the liver is unlikely. In 2 of our patients, SRS failed to detect liver lesions that were seen with conventional morphologic imaging and confirmed at operation. Conversely in another case (G-44), a false-positive liver uptake obscured a positive node in the triangle.

If we focus on the most challenging subset of patients with gastrinoma, ie, those who were cured by primary surgery, 10 of 12 had at least 1 true-positive preoperative imaging study with either EUS or SRS (Table VIII). Among those, 2 nonmetastatic duodenal wall microgastrinomas of 6 and 8 mm in size were identified by positive EUS.

Therefore we consider positive preoperative imaging of such minute tumor(s) to be almost a prerequiste for surgery of sporadic gastrinomas, hopefully for cure.

We conclude that noninvasive preoperative imaging of insulinomas and gastrinomas by combined EUS and SRS should replace all other invasive imaging procedures. Other tests, such as intra-arterial stimulation tests, should be performed only if EUS and SRS are negative.

REFERENCES

1. Proye C. Endocrine tumors of the pancreas: an update. Aust NZ J Surg 1998;68:90-100.
2. Proye C, Boissel P. Preoperative imaging versus intraoperative localization of tumors in adult surgical patients with hyperinsulinemia: a multicenter study of 338 patients. World J Surg 1984;12:685-90.
3. Doppman JL. Pancreatic endocrine tumors: the search goes on. N Engl J Med 1992;326:1770-3.
4. Imamura M, Takahashi K. Use of selective arterial secretin injection test to guide surgery in patients with Zollinger-Ellison syndrome. World J Surg 1993;17:433-8.
5. Strader DB, Doppman JL, Orbuch M, Jensen RT, Metz DC. Functional localization of pancreatic tumors. In: Mignon M, Jensen RT, editors. Endocrine tumors of the pancreas. Basel (Switzerland): Karger; 1995. p 282.

6. Rosch T, Lightdale CJ, Botet JF, Boyce GA, Sivak MV, Yasuda K, et al. Localization of pancreatic endocrine tumors by endoscopic ultrasonography. N Engl J Med 1992;326:1721-6.
7. Lamberts SWJ, Bakker WH, Reubi JC, Krenning EP. Somatostatin-receptor imaging in the localization of endocrine tumors. N Engl J Med 1990;323:1246-9.
8. Carnaille B, Nocaudie M, Pattou F, Huglo D, Deveaux M, Marchandise X, et al. Scintiscans and carcinoid tumors. Surgery 1994;116:1118-22.
9. Kvols LK, Brown ML, O'Connor MK, Hung JC, Hayostek RJ, Reubi JC, et al. Evaluation of a radiolabeled somatostatin analog (I-123 octreotide) in the detection and localization of carcinoid and islet cell tumors. Radiology 1993;187:129-33.
10. Meko JB, Doherty GM, Siegel BA, Norton JA. Evaluation of somatostatin-receptor scintigraphy for detecting neuroendocrine tumors. Surgery 1996;120:975-84.
11. Nocaudie-Calzada M, Huglo D, Deveaux M, Carnaille B, Proye C, Marchandise X. Iodine-123-tyr-3-octreotide uptake in pancreatic endocrine tumors and in carcinoids in relation to hormonal inhibition by octreotide. J Nucl Med 1994;35:57-62.
12. Ruszniewski P, Amouyal P, Amouyal G, Grange JD, Mignon M, Bouche O, et al. Localization of gastrinomas by endoscopic ultrasonography in patients with Zollinger-Ellison syndrome. Surgery 1995;117:629-35.
13. Gibril F, Reynolds JC, Doppman JL, Chen CC, Venzon DJ, Termanini B, et al. Somatostatin receptor scintigraphy: its sensitivity compared with that of other imaging methods in detecting primary and metastatic gastrinomas—a prospective study. Ann Intern Med 1996;125:26-34.
14. Cadiot C, Lebtahi R, Sarda L, Bonnaud G, Marmuse JP, Vissuzaine C, et al. Preoperative detection of duodenal gastrinomas and peripancreatic lymph nodes by somatostatin receptor scintigraphy. Gastroenterology 1996;111:845-54.
15. Thompson NW, Czako PF, Fritts LL, Bude R, Bansal R, Nostrant TT, et al. Role of endoscopic ultrasonography in the localization of insulinomas and gastrinomas. Surgery 1994;116:1131-8.
16. Zimmer T, Stölzel U, Bäder M, Koppenhagen K, Hamm B, Buhr H, et al. Endoscopic ultrasonography and somatostatin receptor scintigraphy in the preoperative localisation of insulinomas and gastrinomas. Gut 1996;39:562-8.
17. Modlin IM, Tang LH. Approaches to the diagnosis of gut neuroendocrine tumors: the last word (today). Gastroenterology 1997;112:583-90.
18. Wiedenmann B, Jensen RT, Mignon M, Modlin M, Skogseid B, Doherty G, et al. Preoperative diagnosis and surgical management of neuroendocrine gastroenteropancreatic tumors: general recommendations by a consensus workshop. World J Surg 1998;22:309-18.
19. Proye C, Pattou F, Carnaille B, Lefebvre J, Decoulx M, D'Herbomez M. Intraoperative insulin measurement during surgical management of organic hypoglycemia. World J Surg 1998. In press.
20. Proye C, Pattou F, Carnaille B, Paris JC, D'Herbomez M, Marchandise X. Intraoperative gastrin measurement during surgical management of gastrinomas. World J Surg 1998;22:643-50.
21. de Kerviler E, Cadiot G, Lebtahi R, Faraggi M, LeGuludec D, Mignon M. Somatostatin receptor scintigraphy in forty-eight patients with the Zollinger-Ellison syndrome. Eur J Nucl Med 1994;21:1191-7.
22. Norton JA. Surgical treatment of islet cell tumors with special emphasis on operative ultrasound. In: Mignon M, Jensen RT, editors. Endocrine tumors of the pancreas: recent advances in research and management. Basel: Karger; 1995. p. 309.
23. Wiersema MJ, Vilmann P, Giovannini M, Chang KG, Wiersema LM. Endosonography-guided fine needle aspiration biopsy: diagnostic accuracy and complication assessment. Gastroenterology 1997;112:1087-95.
24. van Eijck CHJ, Lamberts SWJ, Lemaire LCJ, Jeekel H, Bosman FT, Reubi JC, et al. The use of somatostatin receptor scintigraphy in the differential diagnosis of pancreatic duct cancers and islet cell tumors. Ann Surg 1996;224:119-24.

DISCUSSION

Dr Norman W. Thompson (Ann Arbor, Mich). We have used EUS for both insulinoma and gastrinoma localization for the last 4 years. It is our first test. There are, however, several differences between your experience and ours. Your sensitivity for pancreatic gastrinomas was only 67%. Virtually all pancreatic gastrinomas are larger than 2 cm. I am therefore surprised that your sensitivity was not 100%. EUS will detect virtually all tumors in the head that are 1 cm or larger and many smaller than that. Can you explain that? What did you find at operation in those patients who had negative EUS for pancreatic gastrinomas?

Dr Proye. Endoscopic ultrasonography is a magnificent tool, but it has limitations. It is a wonderful tool to help to dig out the tumors that are embedded in the pancreatic parenchyma, but it is not an excellent tool for pancreatic tumors bulging on the edge of the pancreas or abutting it. Fortunately, those tumors that you cannot demonstrate by endoscopy are those most likely to be felt by the surgeon. In fact, we had only 3 cases of pancreatic gastrinomas. The one we missed was a 2-cm malignant tumor in the distal part of the tail, virtually encased in the splenic hilum.

Dr Thompson. I would agree with you if our only failures in the past 4 years with insulinomas had been pedunculated tumors that were outside the pancreatic parenchyma. They are the easiest ones for the surgeon to find at operation. However, whenever we have found pedunculated gastrinomas, they were not primary pancreatic tumors but metastatic lymph nodes under the capsule or the sheath of the pancreatic head. These patients still had an underlying primary duodenal tumor.

I have one other question. It is amazing that you detected so many duodenal tumors by EUS. We infrequently see the duodenal tumors with EUS, and I would not rely on EUS for detecting those tumors. The vast majority of small tumors are not detectable. We make a presumptive diagnosis of duodenal gastrinoma when the pancreas is negative by EUS and the biochemical diagnosis has been established by a positive secretin test. The next paper will emphasize that, although occasionally you will encounter one elsewhere, 99% of all extrapancreatic gastrinomas will be in the duodenum. I don't know whether any other tests are really necessary. Will you proceed with an operation if your EUS is negative for your pancreas and you don't see anything in the duodenum? What do you do next? Do you do the Imamura arterial stimulation test or explore?

Dr Proye. I don't routinely do the Imamura test, even when EUS is negative. In the case you discussed, I would operate and explore the duodenum. But it is debatable.

Dr Thompson. One other question regarding your use of SRS. We found it of little help in either insulino-

ma or gastrinoma to detect the primary tumors. I noticed that the test was positive in 60% of your insulinomas. All of those would likely have been detected by EUS in our experience, and we would not have done a scan. With gastrinoma, our scans will pick up the large metastatic nodes and liver disease, but they virtually never detect the small primary tumors in the duodenum. What is your experience with SRS in detecting primary duodenal tumors? What is the sensitivity in your experience?

Dr Proye. Forty percent.

Dr Thompson. Those are good sized duodenal tumors. They are larger than the ones we see in most of our patients.

Dr Proye. They were between 4 and 25 mm.
